# Long‐Term Impact of Urban Areas on Meteorological Conditions Over Central Europe

**DOI:** 10.1111/nyas.70069

**Published:** 2025-09-28

**Authors:** Anahí Villalba‐Pradas, Jan Karlický, Peter Huszár, Michal Žák, Tomáš Halenka

**Affiliations:** ^1^ Department of Atmospheric Physics, Faculty of Mathematics and Physics Charles University Prague Czech Republic

**Keywords:** cloud cover, convection, microphysics, precipitation, UHI, UMI, urban

## Abstract

The impact of urban areas on relevant meteorological variables, especially on temperature and wind speed, is well‐known, and several studies have evaluated this effect. However, fewer of them have focused on the effect of urban areas on cloud cover and precipitation. To evaluate this, a total of 15 simulations were performed using the Weather Research and Forecasting model on a Central European domain at 9−km horizontal resolution. The novelty of this study is the number of cities considered and ensembles used over a 10‐year period including not only the impacts of the urban scheme but also the effects of two other relevant parameterizations, namely, convection and microphysics, on the representation of the urban meteorology island (UMI) variables with a special focus on less studied variables, that is, cloud cover and precipitation. Our results show that changes in temperature and specific humidity are mostly sensitive to the urban scheme selected, while changes in precipitation and cloud cover are more sensitive to the parameterization of convection and microphysics. The cloud cover strongly depends on the convection and microphysics schemes, especially in summer (JJA), although the impacts of the different parameterizations depend on the selected city. Despite differences observed among cities, cloud cover increases over urban areas during the afternoon and evening and decreases during the morning and night, especially in JJA. The selection of both convection and microphysics schemes plays an important role in the simulated precipitation. In winter (DJF), no significant variation between simulations is observed. On the other hand, precipitation is enhanced over urban areas during JJA. This study highlights the importance of using model ensembles and a number of cities when evaluating the urban heat island and UMI meteorological values, as large differences exist between the different setups and selected cities.

## Graphical abstract

This study evaluates the impacts of the urban, convection and microphysics schemes on the representation of cloud cover and precipitation over urban areas using ensembles over a 10‐years period. Changes in precipitation and cloud cover are more sensitive to the parameterization of convection and microphysics, although their impact is city‐dependent. In general, cloud cover and precipitation increase over urban areas during the afternoon and evening especially in JJA.

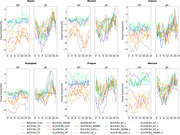

## 1 Introduction

Currently, more than half of the global population lives in cities, and it is expected that this figure will increase by 12 % over the next three decades [1]. In addition, it is well‐known that urban areas experience higher temperatures than their rural counterparts, the so‐called urban heat island (UHI) [2], which has a negative impact on human lives and activities [3, 4]. Therefore, it is crucial to improve our current knowledge of the main features of urban climates and their differences with the surrounding rural areas. The community, in general, and policymakers, in particular, will benefit from it as this information will help them adopt appropriate mitigation and adaptation strategies [5, 6].

Numerous studies have analyzed the impact of urban areas on temperature since the formulation of the UHI. This includes the use of observational data [7, 8, 9, 10], the introduction of empirical relations in temperature difference between the city centers and surroundings [7, 11, 12, 13], and the use of models (numerical weather prediction, or NWP, and regional climate models, or RCMs) [14, 15, 16, 17, 18, 19] whose horizontal resolution and representation of urban canopies have improved with the increase in computational power. More recently, several studies have been performed using artificial intelligence and machine learning [20, 21, 22, 23, 24].

Nevertheless, temperature is not the only meteorological variable affected by urban areas. Numerous studies have shown urban canopies' effect on wind flow [25, 26, 27, 28] and the boundary layer structure [8, 25, 29, 30], which further affect the dynamics and concentration of pollutants and thus air quality over these areas [18, 31, 32, 33, 34, 35, 36, 37, 38]. Despite being less studied, urban canopies also impact the distribution of cloud cover and precipitation as well as the specific humidity. In that sense, urban areas tend to increase convection [39, 40, 41], as well as cloud cover [42, 43] and precipitation during the summertime [44, 45, 46, 47], while decrease specific humidity, primarily during the same period [48, 49]. Moreover, it has been observed that the cloud base height over urban regions during summer and daytime is higher compared to the values in the vicinity, and that optically thicker clouds are more frequent over urban areas probably due to the enhancement of convective activity [50]. Regarding precipitation, studies showed an increase in downtown and downwind areas in big cities [51, 52, 53], but the influence of urban areas on precipitation extremes is not fully understood. Some studies showed increased precipitation extremes over urban areas [54, 55], while others did not find a significant impact [56, 57]. More recently, a study has shown that the impact of urbanization on extreme hourly precipitation in China depends on the location within the country, although authors showed a general increase in diurnal cycles [58]. In the evaluation of the impact of urban areas on cloud cover and precipitation, it is important to account for the presence of aerosols, which are responsible for the evolution of mixed clouds to deep clouds and thus affect precipitation. This impact of aerosol–cloud interactions on precipitation has been observed in different studies [59, 60].

Each of the aforementioned urban‐induced changes in relevant meteorological values led to the formulation of new concepts that could account for such effects—for example, the urban cool island [10] or the dry island [61, 62, 63, 64]. More recently, Karlický et al. [49] introduced a new definition, the so‐called urban meteorology island (UMI), that includes all the “one‐variable” islands other than the UHI, highlighting the different meteorological conditions in the urban areas to their surrounding rural parts.

The expected features of the UHI and the processes leading to it are qualitatively well‐represented in models. However, different models and different configurations within each model produce different magnitudes or even different signs of relevant UMI components. Therefore, the simulated UMI strongly depends on the way the models represent the most relevant processes leading to it. In this regard, parameterizations of the subgrid‐ scales process play an important role [49].

This study focuses on the impact that different urban canopy treatments, convection, and microphysics parameterizations have on the representation of the UHI and the different components of UMI using an RCM. Very few studies focus on the long‐term impact on cloud and precipitation microphysics in terms of impact on rain and cloudiness, and thus, we selected these two latter parameterizations as important factors that determine the impact of urban land.

The manuscript is organized as follows: the second section presents the model used, setup, domain, and driven data together with the observations used for evaluating simulation outputs. This section also includes a description of the methodology used throughout the manuscript. Next, the third section presents the results, starting with the evaluation of simulation outputs against observations for temperature and specific humidity. This section continues with an assessment of the impact of the selected parameterizations on the components of the UMI, first showing spatial differences during winter (DJF) and summer (JJA), and then differences in the diurnal values for the city center and the vicinity of a number of large cities within the domain. In the fourth section, results are discussed, and the last section closes the manuscript with the main conclusions.

## 2 Model and Data

In this study, simulations were conducted using one meteorological model, namely, the Weather Research and Forecasting model (WRF [65]). Outputs were validated using station‐based data from the European Climate Assessment and Dataset (ECAD [66]) for several European cities, as well as station data from the Czech Hydrometeorological Institute (CHMI) [67] in the case of Prague. The next subsections provide a more detailed explanation of the model setup, the observational data, and the methodology to evaluate the results obtained.

### 2.1 Model Configuration

**FIGURE 1:**
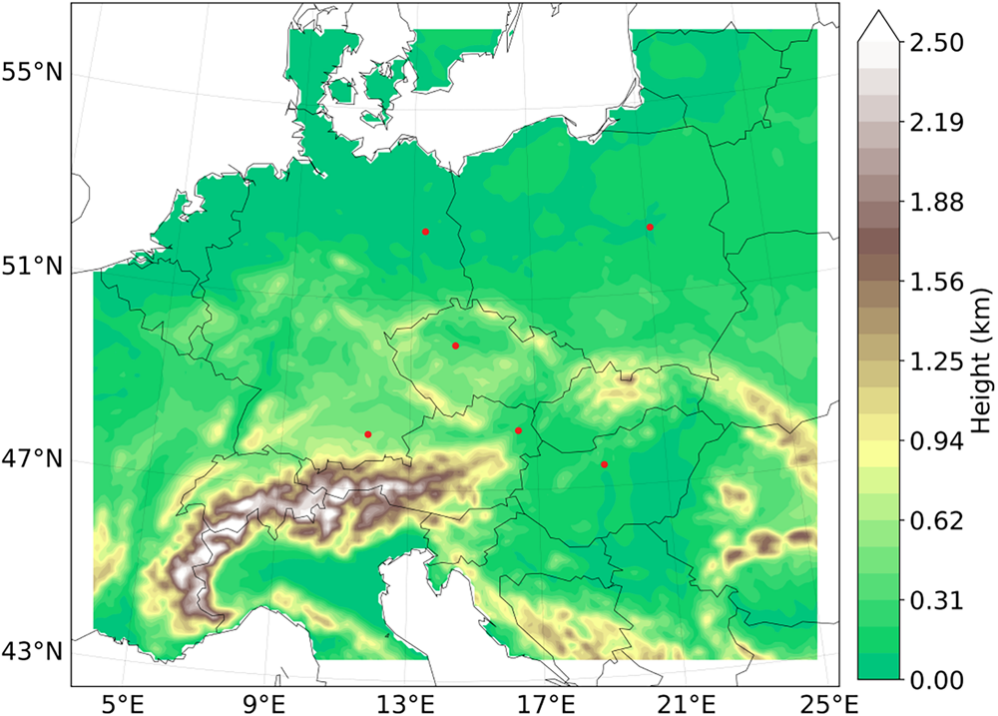
Model domain at a 9−km horizontal resolution showing model orography (km) in the Lambert Conformal projection including the location of the cities analyzed (Berlin, Budapest, Munich, Prague, Vienna, and Warsaw) in red dots.

WRF model was used in version 4.5.0 and was driven by the ERA5 reanalysis data [68]. The static geographic data used are those included in WRF, apart from the values of the land‐use fields. These are replaced by data derived from the CLC 2012 version of CORINE Land Cover data [69]. Figure SA.1 shows the land use for the simulated domain. The urban canopy parameters are modified following Karlický et al. [18]. The domain, centered over Prague (Czech Republic), covers Central Europe and spans a total of 190 × 166 grid points with 9−km horizontal resolution (Figure 1). The simulated period covers 10 years, from 2008 to 2017, to ensure the results' robustness. December 2007 was used as spin‐up.

WRF model offers a wide range of model schemes for different atmospheric processes. However, there are some restrictions on the combination of schemes. Considering this and the goal of this study, the schemes chosen for all the simulations are the Noah land surface model [70] with dominant land use to represent land surface exchange processes, the Rapid Radiative Transfer Model for general circulation models (RRTMG [71]) for the radiative transfer, the Eta Model [72] to parameterize the surface layer (SFL), and the BouLac planetary boundary layer (PBL) scheme [73] in the boundary layer processes representation. Only the schemes used in the description of convection, microphysics, and urban physics differ in the dedicated WRF simulations. The options used in the convection parameterization include the Grell 3D (G3D) [74], Grell−Freitas (GF) [75], and new Tiedtke (NT) [76] schemes. All of them are mass flux schemes that include both shallow and deep convection. In addition, the Grell−Freitas and the new Tiedtke schemes include momentum transport, and Grell 3D and Grell−Freitas include radiation interaction. Differences between the Grell‐type schemes and the new Tiedtke lie in the fact that the former are ensemble schemes, that is, use a multiclosure (based on quasi‐equilibrium, moisture convergence, convective available potential energy [CAPE] removal, or cloud‐base ascent), multi‐parameter, ensemble method with typically 144 subgrid members, while the latter uses a CAPE removal closure. Microphysics is parameterized with the Purdue Lin scheme [77], WRF Double Moment 6‐class (WDM6) [78], or P3 [79] schemes. Each scheme has a different level of complexity, being the Purdue Lin the least complex and the P3 the most. Both the Purdue Lin and the WDM6 scheme predict the mixing ratios of six water species (i.e., water vapor, cloud droplets, rain, cloud ice, graupel, and snow), while the P3 scheme is not based on fixed ice types but instead includes a single ice category that represents a combination of ice, snow, and graupel. Moreover, the WDM6 scheme includes double‐moment rain as well as a prognostic variable of cloud condensation nuclei, and the P3 contains prognostic arrays for rimed ice mass and rimed ice volume, and single‐moment rain and ice [80]. Last, the urban physics is represented through two different schemes, namely, the single‐layer urban canopy model (SLUCM [81]) and a bulk parameterization. In the former, urban street canyons are considered infinite, all buildings have the same characteristics, and takes into account the three‐dimensional nature of urban surfaces. The scheme considers the drag effect of the canyon and uses the surface energy budget as a base to calculate the surface temperature of roads, walls, and roofs. On the other hand, the bulk parameterization does not distinguish between street canyons and buildings but instead considers the same values of volumetric heat capacity, thermal conductivity, roughness length, and albedo for the whole urban domain. Yet, another configuration is included called NU, meaning no urban. This configuration uses the aforementioned bulk parameterization, but all urban areas in the domain are considered rural, that is, the domain does not have any urban area. In this case, the urban land use fraction for all cities was replaced by nonirrigated cropland (211 in CORINE) and pastures (231 in CORINE), meaning land use category 2 in USGS. In total, there are 15 different combinations presented in Table 1.

**TABLE 1:** Weather Research and Forecasting (WRF) model simulation setups.

Simulation No.	Simulation	Urban model	Convection	Microphysics
1	NU_G3D	bulk no urban	Grell 3D	Purdue Lin
2	NU_GF	bulk no urban	Grell−Freitas	Purdue Lin
3	NU_NT	bulk no urban	New Tiedtke	Purdue Lin
4	NU_WDM6	bulk no urban	Grell 3D	WRF Doble Moment 6‐classes
5	NU_P3	bulk no urban	Grell 3D	P3
6	BULK_G3D	bulk	Grell 3D	Purdue Lin
7	BULK_GF	bulk	Grell−Freitas	Purdue Lin
8	BULK_NT	bulk	new Tiedtke	Purdue Lin
9	BULK_WDM6	bulk	Grell 3D	WRF Doble Moment 6‐classes
10	BULK_P3	bulk	Grell 3D	P3
11	SLUCM_G3D	SLUCM	Grell 3D	Purdue Lin
12	SLUCM_GF	SLUCM	Grell−Freitas	Purdue Lin
13	SLUCM_NT	SLUCM	new Tiedtke	Purdue Lin
14	SLUCM_WDM6	SLUCM	Grell 3D	WRF Doble Moment 6‐classes
15	SLUCM_P3	SLUCM	Grell 3D	P3

### 2.2 Methodology

First, results from the modeling should be validated using observational datasets. Then, the impact of the different ensembles is evaluated on the domain at seasonal scale. Later, the impact on the diurnal cycle of relevant variables both in the city centers and the vicinities is presented. The methodology used in each case is explained in the following subsections.

#### 2.2.1 Validation With Observations

To assess model performance in simulating relevant meteorological variables such as specific humidity (Q), as well as mean, maximum, and minimum values of temperature at 2 meters (T2), station data from ECAD are used for selected cities in the domain. The selected cities include Berlin, Budapest, Munich, Prague, Vienna, and Warsaw. All of the selected cities are large cities in Central Europe, and most of them with similar topography. They are inland cities with flat terrain, except for Budapest and Prague, which show a more complex orography. Model simulations are compared with observations by separating data from the city center and its surroundings, using mean monthly values of the relevant meteorological variables. For Prague, values from CHMI were also used due to the reduced availability of data in the ECAD database (i.e., only data from Klememtinum station is available and not for all analyzed variables). The specific stations used in the model evaluation are summarized in Table 2. Despite more stations being available in ECAD database for the urban and vicinity area of selected cities, only those with a complete series of data, that is, no missing data, for the simulated period are used.

**TABLE 2:** Stations used for specific variables.

	Stations city center		Stations vicinity	
City	T2 (mean, max, and min)	Q	T2 (mean, max, and min)	Q
Berlin	Mitte	Mitte	Buch Kaniswall Lichtenrade Lichterfelde‐Sud Rudow Schonefeld Spandau Zehlendorf	Buch Kaniswalll Lichtenrade Lichterfelde‐Sud Rudow Schonefeld Spandau Zehlendorf
Budapest	Allatkert Belterulet Lagymanyos	Allatkert Belterulet Lagymanyos	Janos‐Hegy 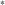 Ferihegy Pestszentlorinc Ujpest	Janos‐Hegy Ferihegy Pestszentlorinc Ujpest
Munich	Muenchen 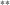 Bogenhausen	Muenchen Bogenhausen	Flughafen	Flughafen
Prague	Karlov 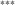	Karlov 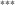	Kbely 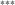 Libus 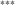 Ruzyne 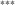	Kbely 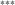 Libus 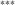 Ruzyne 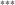
Vienna	Favoriten Innere Stadt	Favoriten Innere Stadt	Jubilaeumswarte Mariabrunn Mariabrunn II Stammersdorf Strebersdorf Unterlaa Unterlaa II	Jubilaeumswarte Mariabrunn Mariabrunn II Stammersdorf Strebersdorf Unterlaa Unterlaa II
Warsaw	Filtry Obserwatorium II	Obserwatorium II	Babice Bielany Okecie	Bielany Okecie

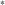
 Data used for the calculation of T2 mean only. 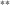 Data not available for T2 max. 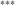 Data provided by the Czech Hydrometeorological Institute (CHMI).

To validate model outputs, multiyear monthly values of the aforementioned selected variables are calculated for every station shown in Table 2. Then, values for each city were obtained by averaging the values of the stations in the city center and the vicinity, respectively. As for the model outputs, the methodology followed is the same after extracting the values of each simulation at each of the chosen stations.

Last, a quantitative comparison between simulations and observations is performed for the same variables and the same stations but instead of average values, the whole 10‐year series is used. Once again, values from each simulations are extracted for each station shown in Table 2. The root mean square error (RMSE), normalized mean bias (NMB), and Pearson correlation coefficient (Corr) are the selected metrics. These results are listed in the Supplementary Section E.

#### 2.2.2 Evaluation Methodology

After validating model performance, the focus is on the impact that different combinations of urban, convection, and microphysics scheme have on relevant meteorological variables, with a special focus on precipitation and cloud cover. In the spatial comparisons, multiyear seasonal average values obtained with the NU simulations are taken as the control simulations, and differences between the bulk and NU, as well as between SLUCM and NU, are calculated. Then, a *t*‐test (Equation 1) has been carried out to identify the points where the calculated differences are statistically significant.

(1) $$t=\frac{\bar{x}-\mu_{0}}{s/\sqrt{n}},$$

where $\bar{x}$ represents the mean value of the difference between a simulation with urban scheme and the corresponding one with no urban scheme (i.e., with the same combinations of the other parameterizations); $\mu_{0}$ is the population mean, taken in this case as 0; s is the standard deviation of the difference; and n is the size of the sample. Taking into account the amount of data in the 10‐year simulation (large number of degrees of freedom) and considering a significance at the 99% level, the differences were masked for t values below 2.33. Please note that all grid points in the domain were used in this analysis, and no specific stations nor grid points were used.

For a more detailed evaluation, diurnal cycles comparing urban areas and their rural counterpart will be presented in the next section using all ensembles. To distinguish between urban and rural areas, the city center has to be identified first. The grid points of each geographical city center ($i_{c}$, $j_{c}$) are obtained using bilinear interpolation. After that, the four grid points separated one grid point from ($i_{c}$, $j_{c}$) are taken in each cardinal direction, that is, four points with coordinates ($i_{c}-1,j_{c}$), ($i_{c},j_{c}-1$), ($i_{c}+1,j_{c}$), and ($i_{c},j_{c}+1$). This process is described graphically in Figure SB.1. Then, the values of each variable in the corresponding city are calculated as an average of these four grid points to obtain a value for the city center. Values in the vicinity are calculated as an average of four boxes (of four grid points each), each centered over four grid points separated three grid points from the location of the city center. Thus, one value is obtained for the city center and another value for the vicinity. The location of the geographical city centers and the center of each of the boxes used in the calculations of the vicinity values are shown in Table S1.

## 3 Results

### 3.1 Model Evaluation

**FIGURE 2:**
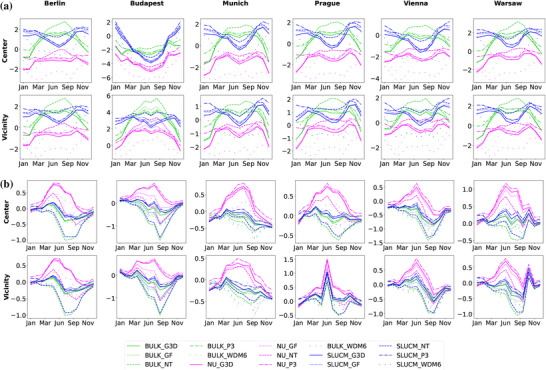
Comparison of multiyear mean monthly values of (a) T2 and (b) specific humidity for each city center and vicinity. Green colors show the values for the bulk urban scheme; magenta for the NO URBAN scheme; blue for the SLUCM scheme. Solid colored lines indicate the values for the simulations using the control setup; dotted lines for simulations with the Grell‐Freitas convection scheme, and dashed lines for the new Tiedtke convection scheme; dashed−dotted lines represent the values using the P3 microphysics scheme, and spaced−dotted lines represent the simulations with the WDM6 microphysics scheme. Values are shown in (a) °C, and (b) g/kg, respectively.

Comparisons between model outputs and observational data are conducted to assess model performance. As already mentioned in the previous section, the focus is on the mean, maximum, and minimum values of temperature at 2 m (T2) and the specific humidity. Multiyear monthly values of these variables are calculated for every station shown in Table 2. Then, values for each city were obtained by averaging the values of the stations in the city center and the vicinity, respectively. As for the model outputs, the method followed is the same after extracting the values of each simulation at each of the chosen stations. Model evaluation results for T2 are shown in Figure 2a, while results for the minimum and maximum values of T2 are included in Figures SC.1 and SC.2, respectively, for the sake of completeness. In general, simulations run with the bulk urban scheme tend to overestimate the mean temperature values, while those with no urban option tend to underestimate them. In general, simulations run with the SLUCM urban scheme show a better agreement with observations, which is expected considering that measurement stations are mainly located in urban areas. Therefore, the representation of the urban canopy affects the results. All simulations show similar patterns of maximum and minimum temperatures compared to observations, although the trend depends on the selected city and ensemble member. Comparing results between city center and vicinity, lower values are shown for the latter. Similar results were obtained for specific humidity in Figure 2b, although in this case, it is the no urban option that shows the strongest overestimation. The statistical evaluation of these results is included in Tables S2– S5 showing the values of RMSE, NMB, and Pearson correlation between each of the ensembles and each of the stations considered in Table 2 for mean T2, maximum and minimum T2, and specific humidity. The correlation coefficient is above 0.9 for the majority of the simulations, which also show low values of the RMSE (in general, less than 4) and NMB (lower than 0.2).

### 3.2 Impact of the Parameterizations on the Components of the Urban Meteorology Island

The UMI introduced by Karlický et al. [49] consists of several components, including temperature and specific humidity, among others. The impact that the choice of the urban, microphysics, and convection schemes have on some of these components, as well as a more detailed evaluation at a city level and diurnal temporal scales, is shown in Supplementary Sections F and G, respectively. In the following sections, only the impact on some others less documented components, that is, precipitation and cloud cover, will be analyzed and discussed. The first part of this analysis shows spatial differences between the different ensembles of bulk and SLUCM urban scheme with NU (the first subsection below), while the second subsection focuses on the impacts at city level and diurnal temporal scales.

#### 3.2.1 Spatial Differences

To evaluate the effect of the urban land‐surface impact and scheme selection in the domain, the “no urban” simulations are taken as a reference. Spatial differences over the domain between the simulations with two urban schemes and the no urban ones are analyzed here following the methodology described in the section of Evaluation Methodology. The urban‐induced changes in cloud fraction and hourly precipitation are evaluated in this section. Results for temperature and specific humidity are also shown here but as an average of all ensembles within one urban scheme. The purpose is to evaluate if these ensembles are within the range of other studies. The impact of each ensemble for these two variables can be found in the Supplementary Section F. Please note that the domain shown in all spatial figures is a zoomed‐in domain to focus only on the aforementioned selected cities, that is, Berlin, Budapest, Munich, Prague, Vienna, and Warsaw.

**FIGURE 3:**
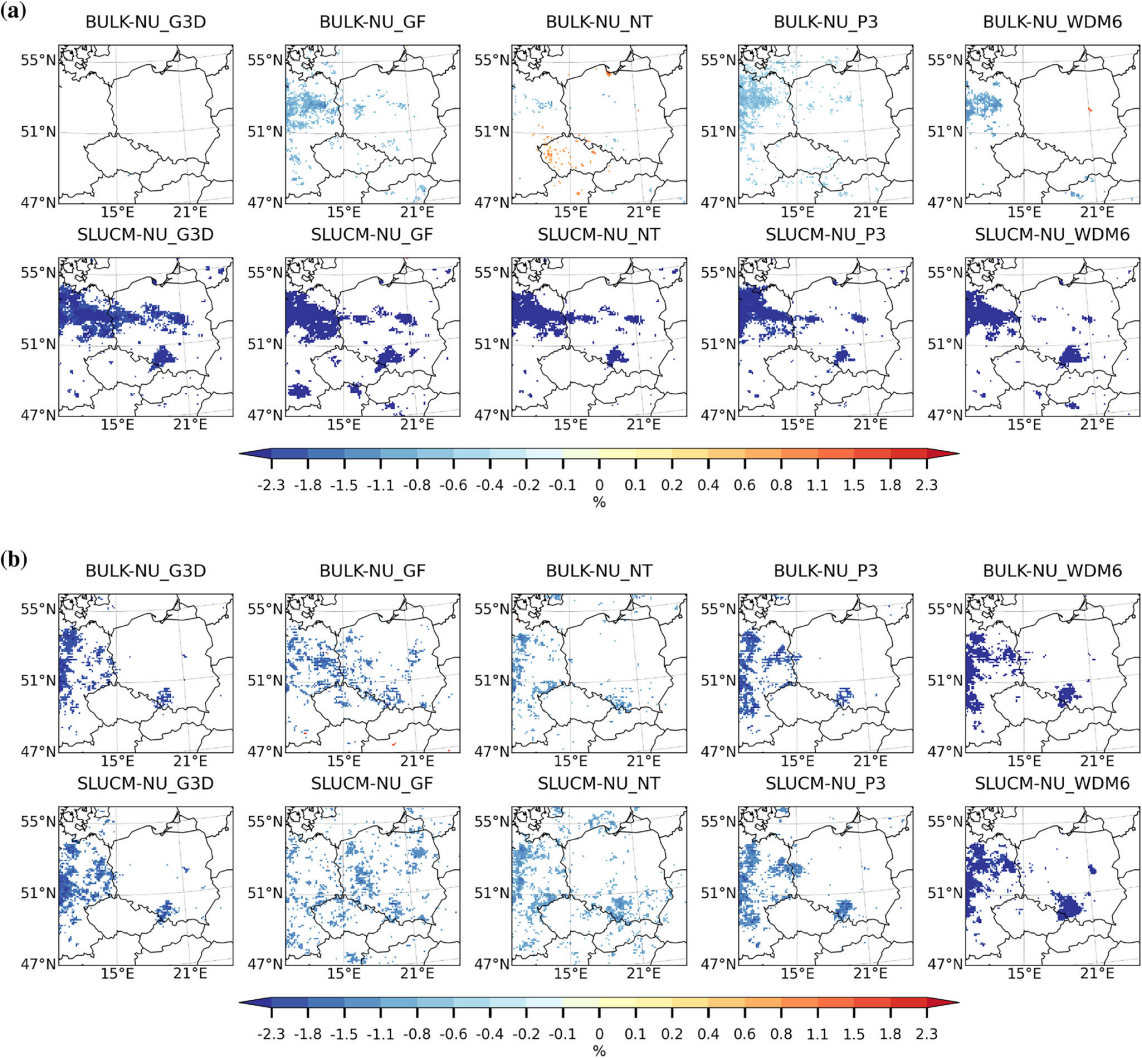
Spatial differences of multiyear seasonal average of cloud cover (%) between the BULK and no urban scheme (BULK−NU; upper row) simulations and between the SLUCM and no urban scheme (SLUCM−NU; lower row) simulations for (a) the winter season (DJF) and (b) the summer season (JJA) over a zoomed‐in domain. Results are only shown where there is a statistically significant difference at the 99% level using a *t*‐test.

In terms of cloud fraction, both urban schemes lead to a slight decrease over the urban areas in both studied seasons, as shown in Figures 3a and b, respectively. The influence of the urban scheme choice is more relevant in DJF than in JJA, when the choice of the convection and microphysics schemes seems to have stronger impacts. However, the impact that each of the analyzed combinations has on cloud fraction strongly depends also on the city of interest. For example, Prague shows the strongest decrease when bulk is used in combination with the GF convection scheme, while it does not show a statistically significant decrease in cloud cover when the SLUCM urban scheme and the G3D convection schemes are used.

**FIGURE 4:**
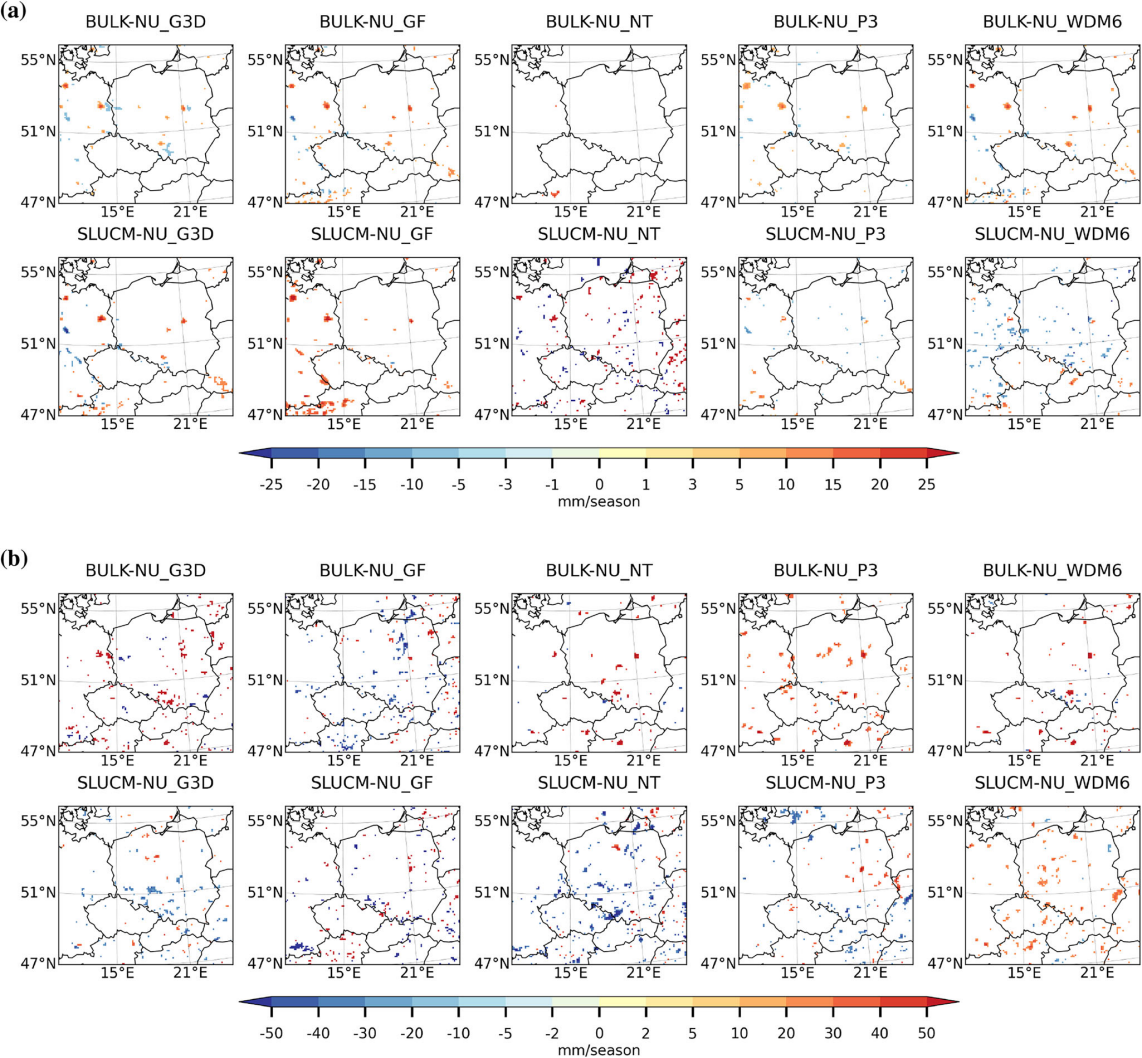
Spatial differences of multiyear seasonal average of precipitation (mm season^−1^) between the BULK and no urban scheme (BULK−NU; upper row) and between the SLUCM and no urban scheme (SLUCM−NU; lower row) simulations for (a) the winter season (DJF) and (b) the summer season (JJA) over a zoomed‐in domain. Results are only shown where there is a statistically significant difference at the 99% level using a *t*‐test.

The spatial distribution of seasonal sum precipitation, including large‐scale and subgrid‐scale precipitation, is shown in Figures 4a and b, respectively. In this case, the choice of the convection and microphysics parameterization seems to affect the distribution of precipitation more than the choice of the urban scheme, although in DJF, the urban scheme also plays a role. During DJF, precipitation shows a statistically significant increase over urban areas when the GF convection scheme is used, followed by the WDM6 microphysics scheme, although the increase is in general lower than 15‐mm season^−1^. In JJA, precipitation increases over cities more when the bulk scheme is used, especially in combination with the P3 and WDM6 microphysics scheme, reaching values between 40‐ and 50‐mm season^−1^. SLUCM also produces more precipitation over cities when these microphysics schemes are used.

**FIGURE 5:**
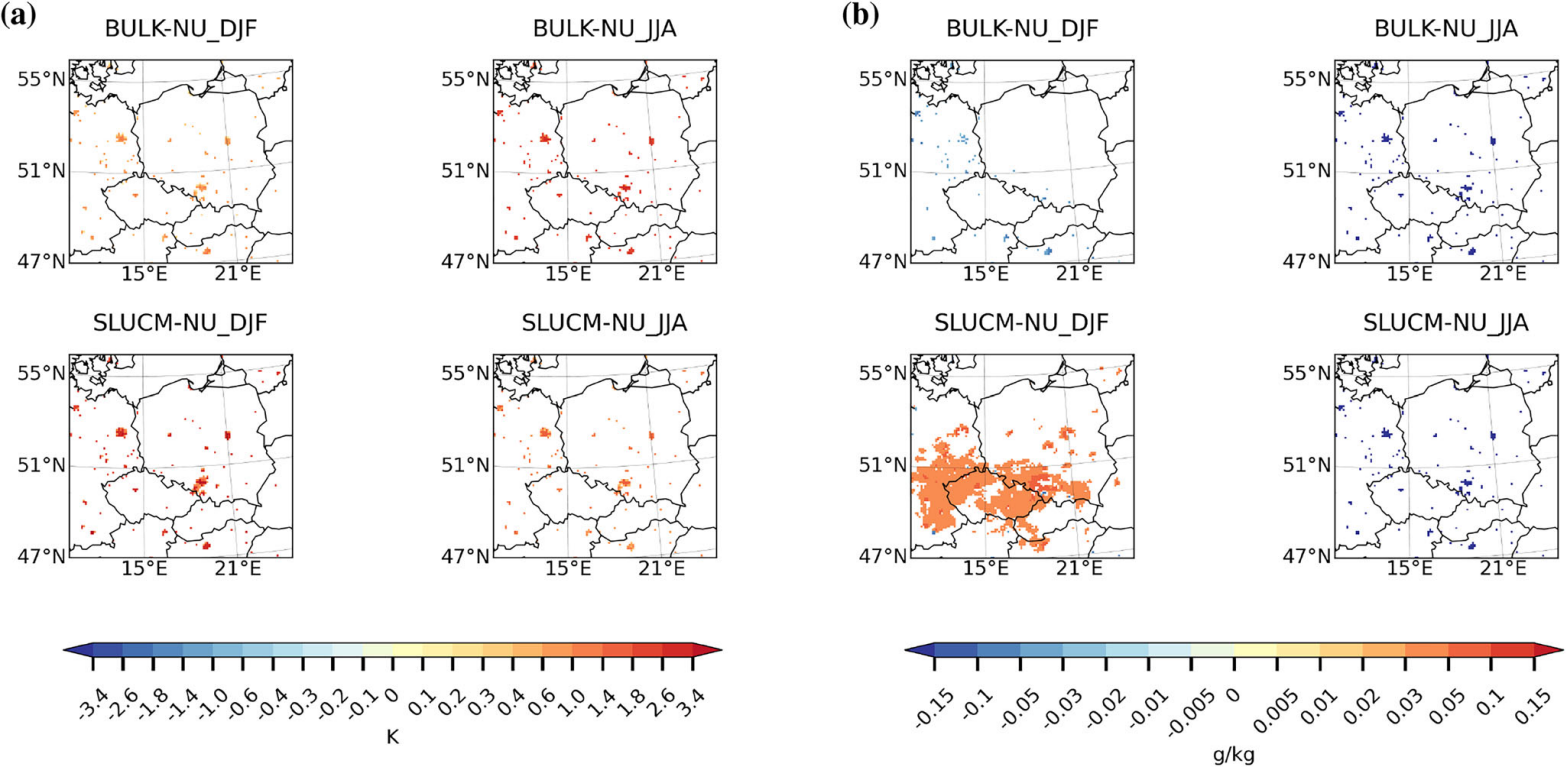
Spatial differences of multiyear seasonal average of (a) temperature at 2 m (°C) and (b) specific humidity (g/kg) between the BULK and no urban scheme (BULK−NU; upper row) average ensembles and between the SLUCM and no urban scheme (SLUCM−NU; lower row) average ensembles for the winter season (DJF, left column) and summer season (JJA, right column) over a zoomed‐in domain. Results are only shown where there is a statistically significant difference at the 99% level using a *t*‐test.

Lastly, to evaluate if our ensembles are within the range of other studies, the average impact of the different ensembles for bulk−NU and SLUCM−NU on temperature at 2 meters is shown in Figure 5a for the two seasons considered. Similarly, Figure 5b shows the impacts on specific humidity. Both variables are affected by the presence of cities. Differences in temperature are higher than 0.6 K over the evaluated cities than over their surroundings for both seasons, in agreement with previous results.

The specific humidity differences are also affected by the presence of urban areas, which tend to have lower values than over rural counterparts, which agrees with the results presented in previous studies. For both urban schemes, the reduction in specific humidity is stronger in JJA than in DJF. Besides, the impact of the selected microphysics and convection scheme is not relevant over cities during the evaluated seasons.

#### 3.2.2 Diurnal Values

This section presents a more detailed evaluation of the impact of urban areas on some relevant variables of the UMI. To do so, the focus is on the differences between the two urban schemes and the no urban simulation on the seasonally averaged diurnal cycle of meteorological variables over six selected cities in the domain, namely, Berlin, Budapest, Munich, Prague, Vienna, and Warsaw. For each city, the evaluation distinguishes between the city center and the vicinity following the methodology explained in the section of Evaluation Methodology. Thus, one value is obtained for the city center and another value for the vicinity. The location of the city centers and the center of each of the boxes used in the calculations of the vicinity values are shown in Table S1.

**FIGURE 6:**
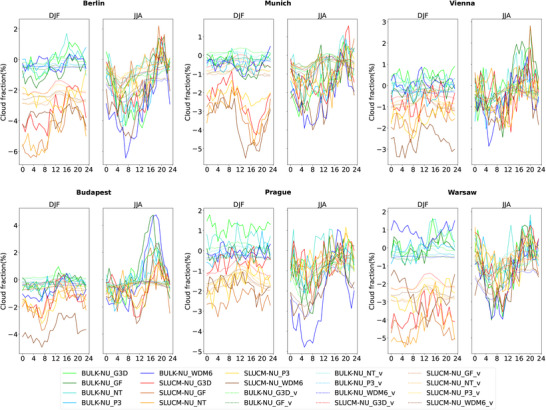
Diurnal cycle of city center (solid lines) and vicinity (dotted lines) for BULK−NU (green and blue lines) and SLUCM−NU (red and brown lines) for cloud cover (%). Differences between BULK−NU using the G3D convection scheme are shown in light green lines; BULK−NU with GF in dark green lines; BULK−NU with NT in turquoise lines; BULK−NU with the P3 microphysics scheme in light blue lines; and BULK−NU with WDM6 in dark blue lines. In case of SLUCM−NU differences, red lines show the differences when the G3D convection scheme is used; SLUCM−NU with GF are shown in light brown; SLUCM−NU with NT in orange; SLUCM−NU with P3 in yellow; and SLUCM−NU with WDM6 in dark brown. For each of the six selected cities, the panel on the left shows results for DJF and the panel on the right shows results for JJA.

The impact of urban areas on cloud cover is shown in Figure 6. In DJF, simulations with the bulk scheme produce higher values of cloud cover than the simulations with the SLUCM scheme. The daily evolution of cloud cover in this season is city‐dependent, showing a reduction during the evening and nighttime in the case of Vienna, while Budapest shows a reduction during nighttime and up to 10:00 am, and Munich reaches a minimum in cloud cover in the afternoon. In JJA, significant differences are found in the afternoon and evening for all selected cities, reaching the highest values at around 8:00 pm for the majority of the cities. The selection of the convection and microphysics schemes seems to play a major role here. The readers should keep in mind that the subgrid includes parameterized convection. The combination showing the highest differences depends on the chosen city.

In terms of the precipitation (including large‐scale and subgrid), the majority of the simulations show a slight increase during DJF for all cities, with some exceptions, such as the combination of SLUCM scheme with the WDM6 microphysics scheme in Warsaw, where precipitation slightly decreases. In JJA, precipitation increases in the afternoon and evening.

## 4 Discussion

Here, the results presented in the Results section will be discussed and compared with existing literature. The order in the discussion follows the one used in the previous section. In that sense, the discussion starts with the mean values of T2 for the different cities compared to ECAD data, followed by the spatial differences and the diurnal cycles for each city and its vicinity.

The mean values of T2 are better simulated with the SLUCM urban scheme in all selected cities and locations, except for Budapest, where the bulk urban scheme with NT microphysics simulates values of T2 closer to the observations. However, the bulk scheme tends to overestimate the mean value of temperature in agreement with the results presented in Salamanca et al. [82]. In the vicinity of Budapest, it is the combination of no urban with the WDM6 microphysics scheme closer to the observed data, despite not being the best combination as the urban areas are not present in the no urban scheme. For all urban schemes, the parameterization that leads to the highest values during the summer months is the NT convection scheme because of how the vertical mixing and moisture are handled in this scheme that uses values of entrainment proportional to the environmental relative humidity, which is more realistic than the fixed values used in the Grell3D scheme. This confirms the hypothesis presented in Karlický et al. [49], according to which summer temperatures could be influenced by the convection scheme used. The urban schemes, as first‐order effects, influence the local atmosphere just above the cities themselves, but higher‐order impacts are also expected as the air above the surroundings and over cities is linked via boundary layer dynamics. This means that any perturbation introduced at a particular point on the domain has at the end impact on the whole domain. The readers should keep in mind that all simulations used the Noah land use surface model, which uses a dominant land use approach. In the dominant land use, each grid cell has just one type of land use instead of fractions of different types. This means that the transition between urban and no urban is sharp from one grid cell to another, so the effects of the UMI and UHI will be stronger in the case of dominant land use compared to fractional land use. Last, simulations produce values of the specific humidity closer to observations with the SLUCM urban scheme combined with the G3D convection scheme or with the P3 microphysics scheme. Differences between the simulations and the observations are higher in the JJA months. The patterns observed in the city center are similar for the vicinities, and the scheme leading to the lowest values of specific humidity is the WDM6 microphysics scheme.

The impact of the UHI and UMI is visible around the big cities in the domain and for both seasons, no matter the combination of urban, convection, and microphysics schemes selected.

As shown in the *t*‐test evaluation in Figure 5a, the values of temperature at 2 meters are mostly affected by the selected urban scheme. In that sense, the SLUCM scheme leads to higher temperatures in DJF than the bulk scheme, both in the cities and in the vicinities, due to the anthropogenic heat production implementation in the former. The SLUCM urban scheme produces UHI intensities higher than 1.5 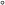 C, although the magnitude of these differences is city‐dependent. On the other hand, the bulk scheme produces higher values of temperature than the SLUCM scheme in JJA. This might be caused by the fact that the bulk scheme does not consider the three‐dimensional character of urban area, and the surface emissivity and surface albedo in the bulk scheme are lower than in the SLUCM scheme leading to higher temperatures in JJA when the bulk scheme is used [83]. Previous studies found similar results [18, 83, 84]. The selection of the urban scheme is much more relevant in the simulation of the UHI temperature than the convection and microphysics schemes [49].

In terms of the specific humidity, stronger decreases over urban areas are observed in JJA than in DJF, as shown in Figure 5b. While in DJF, the differences between the main big cities considered and their rural surrounding are around 0.05 g.kg^−1^, in JJA, the differences are much higher, in agreement with previous studies [85]. The urban scheme chosen is the main responsible for the values simulated in JJA, but in DJF, the values are also affected by the microphysics and convection scheme used (see Figures SF.3 and SF.4). Values of specific humidity follow a similar pattern as temperature. The SLUCM scheme produces higher values than the bulk scheme in DJF. Differences can be seen in the impact that the convection schemes other than GF, and microphysics schemes have on bulk and SLUCM during this season. Values of specific humidity simulated with the bulk option and the NT scheme are more similar to the NU than with any of the other two convection schemes. This may be explained by the different complexity of the convection schemes. GF and NT are more complex schemes than the Grell3D in terms of the type of convective modes simulate (three different convective modes, i.e., shallow, congestus, and deep in the GF scheme; shallow, deep, and midlevel convection in the new Tiedtke scheme; Grell3D only simulates deep convection), as well as in the closure assumption (CAPE‐based in Grell3D [74] and new Tiedtke scheme [86], and beta function in Grell‐Freitas [75]) and the values used for entrainment and detrainment (fixed in Grell3D, proportional to environmental relative humidity in new Tiedtke [87], and derived from the beta function in Grell‐Freitas). Higher values of entrainment rates can lead to lower specific humidity. In terms of the microphysics scheme used, P3 (multimoment) and WDM6 (double moment) are more complex than the default Purdue and Lin scheme (single moment), which, in general, translates to better representations of precipitation [88, 89] that in turn affect the values of specific humidity. In DJF, when the urban scheme used is the SLUCM, the simulated temperature is high in all possible combinations (as shown in Figure SF.1) for the reasons mentioned before when comparing values of temperature. The impact that these higher values of temperature have on specific humidity somehow mask the effect of more complex convection and microphysics schemes.

Values of the cloud cover also depend on the convection and microphysics schemes used more than on the urban scheme, especially in JJA (Figure 3a,b). Cloud cover differences between urban schemes are more pronounced in DJF than in JJA in line with the results obtained for temperature and specific humidity. Almost all possible combinations with the SLUCM urban scheme lead to a decrease in cloud cover in winter (DJF), while this decrease is not that strong when the bulk scheme is used. The cloud cover distribution shows a stronger dependence on the convection and microphysics scheme used in DJF. Regarding the effect of convection schemes, the spatial distribution of cloud cover shows a different pattern for each of the schemes. Differences among the results obtained with these schemes are probably caused by the differences in the parameterizations used concerning types of convective mode, closure, and entrainment rates, as described before when discussing the results obtained for specific humidity. The strongest reductions are obtained with the GF scheme, which uses a derived beta function both for the values of entrainment rates and for the closure of the scheme. Differences between G3D and NT are probably caused by the different values used for entrainment rates and the type of convective modes considered. In terms of the microphysics scheme used (here, we compare the results in the first column with results in the fourth and fifth column in Figure 3a), Purdue and Lin scheme produces higher cloud cover than any of the two microphysics schemes used. This can be caused by differences in the complexity of the parameterizations, and thus, in the treatment of the ice microphysics and fall velocity of hydrometeors. In fact, Purdue−Lin tends to produce too much graupel compared to observations and to other more complex microphysics schemes [90, 91], which may affect values of cloud cover, especially in winter. The representation of hydrometeors is more detailed in WDM6, but P3 is the only of these microphysics schemes that does not use fixed types of ice; instead, it predicts their properties dynamically. This can lead to a better representation of hydrometeors, in particular of ice, which also affects cloud cover in the coldest months. In warmer months (JJA), these differences in the representation of ice in the microphysics schemes do not play a major role. During this season, the most common type of clouds are liquid clouds instead of mixed‐phase clouds. WDM6 microphysics scheme is the one leading to the strongest decrease in cloud cover probably linked to a stronger production of precipitation. Figure 3b shows that both the convection and the microphysics schemes are the main drivers of cloud cover in JJA, and the type of urban scheme chosen does not play a major role.

The presence of urban areas leads to an enhancement in precipitation compared to their rural counterpart in the two analyzed seasons (Figure 4a,b), although the increase is much higher in JJA than in DJF and also city‐dependent. This tendency might be related to the enhancement of convection during summer (JJA), as well as to the presence of the urban canopy that could slow down the movement of precipitation clouds over these areas. The effect of urban drag on momentum transport was probed to strengthen low‐level convergence and moist convection, leading to heavy precipitation [92]. As in the case of the cloud cover, the selection of both convection and microphysics schemes plays an important role in the simulated precipitation. In DJF, it is the GF convection scheme leading to the highest values for both SLUCM and bulk urban schemes. In JJA, the values are also sensitive to the urban and the microphysics schemes. Among all possible combinations, the lowest values are obtained with the NT convection scheme in DJF. In JJA, GF leads to the lowest values for the bulk scheme, while for SLUCM, the lowest values are obtained with G3D and NT convection schemes. The microphysics scheme producing the lowest values of precipitation is the Purdue and Lin in almost all selected cities. Among the most detailed microphysics schemes, that is, P3 and WDM6, WDM6 scheme retrieves less precipitation in urban areas, which is in line with the results shown in Tewari et al. [93]. In general, more detailed microphysics schemes lead to results closer to observations than simpler ones [60].

**FIGURE 7:**
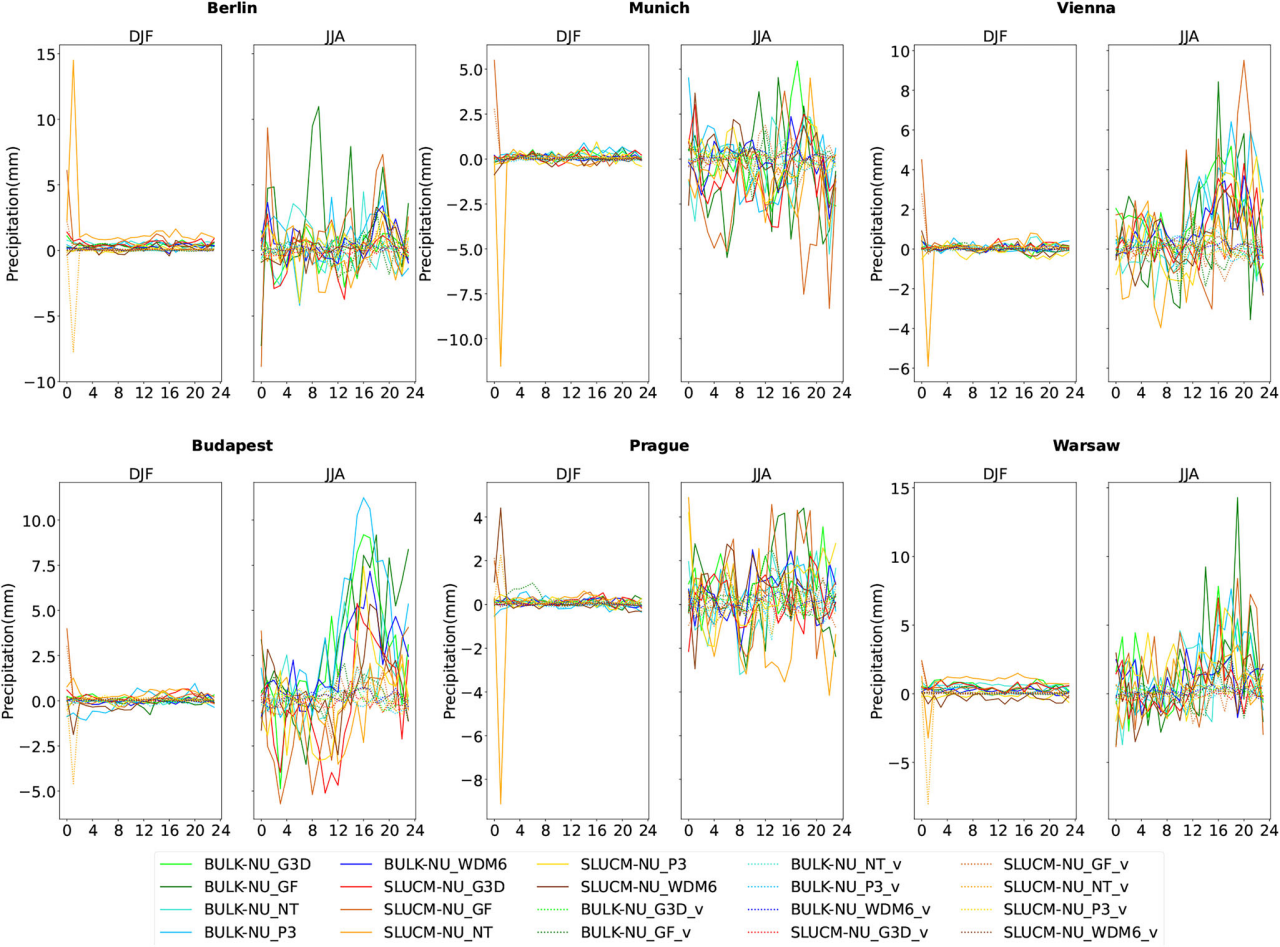
Diurnal cycle of city center (solid lines) and vicinity (dotted lines) for BULK−NU (green and blue lines) and SLUCM−NU (red and brown lines) for precipitation (mm). Differences between BULK−NU using the G3D convection scheme are shown in light green lines; BULK−NU with GF in dark green lines; BULK−NU with NT in turquoise lines; BULK−NU with the P3 microphysics scheme in light blue lines; and BULK−NU with WDM6 in dark blue lines. In case of SLUCM−NU differences, red lines show the differences when the G3D convection scheme is used; SLUCM−NU with GF are shown in light brown; SLUCM−NU with NT in orange; SLUCM−NU with P3 in yellow; and SLUCM−NU with WDM6 in dark brown. For each of the six selected cities, the panel on the left shows results for DJF and the panel on the right shows results for JJA.

The cloud cover is also affected by the presence of urban areas. Despite the differences between cities, simulations, and seasons, cloud cover increases over urban areas during the afternoon and evening and decreases during the morning and night, in agreement with the results presented by previous studies [39, 50]. In cities like London, cloud cover was strongly enhanced during low wind conditions and due to urban‐induced thermal effect that produces a strong moisture convergence over the city [94]. Vo et al. [43] also found an increase in nocturnal cloud cover over cities. However, the enhancement of cloud cover that the authors found in winter in the majority of US cities is not clear in our simulations, as it depends on the selected city and the climate the city belongs to. The increase in cloud cover in summer during the afternoon and evening matches the increase in precipitation shown in Figure 7. Therefore, this increase is probably a consequence of enhanced convection during this time of the day and of the year. On the other hand, the reduction of cloud cover during the morning hours in winter is not followed by a reduction in precipitation. A possible explanation for this behavior in cloud cover is a partial dissolution of fog and nonprecipitating clouds in winter and over cities due to higher temperatures in these areas. When comparing changes in precipitation in DJF and JJA, it is clearly seen that it is nosier for JJA, a pattern already identified by other researchers [49, 85] and caused by more convective precipitation during these months. Luo et al. [46] showed that urbanization enhanced precipitation over urban areas in the Greater Bay area in China and that this enhancement is higher when the SLUCM scheme is used compared to a no urban canopy model. However, the authors observed an increase in specific humidity that we did not observe in our simulations. Differences might be due to the coastal features of the cities in the Greater Bay area. Similar increases in precipitation were observed in other studies [47, 95].

Compared to studies using convection‐permitting (CP) models, similar results have been presented, although not so many studies were carried out for a long‐term period, especially those focused on the impact of urban areas on precipitation. CP studies also reported an increase in temperature over urban areas compared to their rural counterparts [96, 97, 98, 99, 100, 101]. Many of them showed an improved representation of the UHI compared to non‐CP simulations [98, 100], especially over areas with complex topography [102], because of a better representation of the orography when the resolution of the model is finer, but also due to a better representation of the radiative budget and clouds, among other reasons [102]. However, CP simulations do not always lead to better results. Recently, it was shown that CP over Johannesburg in South Africa produced an overestimation of the UHI that led to stronger values of precipitation than the ones observed. In terms of precipitation, it also increases over urban areas in CP models [52, 103], showing daily increases between 4% and 8% per degree warming, which are stronger in JJA than in DJF [101]. More recently, other studies reported other factors affecting precipitation over urban areas. For example, the presence of urban areas leads to updraft that favors moist convection with water vapor and cloud fraction increase over the urban area. This can cause an increase in precipitation downwind of the prevailing wind conditions [104]. Other studies reported that the increase in precipitation is stronger for compact development than for disperse [105], and the impact of urban areas on precipitation also depends on the synoptic forcing when aerosols are also considered in simulations [98]. The main advantage of CP simulations is that they can resolve convection explicitly and do not need to rely on the use of parameterizations that are based on assumptions and empirical values. This leads to a better representation of both precipitation and cloud cover in these simulations compared to those using parameterizations. The reader is referred to the Lucas‐Picher et al. [102] paper for a comprehensive review on the comparison of CP models and RCMs.

## 5 Conclusions

This study presented results from an ensemble of 15 simulations using the WRF model with different combinations of urban, convection, and microphysics schemes. Simulations covered a Central European domain with a 9‐km horizontal resolution for 10 years. Observational data was used to evaluate our simulations and spatial differences and diurnal cycles were calculated for winter and summer time. The main goal was to evaluate the impact that different urban canopy treatments, convection, and microphysics parameterizations have in the simulation of the UHI and, in particular, in those variables of the UMI that traditionally have received less attention, which include cloud fraction and precipitation.

The results presented here agree with those in Karlický et al. [49] and highlight the impact that urban areas have on relevant meteorological values compared to their rural counterparts. However, quantitative differences exist between the selected cities and combinations of parameterizations used.

The main findings of this manuscript are:

- Comparisons with observations from ECAD stations showed differences among cities and among the individual model situations caused by the different combinations of urban, convection, and microphysics schemes. In general, the SLUCM urban scheme agrees better with observations of mean temperature in the selected city centers, while the bulk scheme tends to overestimate them. In the vicinity, the no urban scheme with WDM6 microphysics scheme is the one producing results more similar to the observed values. Values of the specific humidity were closer to observations with the SLUCM urban scheme combined with the G3D convection scheme or with the P3 microphysics scheme.
- Focusing on spatial differences, a stronger signal in temperature is observed with the SLUCM urban scheme leading to T2 values higher than 1°C compared to the no urban scheme. The scheme playing the most important role in the simulation of the UHI is the urban scheme. Specific humidity over cities decreases more in JJA than in DJF with differences between the main big cities considered and their rural surroundings around 0.5 g.kg^−1^ in JJA. Despite the urban scheme driving the values of specific humidity in JJA, in DJF, the other two parameterizations also play a role.
- The cloud cover strongly depends on the convection and microphysics schemes, especially in JJA, while in DJF, the urban scheme chosen also plays a role. The impacts of the different parameterizations depend on the selected city.
- Precipitation is enhanced over urban areas, showing a more pronounced increase in JJA. The selection of both convection and microphysics schemes plays an important role in the simulated precipitation.
- The diurnal cycle of cloud cover is affected by the presence of urban areas increasing during the afternoons in JJA. The increase in cloud cover in JJA during the afternoon and evening matches the increase in precipitation. Therefore, this increase is probably a consequence of enhanced convection during this time of the day and of the year. The reduction of cloud cover during the morning hours in DJF is not followed by a reduction in precipitation, possibly because of a partial dissolution of fog and nonprecipitating clouds in DJF and over cities due to higher temperatures in these areas.
- Precipitation is also enhanced over cities during JJA afternoons caused by more convection during these months than during DJF.

This study highlighted the importance of using model ensembles and a number of cities when evaluating the UHI and UMI meteorological values, as large differences exist among the different setups and the selected cities when different combinations of schemes are used. Despite convection‐permitting simulations being more appropriate to resolve convection and avoid the used of schemes based on assumptions and empirical values, other processes such as microphysics still need to rely on parameterizations due to their subgrid‐ scale nature, and the impact of these choices depends on the studied city.

## Author Contributions

P.H. conceived the main scientific idea and organized the project team, in collaboration with T.H. J.K. prepared the input and evaluation data. M.Ž. prepared the data from CHMI. A.V.‐P. performed the model simulation. A.V.‐P. analyzed the resulting data, with contributions from J.K. and P.H. A.V.‐P. wrote the text, and P.H., J.K., M.Ž., and T.H. revised it.

## Conflicts of Interest

The authors declare no potential conflicts of interest.

## Supporting information

Data S1

Data S1
