# Supplementary material for: Long‐Term Impact of Urban Areas on Meteorological Conditions Over Central Europe
Source: Ann N Y Acad Sci. 2025 Sep 28;1553(1):461–76. doi: 10.1111/nyas.70069 (PMC12645272; doi:10.1111/nyas.70069)
Supplement: Supplementary file 1 — Data S1 [file NYAS-1553-461-s002.pdf]

SUPPLEMENT

A Land use categories

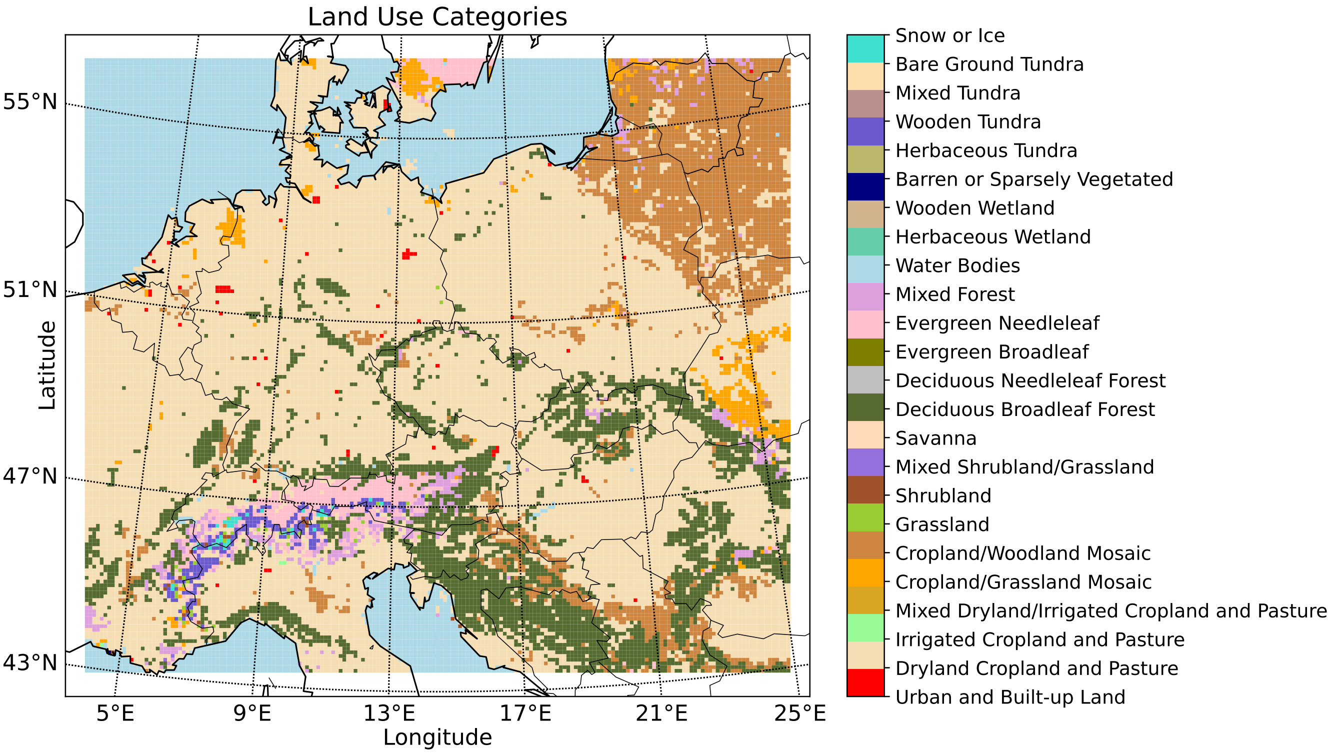

Figure A.1: Values of the CORINE land use used in WRF simulations for the different urban schemes except for the NO URBAN option, where the urban fraction is replaced by non-irrigated cropland and pastures.

B Calculation of urban vs rural values

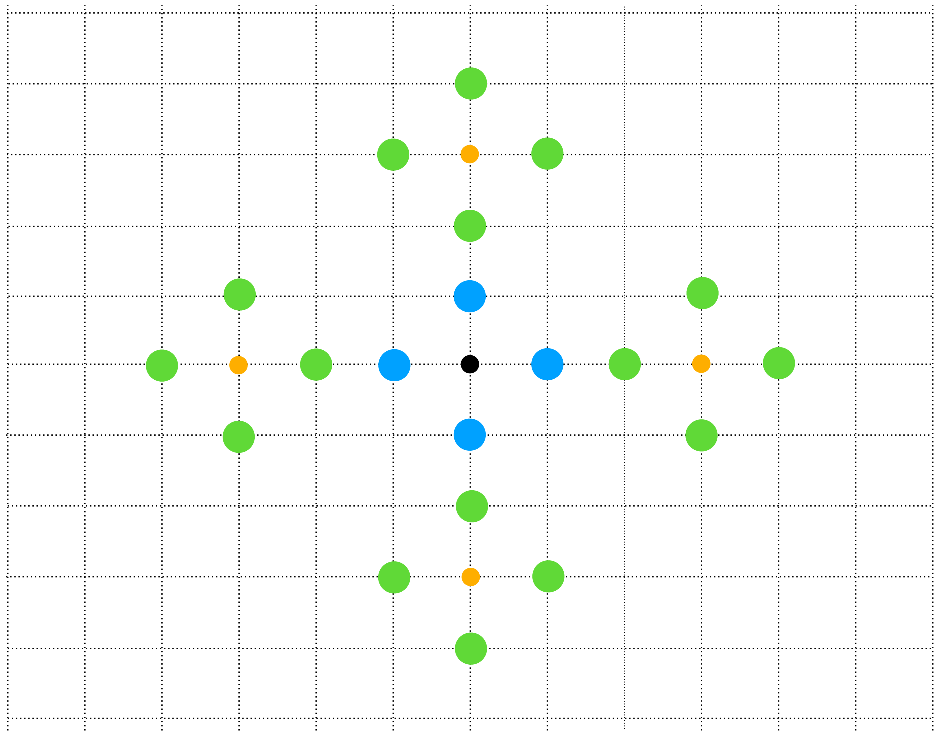

Figure B.1: Example of the calculation of the values for the city center (blue dots) and vicinity (green dots) for each of the selected cities. Orange dots mark the center of each vicinity box, while the black dot marks the location of the geographical city center in grid coordinates (i_c, j_c). As explained in Section 2.3, the grid points of each geographical city center (i_c, j_c) are obtained using bilinear interpolation. Then, four grid point separated one grid point from (i_c, j_c) are taken in each cardinal direction, i.e., four points with coordinates ($i_c - 1$,j_c), (i_c,$j_c - 1$), ($i_c + 1$,j_c) and (i_c,$j_c + 1$). Then, the values of each variable in the corresponding city are calculated as an average of these four grid points. Values in the vicinity are calculated as an average of four boxes (of four grid points each) each of them centered over four grid points separated three gridpoints from the location of the city center.

C Evaluation with observations for the minimum and maximum values of T2

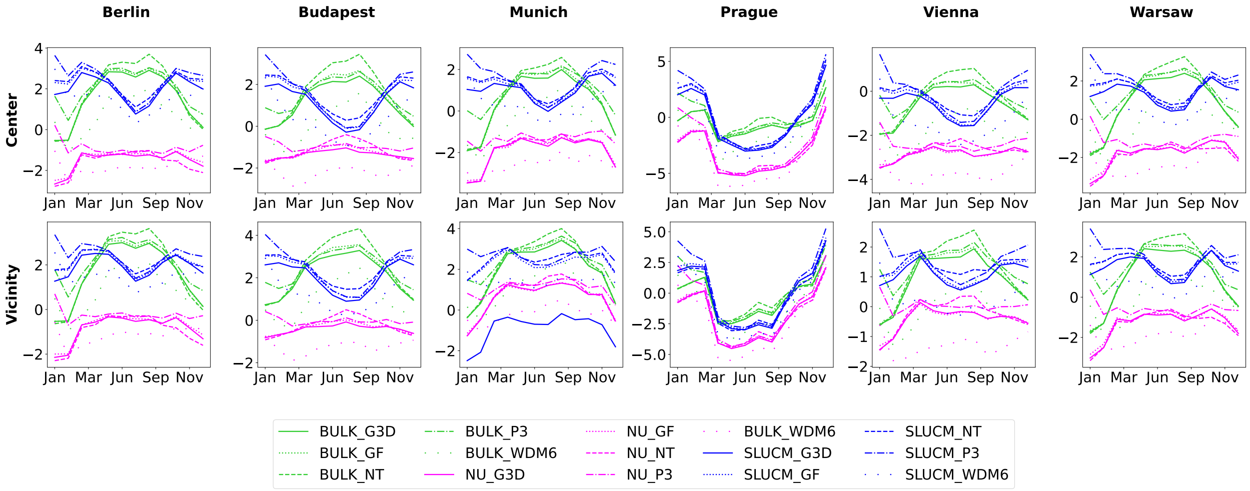

Figure C.1: Comparison of multi-year mean monthly values of minimum T2 for each city center and vicinity. Green colors show the values for the bulk urban scheme; magenta for the NO URBAN scheme; blue for the SLUCM scheme. Solid colored lines indicate the values for the simulations using the control setup; dotted lines for simulations with the Grell-Freitas convection scheme, and dashed lines for the new Tiedtke convection scheme; dashed-dotted lines represent the values using the P3 microphysics scheme, and spaced-dotted lines the simulations with the WDM6 microphysics scheme. Values are shown in °C.

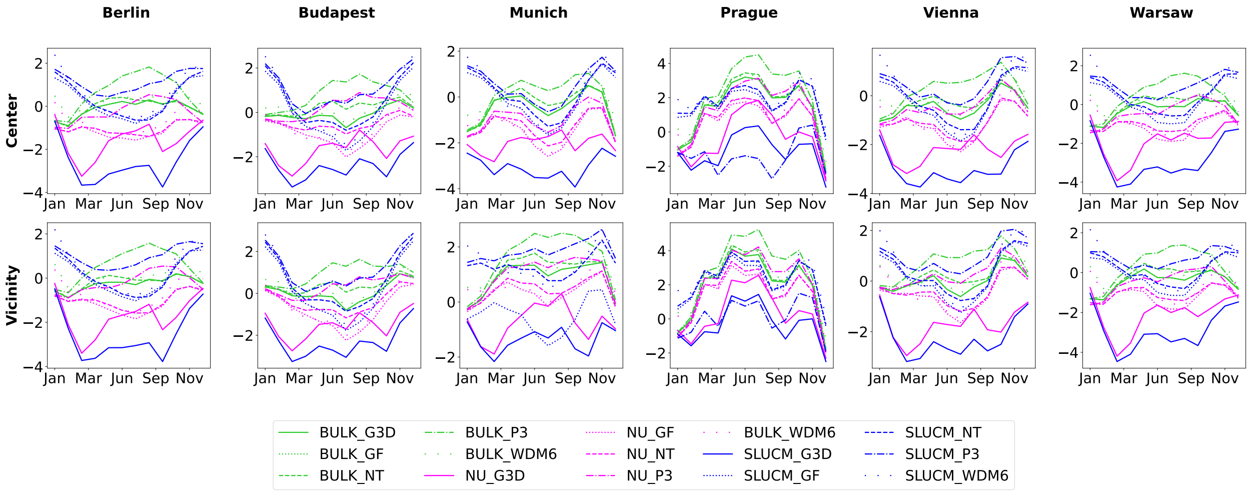

Figure C.2: Same as Fig. C.1 but for maximum T2 values.

In general, simulations tend to underestimate maximum temperature values except for the city of Prague where most ensemble members show an overestimation. For all analyzed cities, the highest values of maximum temperature are obtained with the bulk urban scheme in combination with the NT convection parameterization. In terms of the maximum T2, simulations tend to underestimate them. Generally, the best results are achieved with the bulk scheme combined with the GF convection scheme or with the SLUCM and the NT convection scheme in the city centers. The fact that the bulk scheme provides the best results for the maximum T2 is due to the fact that the bulk scheme often leads to stronger rise of daytime temperature towards their maxima due to not considering the morning shading effects of the urban built-up as it is in SLUCM (this scheme is closer to observations of mean temperatures despite underestimate them). Therefore, if there is a general underestimation of temperatures, bulk is usually closer to the observed maximum values. The combination leading to the lowest values of maximum T2 is that with the WDM6 microphysics scheme.

The bulk urban scheme tends to overestimate the minimum temperature values in all cities except in Prague between March and October. The SLUCM urban scheme retrieves the closest values of minimum temperature to observations for the majority of cities in the city center. In the vicinity, the no-urban scheme is the one that tends to produce better results compared to observations. The combination of any urban scheme with the WDM6 microphysics scheme leads to the lowest values of minimum T2. Comparing results between city center and vicinity, lower values are shown for the latter.

D Location of city centers and vicinities

Table 1: Location of the city centers and central point of the boxes used for the calculations in the city center and the surroundings.

**City**	**Center (lat,lon)**	**Center***	**Vicinity 1***	**Vicinity 2***	**Vicinity 3***	**Vicinity 4***
Berlin	52.52, 13.405	86,112	89,115	83,115	83,109	89,109
Budapest	47.49, 19.05	133,51	136,54	130,54	130,48	136,48
Munich	48.13, 11.58	70,59	73,62	67,62	67,56	73,56
Prague	50.09, 14.42	94,82	97,85	91,85	91,79	97,99
Vienna	48.21, 16.37	110,59	113,62	107,62	107,56	113,56
Warsaw	52.23, 21.01	144,11	147,114	141,114	141,108	147,108

* Values in grid coordinates.

E Statistical evaluation

This section presents the results of a quantitative analysis using three statistical metrics, i.e., root mean squared error (RMSE), normalized mean bias (NMB) and Pearson correlation coefficient (Corr) between each of the 15 ensemble members and observational data for the whole time period. The variables analyzed are temperature at 2 m (T2, Table 2), its maximum (T2max, Table 3) and minimum (T2min, Table 4) values, and specific humidity (Q2, Table 5). The abbreviations used in the tables are explained below.

**Abbreviation**	**Meaning**
BG3D	BULK_G3D
BGF	BULK_GF
BNT	BULK_NT
BP3	BULK_P3
BWDM6	BULK_WDM6
NG3D	NU_G3D
NGF	NU_GF
NNT	NU_NT
NP3	NU_P3
NWDM6	NU_WDM6
SG3D	SLUCM_G3D
SGF	SLUCM_GF
SNT	SLUCM_NT
SP3	SLUCM_P3
SWDM6	SLUCM_WDM6
BU	Buch
KA	Kaniswall
LR	Lichtenrade
LF	Lichterfelde-Sud
MI	Mitte
RU	Rudow
SC	Schonefeld
SP	Spandau
ZE	Zehlendorf
AL	Allakert
BE	Belterulet
JH	Janos-Hegy
FE	Ferihegy
LA	Lagymanyos
PE	Pestszentlorinc
UJ	Ujpest
BO	Bogenhausen
FL	Flughafen
MU	Muenchen
KR	Karlov
KB	Kbely
LI	Libus
RZ	Ruzyne
FA	Favoriten
IS	Inner-Stadt
JU	Jubilaeumswarte
MB	Mariabrunn
MBII	Mariabrunn II
ST	Stammersdorf
UN	Unterlaa
UNII	Unterlaa II
BA	Babice
BI	Bielany
FI	Filtry
OB	Obserwatorium II

Table 2: Statistical analysis showing root mean square error (RMSE), normalized mean bias (NMB) and Pearson correlation (Cor) between each simulation and each of the stations considered for T2. The abbreviations used in the Table are explained in the introduction of this section.

Sims	Stat	BU	KA	LR	LF	MI	RU	SC	SP	ZE	AL	BE	JH	FE	LA	PE	UJ	BO	FL	MU	KR	KB	LI	RZ	FA	IS	JU	MB	MBII	ST	UN	UNII	BA	BI	FI	OB	OK
	RMSE	1.708	37.59	1.922	1.575	1.465	1.638	1.758	1.758	1.857	2.143	1.455	3.141	2.075	1.645	1.952	2.314	1.723	1.654	1.756	1.704	2.775	1.687	2.012	1.687	1.687	2.010	2.069	2.069	1.831	1.833	1.613	1.613	1.895	1.772	2.170	1.805
BG3D	NMB	-0.0634	-0.207	-0.1052	-0.0425	-0.060	-0.0888	-0.0888	-0.0394	-0.0996	-0.1248	-0.0259	-0.2877	-0.1352	-0.0719	-0.1175	-0.1493	-0.0175	0.0611	-0.0107	-0.0212	-0.0067	-0.0363	-0.1246	0.0464	0.0464	-0.1236	-0.0489	-0.0489	-0.0488	-0.0451	0.0195	0.0195	-0.0674	-0.0537	-0.1220	-0.0689
	Corr	0.986	0.172	0.987	0.987	0.988	0.987	0.988	0.988	0.987	0.986	0.989	0.988	0.989	0.989	0.989	0.987	0.983	0.982	0.982	0.984	0.9420	0.983	0.983	0.984	0.984	0.984	0.975	0.975	0.981	0.980	0.982	0.982	0.985	0.988	0.987	0.988
BGF	RMSE	1.749	37.598	1.978	1.631	1.694	1.806	1.806	1.511	1.913	2.375	1.592	3.318	2.290	1.845	2.163	2.551	1.776	1.645	1.814	1.734	2.773	1.716	2.066	1.660	1.660	2.083	2.131	2.131	1.888	1.888	1.593	1.593	1.970	1.843	2.283	1.901
	NMB	-0.0690	-0.2125	-0.1103	-0.0474	-0.0667	-0.0942	-0.0941	-0.0456	-0.1053	-0.1446	-0.0440	-0.3071	-0.1567	-0.0907	-0.1386	-0.1695	-0.0279	0.0511	-0.223	-0.0274	-0.0137	-0.0430	-0.1319	0.0379	0.0379	-0.1337	-0.0593	-0.0593	-0.0583	-0.0546	0.0116	0.0116	-0.0799	-0.0656	-0.1351	-0.0818
	Corr	0.987	0.173	0.987	0.987	0.988	0.987	0.987	0.988	0.987	0.987	0.990	0.989	0.990	0.990	0.990	0.988	0.983	0.983	0.982	0.984	0.943	0.984	0.984	0.985	0.985	0.985	0.975	0.975	0.981	0.981	0.983	0.983	0.986	0.988	0.987	0.989
BNT	RMSE	2.152	37.634	2.463	2.011	2.110	2.256	2.255	1.878	2.397	2.789	1.979	3.877	2.751	1.75	2.619	2.999	1.984	1.657	1.992	2.030	2.898	2.025	2.484	1.723	1.723	2.461	2.403	2.403	2.181	2.174	1.716	1.716	2.283	2.163	2.659	2.224
	NMB	-0.1116	-0.2641	-0.1559	-0.0904	-0.1087	-0.1374	-0.1374	-0.0862	-0.1497	-0.1801	-0.0801	-0.3617	-0.1929	-0.1260	-0.1743	-0.2058	-0.0594	0.0284	-0.0488	-0.0684	-0.0475	-0.0840	-0.1792	0.0047	0.0047	-0.1765	-0.0972	-0.0972	-0.0939	-0.0901	-0.0243	-0.0243	-0.1180	-0.1028	-0.1742	-0.1183
	Corr	0.987	0.173	0.987	0.988	0.988	0.987	0.987	0.988	0.987	0.987	0.989	0.989	0.989	0.989	0.989	0.988	0.983	0.983	0.982	0.983	0.943	0.983	0.983	0.984	0.984	0.984	0.975	0.975	0.981	0.980	0.982	0.982	0.986	0.989	0.988	0.989
BP3	RMSE	1.670	37.598	1.952	1.487	1.560	1.777	1.777	1.390	1.890	2.304	1.463	3.318	2.255	1.757	2.117	2.480	1.563	1.295	1.579	1.558	2.742	1.583	2.112	1.506	1.506	2.154	2.050	2.050	1.850	1.845	1.543	1.543	1.858	1.676	2.198	1.764
	NMB	-0.1011	-0.2581	-0.1430	-0.0782	-0.0955	-0.1277	-0.1278	-0.0747	-0.1372	-0.1475	-0.0469	-0.3099	-0.1583	-0.0929	-0.1407	-0.1725	-0.0568	0.0104	-0.0502	-0.0612	-0.0507	-0.0810	-0.1704	0.0226	0.0226	-0.1555	-0.0782	-0.0782	-0.0786	-0.0748	-0.0041	-0.0041	-0.1178	-0.1019	-0.1733	-0.116
	Corr	0.989	0.173	0.990	0.990	0.991	0.990	0.990	0.991	0.990	.987	0.989	0.989	0.990	0.990	0.989	0.988	0.986	0.986	0.985	0.987	0.943	0.987	0.986	0.985	0.985	0.985	0.977	0.977	0.983	0.982	0.983	0.983	0.988	0.991	0.990	0.991
BWDM6	RMSE	2.099	37.586	2.072	2.156	2.126	2.025	2.024	2.087	2.041	2.007	2.233	2.294	1.894	1.994	1.892	2.045	2.260	2.437	2.270	2.356	3.153	2.186	2.025	2.976	2.976	2.028	2.602	2.602	2.424	2.453	2.548	2.548	2.401	2.397	2.340	2.273
	NMB	0.0598	-0.0705	0.0265	0.0817	0.0637	0.0410	0.0410	0.0792	0.0306	-0.0073	0.0861	-0.1468	-0.0168	0.0404	-0.0010	-0.0292	0.1175	0.1857	0.1193	0.1124	0.1096	0.1033	0.0295	0.1670	0.1670	0.0249	0.0901	0.0901	0.0858	0.0890	0.1381	0.1381	0.0910	0.1011	0.0429	0.0896
	Corr	0.977	0.169	0.990	0.973	0.973	0.973	0.973	0.973	0.972	0.978	0.980	0.982	0.980	0.98	0.980	0.978	0.977	0.977	0.977	0.972	0.932	0.975	0.972	0.973	0.973	0.974	0.963	0.963	0.969	0.968	0.972	0.972	0.972	0.974	0.972	0.975
NG3D	RMSE	1.719	37.569	1.535	1.883	1.921	1.543	1.543	1.907	1.575	1.411	1.814	2.008	1.341	1.504	1.413	1.305	2.188	1.723	2.234	2.232	2.729	1.910	1.519	2.601	2.601	1.567	1.895	1.895	1.757	1.793	1.701	1.701	1.831	1.962	1.666	1.638
	NMB	0.1101	-0.1626	0.0719	0.1245	0.126	0.0786	0.0786	0.1284	0.0781	0.0249	0.0981	-0.1475	0.0202	0.06239	0.0355	0.0037	0.1514	0.0758	0.1588	0.1454	0.0249	0.1116	0.0286	0.1614	0.1614	-0.0125	0.0551	0.0551	0.0652	0.0684	0.0482	0.0482	0.0986	0.1259	0.0693	0.0763
	Corr	0.987	0.173	0.986	0.986	0.985	0.987	0.986	0.985	0.985	0.988	0.988	0.987	0.989	0.989	0.988	0.989	0.982	0.982	0.982	0.981	0.944	0.982	0.984	0.983	0.983	0.983	0.976	0.976	0.982	0.981	0.981	0.981	0.985	0.986	0.987	0.988
NGF	RMSE	1.66	37.578	1.494	1.829	1.851	1.503	1.503	1.841	1.522	1.410	1.625	2.137	1.280	1.400	1.328	1.361	2.104	1.676	2.141	2.161	2.716	1.841	1.490	2.537	2.537	1.534	1.859	1.859	1.0708	1.743	1.662	1.662	1.749	1.860	1.615	1.575
	NMB	0.1030	-0.1730	0.0643	0.1173	0.1182	0.07010	0.0709	0.1211	0.0697	0.0041	0.0777	-0.171	-0.0020	0.0425	0.01357	-0.0176	0.1407	0.0637	0.1482	0.1381	0.0155	0.1035	0.0218	0.1569	0.1569	-0.0203	0.0474	0.0474	0.0594	0.0627	0.0437	0.0437	0.0855	0.1122	0.0547	0.0633
	Corr	0.987	0.1728	0.987	0.986	0.986	0.987	0.986	0.985	0.986	0.988	0.989	0.989	0.990	0.990	0.989	0.989	0.983	0.983	0.983	0.982	0.945	0.983	0.984	0.984	0.984	0.984	0.977	0.977	0.983	0.982	0.982	0.982	0.986	0.987	0.987	0.988
NNT	RMSE	1.674	37.607	1.574	1.734	1.795	1.520	1.519	1.751	1.588	.558	1.502	2.618	1.449	1.452	1.447	1.611	2.044	1.698	2.078	2.040	2.804	1.781	1.607	2.288	2.288	1.651	1.872	1.872	1.680	1.709	1.658	1.658	1.744	1.820	1.715	1.636
	NMB	0.073	-0.2177	0.0340	0.0888	0.0917	0.0414	0.0413	0.0945	0.0402	-0.0285	0.0425	-0.2258	-0.0369	0.0098	-0.0208	-0.0509	0.1238	0.0454	0.1318	0.1098	-0.0137	0.0748	-0.0169	0.1278	0.1278	-0.0589	0.0138	0.0138	0.0272	0.0306	0.0098	0.0098	0.06288	0.0888	0.0297	0.0401
	Corr	0.9870	0.1735	0.987	0.987	0.986	0.988	0.987	0.986	0.986	0.988	0.989	0.988	0.989	0.990	0.989	0.989	0.98	0.982	0.982	0.982	0.945	0.983	0.984	0.984	0.984	0.984	0.976	0.976	0.982	0.982	0.982	0.982	0.987	0.987	0.988	0.989
NP3	RMSE	1.242	37.579	.098	1.390	1.420	1.112	1.112	1.433	1.116	1.384	1.631	2.188	1.339	1.371	1.365	1.310	1.727	1.304	1.782	1.724	2.661	1.434	1.316	2.323	2.323	1.606	1.735	1.735	1.565	1.597	1.583	1.583	1.391	1.428	1.206	1.192
	NMB	0.0645	-0.219	0.0245	0.0798	0.0808	0.0308	0.0307	0.0839	0.0304	-0.0008	0.0752	-0.1697	-0.0071	0.0386	0.0086	-0.0226	0.1060	0.0212	0.1137	0.0967	-0.0249	0.0589	-0.0240	0.1364	0.1364	-0.0449	0.0249	0.0249	0.0346	0.0380	0.0228	0.0228	0.0406	0.0688	0.0084	0.01837
	Corr	0.990	0.173	0.990	0.990	0.989	0.991	0.991	0.989	0.990	0.987	0.989	0.987	0.989	0.989	0.988	0.989	0.985	0.986	0.985	0.985	0.945	0.987	0.987	0.984	0.984	0.983	0.978	0.978	0.983	0.982	0.982	0.982	0.988	0.990	0.990	0.991
NWDM6	RMSE	2.734	37.514	2.547	3.003	3.0146	2.595	2.595	2.995	2.582	2.299	3.042	1.699	2.209	2.632	2.345	2.112	3.203	2.562	3.256	3.293	3.239	2.907	2.334	3.965	3.965	2.331	2.946	2.946	2.915	2.956	2.777	2.777	2.990	3.174	2.715	2.774
	NMB	0.2123	-0.0259	0.1783	0.2249	0.2243	0.1844	0.1844	0.2246	0.1821	0.1245	0.1965	-0.0204	0.1204	0.1581	0.1341	0.1055	0.2654	0.2034	0.2718	0.2553	0.1430	0.2284	0.1621	0.2688	0.2688	0.1263	0.1833	0.1833	0.1873	0.1902	0.1671	0.1671	0.2312	0.2533	0.2049	0.2123
	Corr	0.975	0.1765	0.972	0.972	0.970	0.973	0.972	0.971	0.971	0.980	0.980	0.981	0.981	0.981	0.981	0.981	0.977	0.976	0.977	0.973	0.933	0.975	0.975	0.974	0.974	0.973	0.965	0.965	0.972	0.971	0.972	0.972	0.973	0.974	0.974	0.975
SG3D	RMSE	1.932	37.584	1.976	1.584	1.813	1.884	1.884	1.649	1.938	2.565	1.798	3.547	2.451	2.104	2.314	2.695	1.764	1.567	1.808	1.832	2.808	1.696	2.194	1.840	1.840	2.390	2.251	2.251	2.026	2.035	1.615	1.615	2.057	1.935	2.312	1.832
	NMB	-0.1178	-0.225	-0.1496	5-0.084	-0.1089	-0.1317	-0.1316	-0.1439	-0.1438	-0.1381	-0.0417	-0.3207	-0.1455	-0.0839	-0.1276	-0.1629	-0.0761	0.0506	-0.666	-0.0594	-0.0216	-0.069	-0.1653	0.0324	0.0324	-0.1592	-0.0739	-0.0739	-0.0654	-0.0617	0.0088	0.0088	-0.1274	-0.1133	-0.1854	-0.1247
	Corr	0.980	0.174	0.985	0.986	0.984	0.986	0.985	0.984	0.985	0.977	0.983	0.988	0.984	0.981	0.984	0.977	0.980	0.983	0.977	0.978	0.939	0.982	0.980	.979	0.979	0.980	0.966	0.966	0.974	0.974	0.981	0.981	0.983	0.984	0.983	0.987
SGF	RMSE	1.971	37.595	2.022	1.615	1.842	1.9380	1.937	1.663	1.979	2.707	1.793	3.655	2.565	2.167	2.421	2.849	1.779	1.544	1.818	1.792	2.794	1.675	2.236	1.738	1.738	2.409	2.274	2.274	2.036	2.040	1.576	1.576	2.090	1.955	2.394	1.876
	NMB	-0.1321	-0.2311	-0.1636	-0.0977	-0.1237	-0.1450	-0.1449	-0.0949	-0.1579	-0.1643	-0.0662	-0.3401	-0.1725	-0.1089	-0.1543	-0.1896	-0.0956	0.0392	-0.0869	-0.0747	-0.0301	-0.0846	-0.1816	0.0197	0.0197	-0.1677	-0.0889	-0.0889	-0.0794	-0.0756	0.0012	0.0012	-0.1480	-0.1345	-0.2080	-0.1449
	Corr	0.982	0.173	0.988	0.988	0.987	0.987	0.987	0.986	0.987	0.979	0.985	0.989	0.987	0.984	0.986	0.980	0.983	0.983	0.980	0.981	0.941	0.0.985	0.983	0.981	0.981	0.981	0.967	0.967	0.976	0.976	0.982	0.982	0.985	0.987	0.985	0.989
SNT	RMSE	2.253	37.635	2.373	1.860	2.117	2.228	2.228	1.883	2.321	2.935	1.949	4.057	2.829	2.367	2.674	3.114	1.902	1.554	1.925	1.946	2.916	1.856	2.527	1.664	1.664	2.681	2.462	2.462	2.208	2.204	1.675	1.675	2.291	2.154	2.656	2.0956
	NMB	-0.1735	-0.2833	-0.2068	-0.1384	-0.1647	-0.1865	-0.1864	-0.2010	-0.1338	-0.1971	-0.0986	-0.3897	-0.2055	-0.1403	-0.1867	-0.2231	-0.1224	0.0169	-0.1106	-0.1120	-0.0653	-0.1226	-0.2263	-0.0120	-0.0120	-0.2009	-0.1256	-0.1256	-0.1140	-0.1101	-0.0364	-0.0364	-0.1841	-0.1699	-0.2457	-0.1807
	Corr	0.982	0.173	0.988	0.989	0.988	0.988	0.987	0.986	0.987	0.980	0.986	0.989	0.987	0.985	0.986	0.981	0.984	0.983	0.980	0.982	0.941	0.985	0.983	0.981	0.981	0.981	0.968	0.968	0.977	0.976	0.982	0.982	0.986	0.988	0.986	0.990
SP3	RMSE	2.131	37.607	2.218	1.793	2.016	2.133	2.132	1.831	2.175	2.808	1.933	3.732	2.682	2.307	2.542	2.937	1.980	1.286	2.013	1.989	2.823	1.875	2.468	1.827	1.827	2.624	2.386	2.386	2.182	2.180	1.614	1.614	2.277	2.140	2.565	2.069
	NMB	-0.1522	-0.2735	-0.1851	-0.1179	-0.1413	-0.1680	-0.1680	-0.1138	-0.1792	-0.1645	-0.0655	-0.3395	-0.1717	-0.1082	-0.1534	-0.1898	-0.1125	0.0018	-0.1043	-0.0974	-0.0658	-0.1111	-0.2103	0.0053	0.0053	-0.1880	-0.1069	-0.1069	-0.0980	-0.0942	-0.0160	-0.0160	-0.1693	-0.1537	-0.2284	-0.1678
	Corr	0.981	0.1722	0.987	0.988	0.986	0.988	0.987	0.985	0.987	0.977	0.984	0.987	0.985	0.982	0.985	0.979	0.981	0.986	0.978	0.980	0.940	0.985	0.983	0.979	0.979	0.980	0.967	0.967	0.976	0.975	0.982	0.982	0.985	0.987	0.985	0.989
SWDM6	RMSE	1.952	37.542	1.845	1.791	1.861	1.863	1.862	1.844	1.832	2.206	1.967	2.677	2.051	1.988	1.988	2.256	1.866	2.288	1.906	2.042	3.102	1.856	1.935	2.616	2.616	2.074	2.408	2.408	2.232	2.261	2.328	2.328	2.154	2.080	2.155	1.930
	NMB	-0.0305	0.1012	-0.0557	0.0042	-0.0232	-0.0382	-0.0381	0.0042	-0.0505	-0.0529	0.0401	-0.2055	-0.0554	-0.0018	-0.0389	-0.0758	0.0209	0.1676	0.0263	0.0418	0.0859	0.0395	-0.0450	0.1266	0.1266	-0.0346	0.0355	0.0355	0.0421	0.0455	0.1147	0.1147	-0.0129	-0.0037	-0.0688	-0.0074
	Corr	0.968	0.174	0.973	0.974	0.973	0.973	0.973	0.972	0.973	0.972	0.977	0.981	0.978	0.976	0.978	0.972	0.972	0.977	0.971	0.971	0.930	0.976	0.972	0.971	0.971	0.972	0.956	0.956	0.965	0.965	0.974	0.974	0.971	0.974	0.971	0.976

Table 3: Statistical analysis showing root mean square error (RMSE), normalized mean bias (NMB) and Pearson correlation (Cor) between each simulation and each of the stations considered for T2max. The abbreviations used in the Table are explained in the introduction of this section.

Sims	Stat	BU	KA	LR	LF	MI	RU	SC	SP	ZE	AL	BE	FE	LA	PE	UJ	BO	FL	KR	LI	RZ	FA	IS	JU	MB	MBII	ST	SR	UN	UNII	BA	FI	OB	OK
	RMSE	1.735	2.122	1.643	1.666	1.595	1.612	1.612	1.581	1.648	2.112	2.141	2.035	2.023	2.009	2.147	2.181	2.168	2.005	1.953	2.096	2.173	2.173	2.582	2.262	2.262	2.161	2.168	2.207	2.207	2.034	1.960	1.847	1.853
BG3D	NMB	0.0399	0.0690	0.0157	-0.0039	0.0077	-0.0022	-0.0022	0.0152	-0.0016	0.0054	0.051	-0.0311	-0.0438	-0.0212	0.0086	0.0314	0.0443	0.0053	-0.0061	-0.0640	0.0269	0.0269	-0.1023	0.0364	0.0364	-0.0058	-0.0015	0.0088	0.0088	0.0316	0.0421	-0.0079	0.0187
	Corr	0.984	0.982	0.984	0.983	0.985	0.985	0.985	0.985	0.984	0.979	0.981	0.981	0.981	0.981	0.979	0.973	0.974	0.977	0.979	0.979	0.976	0.976	0.974	0.974	0.974	0.975	0.975	0.974	0.974	0.981	0.983	0.984	0.984
BGF	RMSE	1.690	2.067	1.641	1.664	1.585	1.623	1.623	1.549	1.634	2.052	1.989	2.069	2.132	2.017	2.077	2.129	2.074	1.949	1.894	2.092	2.116	2.116	2.652	2.208	2.208	2.151	2.150	2.150	2.150	2.0008	1.901	1.888	1.847
	NMB	0.03587	0.0660	0.0122	-0.0074	0.0036	-0.0058	-0.0058	0.0108	-0.0053	-0.0132	0.0329	-0.0513	-0.0628	-0.0412	-0.0098	0.0220	0.0334	-0.0006	-0.0118	-0.0705	0.0173	0.0173	-0.1128	0.0260	0.0260	-0.0167	-0.0124	-0.0009	-0.0009	0.0199	0.0303	-0.0204	0.0067
	Corr	0.985	0.982	0.984	0.984	0.985	0.985	0.984	0.986	0.984	0.980	0.982	0.983	0.983	0.983	0.980	0.974	0.975	0.979	0.980	0.980	0.977	0.977	0.975	0.975	0.975	0.976	0.976	0.975	0.975	0.982	0.984	0.985	0.984
BNT	RMSE	1.712	1.760	1.832	1.999	1.857	1.924	1.924	1.753	1.953	2.228	2.015	2.438	2.598	2.357	2.217	2.152	2.025	2.125	2.136	2.521	2.202	2.202	3.103	2.247	2.247	2.339	2.324	2.296	2.296	2.099	1.946	2.182	2.020
	NMB	-0.0134	0.0056	-0.0380	-0.0586	-0.0447	-0.0576	-0.0576	-0.0382	-0.0555	-0.0469	-0.0016	-0.0873	-0.0991	-0.0768	-0.0435	-0.0047	0.0039	-0.0441	-0.0580	-0.1203	-0.0179	-0.0179	-0.1567	-0.0106	-0.0106	-0.0535	-0.0490	-0.0415	-0.0415	-0.0207	-0.0089	-0.0616	-0.0344
	Corr	0.985	0.983	0.98	0.984	0.986	0.985	0.985	0.986	0.984	0.980	0.982	0.982	0.982	0.982	0.980	0.974	0.975	0.979	0.980	0.980	0.976	0.976	0.974	0.975	0.975	0.976	0.976	0.974	0.974	0.982	0.984	0.985	0.985
BP3	RMSE	1.614	2.041	1.600	1.676	1.564	1.632	1.632	1.517	1.645	2.175	2.069	2.139	2.120	2.098	2.214	2.062	2.120	1.971	1.958	2.194	2.134	2.134	2.704	2.219	2.219	2.196	2.196	2.237	2.237	1.899	1.802	1.812	1.741
	NMB	0.0188	0.0511	-0.0043	-0.00243	-0.0125	-0.0229	-0.0229	-0.0048	-0.0217	-0.0061	0.0395	-0.0426	-0.0559	-0.0326	-0.0027	0.0145	0.0270	-0.0156	-0.0280	-0.0869	0.0131	0.0131	-0.1199	0.0211	0.0211	-0.0214	-0.0170	-0.0003	-0.0003	0.0063	0.0170	-0.0344	-0.0067
	Corr	0.985	0.981	0.985	0.984	0.985	0.985	0.985	0.986	0.984	0.978	0.981	0.981	0.980	0.980	0.978	0.975	0.974	0.978	0.979	0.980	0.976	0.976	0.975	0.974	0.974	0.975	0.975	0.973	0.973	0.982	0.984	0.985	0.985
BWDM6	RMSE	3.314	3.733	3.096	3.002	3.016	2.981	2.981	3.038	2.992	3.057	3.513	2.764	2.685	2.818	3.097	3.296	3.397	3.211	3.048	2.741	3.513	3.513	2.644	3.568	3.568	3.246	3.291	3.288	3.288	3.627	3.629	3.230	3.413
	NMB	0.1436	0.1707	0.1228	0.1053	0.1140	0.1083	0.1083	0.1195	0.1065	0.0963	0.1406	0.0640	0.0522	0.0730	0.0993	0.1388	0.1564	0.1226	0.1157	0.0654	0.1363	0.1363	0.0239	0.1471	0.1471	0.1088	0.1126	0.1178	0.1178	0.1571	0.1641	0.1205	0.1459
	Corr	0.960	0.956	0.962	0.960	0.962	0.961	0.961	0.962	0.960	0.969	0.970	0.969	0.969	0.969	0.969	0.962	0.959	0.960	0.963	0.961	0.962	0.962	0.964	0.961	0.961	0.961	0.960	0.960	0.960	0.959	0.962	0.961	0.962
NG3D	RMSE	2.331	2.307	2.077	1.933	2.003	1.906	1.906	2.013	1.968	2.389	2.729	2.196	1.903	2.238	2.443	2.607	2.213	2.312	2.174	1.981	2.526	2.526	2.372	2.625	2.625	2.306	2.338	2.245	2.245	2.369	2.479	2.005	2.065
	NMB	0.1073	0.0860	0.0815	0.0632	0.0787	0.0631	0.0631	0.0798	0.0666	0.0547	0.0987	0.0239	0.0018	0.0332	0.0578	0.0995	0.0503	0.0658	0.0511	-0.0055	0.0732	0.0732	-0.0525	0.0814	0.0814	0.0370	0.0411	0.0166	0.0166	0.0936	0.1111	0.0647	0.0725
	Corr	0.983	0.981	0.983	0.982	0.983	0.983	0.983	0.984	0.982	0.979	0.979	0.980	0.981	0.979	0.979	0.972	0.973	0.975	0.977	0.978	0.974	0.974	0.972	0.972	0.972	0.974	0.974	0.973	0.973	0.980	0.982	0.983	0.983
NGF	RMSE	2.242	2.213	1.997	1.857	1.913	1.844	1.844	1.931	1.876	2.146	2.376	2.029	1.881	2.040	2.194	2.461	2.091	2.219	2.073	1.914	2.440	2.440	2.361	2.511	2.511	2.232	2.262	2.187	2.187	2.244	2.310	1.907	1.950
	NMB	0.1026	0.0802	0.0767	0.0584	0.0730	0.0581	0.0581	0.0749	0.0603	0.0324	0.0747	-0.0016	-0.0211	0.0080	0.0356	0.0889	0.0386	0.0600	0.0450	-0.0108	0.0672	0.0672	-0.0616	0.0728	0.0728	0.0301	0.0342	0.0110	0.0110	0.0813	0.0974	0.0503	0.0606
	Corr	0.984	0.982	0.984	0.983	0.984	0.984	0.984	0.984	0.983	0.980	0.980	0.981	0.982	0.980	0.980	0.974	0.975	0.977	0.979	0.979	0.975	0.975	0.974	0.973	0.973	0.9749	0.975	0.974	0.974	0.981	0.983	0.984	0.984
NNT	RMSE	1.832	1.786	1.703	1.731	1.682	1.679	1.679	1.639	1.724	2.097	2.111	2.218	2.270	2.171	2.105	2.307	2.044	2.055	1.975	2.122	2.266	2.266	2.686	2.313	2.313	2.213	2.224	2.275	2.275	2.062	2.029	1.895	1.858
	NMB	0.0448	0.0184	0.0202	0.0007	0.0175	7.5350	7.5350	0.0167	0.0022	-0.0076	0.0308	-0.0482	-0.0631	-0.0381	-0.0043	0.0651	0.0105	0.0117	-0.0034	-0.0669	0.0277	0.0277	-0.1089	0.0338	0.0338	-0.0111	-0.0068	-0.0318	-0.0318	0.0393	0.0542	0.0048	0.0196
	Corr	0.983	0.982	0.984	0.983	0.984	0.984	0.984	0.984	0.983	0.979	0.979	0.979	0.982	0.979	0.979	0.973	0.974	0.977	0.978	0.979	0.974	0.974	0.973	0.972	0.972	0.974	0.974	0.974	0.974	0.981	0.983	0.983	0.984
NP3	RMSE	2.130	2.179	1.876	1.776	1.795	1.758	1.758	1.829	1.805	2.375	2.631	2.223	1.949	2.244	2.435	2.427	2.142	2.169	2.073	1.982	2.463	2.463	2.449	2.570	2.570	2.298	2.324	2.275	2.275	2.160	2.255	1.848	1.855
	NMB	0.0851	0.0651	0.0581	0.0394	0.0552	0.0395	0.0395	0.0570	0.0428	0.0431	0.0875	0.0108	-0.0104	0.0203	0.0462	0.0826	0.0312	0.0433	0.0278	-0.0288	0.0623	0.0623	-0.0670	0.0685	0.0685	0.0239	0.0281	0.0065	0.0065	0.0674	0.0856	0.0378	0.0463
	Corr	0.983	0.981	0.984	0.983	0.984	0.984	0.984	0.984	0.983	0.978	0.978	0.979	0.980	0.979	0.978	0.975	0.974	0.977	0.978	0.979	0.974	0.974	0.973	0.971	0.971	0.974	0.973	0.973	0.973	0.982	0.983	0.984	0.984
NWDM6	RMSE	4.057	4.086	3.812	3.644	3.748	3.638	3.637	3.743	3.668	3.622	4.135	3.252	3.027	3.337	3.670	4.024	3.588	3.780	3.547	3.056	4.077	4.077	2.876	4.086	4.086	3.682	3.737	3.443	3.443	4.226	4.364	3.816	3.930
	NMB	0.2112	0.1918	0.1871	0.1710	0.1836	0.1721	0.1721	0.1846	0.1730	0.1423	0.1827	0.1122	0.0947	0.1207	0.1452	0.2053	0.1672	0.1803	0.1689	0.1211	0.1821	0.1821	0.0744	0.1912	0.1912	0.1523	0.1559	0.1288	0.1288	0.2174	0.2317	0.1916	0.1994
	Corr	0.956	0.950	0.956	0.954	0.955	0.956	0.955	0.956	0.953	0.966	0.965	0.965	0.966	0.965	0.966	0.957	0.955	0.958	0.961	0.960	0.959	0.959	0.960	0.958	0.958	0.958	0.958	0.958	0.958	0.955	0.958	0.958	0.958
SG3D	RMSE	1.979	2.113	1.830	1.865	1.828	1.847	1.847	1.808	1.861	2.748	2.501	2.548	2.472	2.509	2.804	2.217	2.134	2.288	2.191	2.405	2.415	2.415	2.978	2.552	2.552	2.496	2.506	2.242	2.242	2.177	2.139	2.067	1.921
	NMB	0.0092	0.0583	-0.0087	-0.0288	-0.0206	-0.0265	-0.0264	-0.0095	-0.0260	-0.0031	0.0419	-0.0375	-0.0523	-0.0276	0.0002	0.0010	0.0379	-0.0161	-0.0250	-0.0849	0.0159	0.0159	-0.1247	0.0211	0.0211	-0.0183	-0.0140	0.0014	0.0014	0.0016	0.0089	-0.0428	-0.0073
	Corr	0.980	0.982	0.983	0.982	0.983	0.983	0.983	0.982	0.982	0.971	0.975	0.976	0.974	0.976	0.971	0.975	0.975	0.975	0.977	0.976	0.972	0.972	0.970	0.967	0.967	0.970	0.970	0.974	0.974	0.979	0.981	0.981	0.983
SGF	RMSE	1.944	2.067	1.805	1.839	1.807	1.839	1.839	1.772	1.831	2.649	2.265	2.524	2.523	2.462	2.696	2.155	2.034	2.224	2.131	2.394	2.343	2.343	3.003	2.483	2.483	2.468	2.471	2.190	2.190	2.096	2.030	2.058	1.848
	NMB	0.0028	0.0576	-0.0143	-0.0345	-0.0270	-0.0319	-0.0319	-0.0157	-0.0319	-0.0249	0.0201	-0.0609	-0.0753	-0.0507	-0.0216	-0.0120	0.0252	-0.0253	-0.0338	-0.0945	0.0051	0.0051	-0.1338	0.0091	0.0091	-0.0304	-0.0260	-0.0055	-0.0055	-0.0139	-0.0070	-0.0596	-0.0227
	Corr	0.981	0.983	0.984	0.983	0.984	0.984	0.984	0.984	0.984	0.972	0.977	0.978	0.976	0.977	0.972	0.976	0.976	0.977	0.979	0.978	0.973	0.973	0.971	0.968	0.968	0.971	0.971	0.975	0.975	0.980	0.983	0.982	0.984
SNT	RMSE	1.894	1.747	1.890	2.069	1.968	2.031	2.031	1.878	2.040	2.676	2.160	2.729	2.820	2.636	2.695	2.126	2.002	2.302	2.268	2.728	2.347	2.347	3.374	2.463	2.463	2.596	2.583	2.334	2.334	2.127	2.022	2.289	1.964
	NMB	-0.0493	-0.0061	0.0677	-0.0889	-0.0792	-0.0867	-0.0867	-0.0679	-0.0859	-0.0600	-0.0148	-0.0973	-0.1120	-0.0868	-0.0565	-0.0370	-0.0041	-0.0697	-0.0811	-0.1462	-0.0316	-0.0316	-0.1784	-0.0284	-0.0284	-0.0695	-0.0649	-0.0503	-0.0503	-0.0555	-0.0473	-0.1019	-0.0652
	Corr	0.982	0.983	0.985	0.984	0.984	0.985	0.985	0.984	0.984	0.973	0.977	0.977	0.976	0.977	0.973	0.977	0.976	0.977	0.980	0.979	0.972	0.972	0.970	0.968	0.968	0.971	0.971	0.974	0.974	0.981	0.983	0.982	0.984
SP3	RMSE	2.049	2.078	1.927	1.996	1.945	1.981	1.981	1.894	1.987	2.850	2.500	2.682	2.620	2.632	2.904	2.317	2.121	2.397	2.313	2.588	2.488	2.488	3.160	2.633	2.633	2.634	2.637	2.303	2.303	2.208	2.175	2.120	1.975
	NMB	-0.0102	0.0406	-0.0273	-0.0478	-0.0392	-0.0457	-0.0457	-0.0280	-0.0447	-0.0154	0.0297	-0.05059	-0.0652	-0.0405	-0.0121	-0.0135	0.0205	-0.0363	-0.0452	-0.1073	0.0009	0.0009	-0.1402	0.0047	0.0047	-0.0349	-0.0305	-0.0093	-0.0093	-0.0210	-0.0136	0.0665	-0.0305
	Corr	0.979	0.981	0.982	0.981	0.982	0.982	0.982	0.982	0.981	0.969	0.974	0.975	0.973	0.974	0.969	0.973	0.974	0.973	0.976	0.975	0.970	0.970	0.968	0.965	0.965	0.970	0.968	0.973	0.973	0.979	0.981	0.981	0.983
SWDM6	RMSE	2.988	3.536	2.732	2.665	2.642	2.669	2.669	2.710	2.669	3.125	3.213	2.779	2.632	2.799	3.186	2.877	3.263	2.940	2.791	2.591	3.220	3.220	2.708	3.309	3.309	3.085	3.128	3.038	3.038	3.159	3.141	2.795	2.928
	NMB	0.0869	0.1520	0.0712	0.0527	0.0571	0.0568	0.0568	0.0695	0.0553	0.0627	0.1065	0.0343	0.0173	0.0436	0.0658	0.0798	0.1436	0.0764	0.0741	0.0195	0.1031	0.1031	-0.0204	0.1094	0.1094	0.0758	0.0798	0.0975	0.0975	0.0944	0.0975	0.0504	0.0890
	Corr	0.958	0.958	0.963	0.961	0.962	0.962	0.962	0.962	0.961	0.962	0.966	0.966	0.964	0.966	0.962	0.960	0.960	0.961	0.965	0.963	0.960	0.960	0.961	0.957	0.957	0.957	0.957	0.963	0.963	0.960	0.964	0.963	0.964

Table 4: Statistical analysis showing root mean square error (RMSE), normalized mean bias (NMB) and Pearson correlation (Cor) between each simulation and each of the stations considered for T2min. The abbreviations used in the Table are explained in the introduction of this section.

**Sims**	**Stat**	**BU**	**KA**	**LR**	**LF**	**MI**	**RU**	**SC**	**SP**	**ZE**	**AL**	**BE**	**FE**	**LA**	**PE**	**UJ**	**BO**	**FL**	**MU**	**KR**	**LI**	**RZ**	**FA**	**IS**	**JU**	**MB**	**MBII**	**ST**	**SR**	**UN**	**UNII**	**BA**	**FI**	**OB**	**OK**
	RMSE	2.983	3.151	2.483	3.050	2.810	2.952	2.952	2.448	2.962	3.244	2.074	3.133	1.928	2.823	3.558	2.339	2.639	2.319	2.489	2.368	2.708	1.896	1.896	2.096	3.420	3.420	2.484	2.499	1.998	1.998	2.855	2.497	2.751	2.754
BG3D	NMB	0.3027	-0.3830	-0.1639	-0.3247	-0.2525	-0.3199	-0.3199	-0.1851	-0.3099	-0.2827	-0.0755	-0.3211	-0.0881	-0.2522	-0.3667	-0.0983	0.0113	-0.0637	-0.0795	-0.1003	-0.2862	0.0631	0.0631	-0.1874	-0.2869	-0.2869	-0.1561	-0.1539	0.0137	0.0137	-0.1788	-0.1076	-0.1863	-0.1955
	Corr	0.956	0.936	0.966	0.961	0.961	0.960	0.960	0.964	0.962	0.958	0.975	0.968	0.980	0.970	0.960	0.967	0.937	0.964	0.959	0.961	0.961	0.977	0.977	0.978	0.930	0.930	0.962	0.961	0.966	0.966	0.961	0.972	0.969	0.964
BGF	RMSE	3.033	3.149	2.527	3.090	2.864	2.985	2.985	2.484	3.004	3.374	2.185	3.267	2.038	2.950	3.691	2.384	2.642	2.382	2.536	2.401	2.752	1.897	1.897	2.143	3.480	3.480	2.519	2.534	1.994	1.994	2.904	2.541	2.806	2.796
	NMB	-0.3107	-0.3874	-0.1680	-0.3294	-0.2599	-0.3270	-0.3270	-0.1903	-0.3166	-0.2976	-0.0884	-0.3391	-0.1011	-0.2692	-0.3826	-0.1082	0.0074	-0.0769	-0.0847	-0.1077	-0.2947	0.0569	0.0569	-0.1949	-0.2963	-0.2963	-0.1618	-0.1596	0.0114	0.0114	-0.1907	-0.1196	-0.1991	-0.2074
	Corr	0.955	0.937	0.965	0.961	0.961	0.960	0.960	0.964	0.962	0.958	0.975	0.968	0.981	0.970	0.960	0.968	0.938	0.965	0.958	0.961	0.961	0.977	0.977	0.978	0.930	0.930	0.963	0.962	0.967	0.967	0.961	0.973	0.970	0.964
BNT	RMSE	3.246	3.248	2.753	3.345	3.111	3.213	3.213	2.701	3.268	3.653	2.470	3.589	2.338	3.273	4.003	2.571	2.667	2.503	2.746	2.574	2.981	1.968	1.968	2.356	3.712	3.712	2.738	2.751	2.095	2.095	3.107	2.754	3.028	2.980
	NMB	-0.3368	-0.3832	-0.1965	-0.3618	-0.2911	-0.3527	-0.3526	-0.2166	-0.3467	-0.3359	-0.1271	-0.3773	-0.1367	-0.3053	-0.4234	-0.1451	-0.0048	-0.1003	-0.1201	-0.1386	-0.3358	0.0275	0.0275	-0.2354	-0.3374	-0.3374	-0.1930	-0.1908	-0.0169	-0.0169	-0.2191	-0.1460	-0.2273	-0.2317
	Corr	0.954	0.935	0.965	0.960	0.960	0.959	0.959	0.963	0.960	0.958	0.975	0.966	0.980	0.968	0.960	0.967	0.938	0.964	0.958	0.961	0.960	0.977	0.977	0.979	0.928	0.928	0.961	0.960	0.966	0.966	0.961	0.973	0.969	0.964
BP3	RMSE	2.971	3.114	2.366	3.047	2.779	2.959	2.959	2.372	2.969	3.410	2.104	3.313	2.003	2.985	3.732	2.186	2.345	2.119	2.321	2.221	2.781	1.660	1.660	2.204	3.475	3.475	2.525	2.537	1.900	1.900	2.746	2.278	2.634	2.681
	NMB	-0.3738	-0.5045	-0.2275	-0.3971	-0.3145	-0.3985	-0.3985	-0.2493	-0.3830	-0.3236	-0.1102	-0.3657	-0.1206	-0.2944	-0.4103	-0.1788	-0.1315	-0.1470	-0.1491	-0.1831	-0.3778	0.0258	0.0258	-0.2428	-0.3464	-0.3464	-0.2101	-0.2079	-0.0332	-0.0332	-0.2743	-0.1926	-0.2773	-0.2962
	Corr	0.962	0.945	0.972	0.968	0.968	0.968	0.968	0.972	0.968	0.959	0.976	0.969	0.981	0.971	0.962	0.973	0.948	0.970	0.965	0.968	0.966	0.979	0.979	0.981	0.935	0.935	0.966	0.965	0.969	0.969	0.966	0.978	0.974	0.969
BWDM6	RMSE	2.637	2.835	2.280	2.591	2.496	2.541	2.541	2.206	2.516	2.767	2.288	2.581	2.067	2.396	2.948	2.339	2.629	2.356	2.508	2.283	2.345	2.690	2.690	1.935	3.050	3.050	2.340	2.361	2.332	2.332	2.666	2.578	2.616	2.533
	NMB	-0.1492	-0.2349	-0.0148	-0.1550	-0.0952	-0.1544	-0.1544	-0.0466	-0.1446	-0.1260	0.0639	-0.1613	0.0451	-0.1006	-0.1998	0.0909	0.1699	0.1094	0.0764	0.0641	-0.0864	0.1975	0.1975	-0.0029	-0.0873	-0.0873	0.0117	0.0135	0.1485	0.1485	0.0275	0.0859	0.0210	0.0124
	Corr	0.951	0.935	0.960	0.956	0.954	0.954	0.954	0.960	0.957	0.952	0.968	0.962	0.973	0.963	0.954	0.965	0.940	0.963	0.953	0.959	0.957	0.970	0.970	0.970	0.926	0.926	0.956	0.955	0.963	0.963	0.956	0.964	0.962	0.959
NG3D	RMSE	2.271	2.794	2.537	2.174	2.610	2.149	2.149	2.654	2.251	2.125	2.351	1.918	2.441	2.063	1.876	2.784	2.629	2.869	3.1514	2.635	2.049	3.416	3.416	1.793	2.612	2.612	2.147	2.175	2.126	2.126	2.642	2.965	2.705	2.437
	NMB	0.1758	-0.2379	0.2572	0.1546	0.2424	0.1454	0.1454	0.2602	0.1698	0.0751	0.1878	0.0643	0.2021	0.1131	0.0145	0.3039	0.0578	0.3253	0.3296	0.2686	0.1086	0.3057	0.3057	0.0477	-0.0339	-0.0339	0.1150	0.1166	0.0955	0.0955	0.2463	0.3265	0.2787	0.1891
	Corr	0.963	0.939	0.967	0.965	0.959	0.964	0.964	0.958	0.964	0.965	0.976	0.971	0.979	0.971	0.971	0.963	0.938	0.962	0.954	0.960	0.965	0.969	0.969	0.973	0.938	0.938	0.965	0.964	0.964	0.964	0.964	0.968	0.968	0.965
NGF	RMSE	2.230	2.787	2.494	2.141	2.551	2.113	2.113	2.598	2.202	2.119	2.333	1.897	2.393	2.038	1.890	2.730	2.613	2.816	3.098	2.581	2.018	3.400	3.400	1.761	2.607	2.607	2.122	2.150	2.105	2.105	2.593	2.908	2.645	2.383
	NMB	0.1645	-0.2578	0.2462	0.1420	0.2317	0.1326	0.1326	0.2516	0.1581	0.0670	0.1832	0.0587	0.1950	0.1078	0.0059	0.2970	0.0506	0.3188	0.3203	0.2590	0.0984	0.3058	0.3058	0.0463	-0.03669	-0.03669	0.1160	0.1176	0.0962	0.0962	0.2373	0.3190	0.2707	0.1783
	Corr	0.963	0.940	0.966	0.965	0.959	0.964	0.964	0.959	0.964	0.965	0.976	0.972	0.980	0.971	0.971	0.964	0.939	0.963	0.955	0.961	0.966	0.970	0.970	0.973	0.939	0.939	0.965	0.965	0.965	0.965	0.965	0.968	0.969	0.966
NNT	RMSE	2.408	2.873	2.639	2.277	2.704	2.239	2.239	2.780	2.365	2.123	2.250	1.923	2.314	2.049	1.947	2.741	2.657	2.837	3.159	2.643	2.095	3.305	3.305	1.753	2.733	2.733	2.145	2.175	2.117	2.117	2.701	3.036	2.782	2.481
	NMB	0.1966	-0.2189	0.2710	0.1703	0.2600	0.1636	0.1636	0.2851	0.1901	0.0535	0.1641	0.0458	0.1791	0.0956	-0.0085	0.292	0.0538	0.3144	0.3282	0.2664	0ñ.0927	0.2897	0.2897	0.0185	-0.0649	-0.0649	0.0953	0.0969	0.0752	0.0752	0.2571	0.3369	0.2898	0.1947
	Corr	0.962	0.938	0.967	0.965	0.960	0.964	0.964	0.959	0.964	0.966	0.977	0.972	0.980	0.971	0.971	0.964	0.938	0.963	0.956	0.962	0.965	0.970	0.970	0.975	0.936	0.936	0.965	0.964	0.965	0.965	0.965	0.968	0.968	0.966
NP3	RMSE	1.727	2.656	1.954	1.642	2.033	1.649	1.649	2.101	1.691	2.023	2.102	1.837	2.197	1.919	1.821	2.211	2.281	2.304	2.596	2.088	1.734	3.042	3.042	1.693	2.517	2.517	1.891	1.920	1.923	1.923	2.059	2.277	2.038	1.939
	NMB	0.0850	-0.3669	0.1776	0.0640	0.1605	0.0507	0.0507	0.1786	0.0776	0.0277	0.1493	0.0134	0.1639	0.0649	-0.0360	0.2133	-0.0896	0.2342	0.2485	0.1766	0.0096	0.2632	0.2632	-0.0122	-0.0994	-0.0994	0.0564	0.0582	0.0466	0.0466	0.1353	0.2239	0.1688	0.0750
	Corr	0.971	0.949	0.974	0.973	0.967	0.973	0.973	0.967	0.972	0.965	0.977	0.972	0.978	0.971	0.972	0.967	0.948	0.966	0.960	0.967	0.970	0.970	0.970	0.974	0.943	0.943	0.967	0.967	0.967	0.967	0.970	0.973	0.974	0.971
NWDM6	RMSE	2.459	2.581	2.871	2.399	2.910	2.381	2.381	2.917	2.466	2.574	3.216	2.380	3.214	2.630	2.198	3.311	2.629	3.392	3.592	3.010	2.358	4.226	4.226	2.325	2.727	2.727	2.656	2.683	2.625	2.625	3.084	3.484	3.168	2.828
	NMB	0.2465	-0.1252	0.3199	0.2260	0.3043	0.2177	0.2177	0.3160	0.2373	0.1824	0.2970	0.1806	0.2939	0.2233	0.1289	0.4243	0.2109	0.4409	0.4162	0.3653	0.2433	0.4033	0.4033	0.1989	0.1282	0.1282	0.2414	0.2428	0.2214	0.2214	0.3581	0.4258	0.3850	0.3092
	Corr	0.960	0.938	0.961	0.960	0.952	0.959	0.959	0.954	0.959	0.960	0.970	0.966	0.974	0.966	0.966	0.961	0.942	0.961	0.950	0.958	0.961	0.966	0.966	0.967	0.936	0.936	0.961	0.960	0.961	0.961	0.959	0.961	0.962	0.960
SG3D	RMSE	3.098	3.064	2.315	2.920	2.889	2.925	2.925	2.515	2.858	3.314	2.171	3.147	2.260	2.842	3.535	2.259	2.573	2.209	2.377	2.176	2.708	1.992	1.992	2.373	3.481	3.481	2.467	2.490	1.964	1.964	2.750	2.331	2.640	2.680
	NMB	-0.3901	-0.4042	-0.2158	-0.3838	-0.3190	-0.3739	-0.3739	-0.2386	-0.3704	-0.2699	-0.0775	-0.3019	-0.0764	-0.2339	-0.3532	-0.1839	-0.0137	-0.1423	-0.1195	-0.1347	-0.3421	0.0595	0.0595	-0.2349	-0.3195	-0.3195	-0.1634	-0.1613	0.0036	0.0036	-0.2699	-0.1850	-0.2692	-0.2852
	Corr	0.944	0.938	0.964	0.958	0.954	0.956	0.956	0.957	0.958	0.943	0.964	0.957	0.965	0.959	0.945	0.958	0.938	0.954	0.948	0.958	0.955	0.967	0.967	0.972	0.916	0.916	0.953	0.952	0.965	0.965	0.956	0.966	0.962	0.960
SGF	RMSE	3.169	3.103	2.369	2.986	2.946	3.007	3.007	2.549	2.916	3.399	2.173	3.181	2.229	2.871	3.614	2.243	2.584	2.177	2.310	2.118	2.721	1.885	1.885	2.377	3.519	3.519	2.468	2.488	1.940	1.940	2.765	2.313	2.660	2.709
	NMB	-0.4222	-0.4193	-0.2424	-0.4140	-0.3500	-0.4051	-0.4051	-0.2667	-0.4005	-0.2944	-0.0986	-0.3276	-0.0980	-0.2583	-0.3792	-0.2134	-0.0196	-0.1717	-0.1457	-0.1606	-0.3729	0.0455	0.0455	-0.2391	-0.3399	-0.3399	-0.1793	-0.1771	-0.0020	-0.0020	-0.2990	0.2150	0.3013	-0.3144
	Corr	0.947	0.937	0.967	0.962	0.960	0.959	0.959	0.961	0.963	0.946	0.967	0.964	0.970	0.965	0.950	0.964	0.938	0.960	0.955	0.963	0.961	0.970	0.970	0.972	0.918	0.918	0.956	0.955	0.966	0.966	0.960	0.971	0.966	0.964
SNT	RMSE	3.253	3.147	2.424	3.055	3.031	3.043	3.043	2.631	2.992	3.492	2.211	3.303	2.261	2.984	3.736	2.328	2.610	2.237	2.355	2.156	2.814	1.815	1.815	2.486	3.656	3.656	2.555	2.575	2.004	2.004	2.819	2.361	2.718	2.747
	NMB	-0.4370	-0.4063	-0.2551	-0.4285	-0.3674	-0.4138	-0.4138	-0.2782	-0.4142	-0.3194	-0.1240	-0.3505	-0.1203	-0.2800	-0.4058	-0.2375	-0.0260	-0.1860	-0.1652	-0.1765	-0.391	0.0236	0.0236	-0.2721	-0.3738	-0.3738	-0.2037	-0.2015	-0.0275	-0.0275	-0.3161	-0.2328	-0.3203	-0.3287
	Corr	0.946	0.936	0.967	0.962	0.959	0.959	0.959	0.959	0.962	0.948	0.969	0.963	0.972	0.96	0.951	0.964	0.937	0.959	0.955	0.964	0.960	0.971	0.971	0.973	0.918	0.918	0.956	0.955	0.965	0.965	0.961	0.972	0.967	0.964
SP3	RMSE	3.288	3.111	2.513	3.152	3.090	3.161	3.161	2.701	3.085	3.542	2.264	3.332	2.378	3.023	3.763	2.443	2.360	2.371	2.455	2.249	2.942	1.882	1.882	2.605	3.651	3.651	2.614	2.631	1.927	1.927	2.942	2.485	2.845	2.898
	NMB	-0.4565	-0.5216	-0.2780	-0.4546	-0.3794	-0.4509	-0.4509	-0.3019	-0.4408	-0.3203	-0.1173	-0.3513	-0.1164	-0.2808	-0.4068	-0.2605	-0.1454	-0.2261	-0.1908	-0.2162	-0.4366	0.0164	0.0164	-0.2838	-0.3876	-0.3876	-0.2235	-0.2213	-0.0426	-0.0426	-0.3473	-0.2545	-0.3436	-0.3676
	Corr	0.949	0.945	0.969	0.964	0.960	0.963	0.963	0.962	0.964	0.947	0.968	0.964	0.969	0.965	0.951	0.963	0.947	0.961	0.956	0.967	0.963	0.970	0.970	0.972	0.920	0.920	0.958	0.957	0.966	0.966	0.961	0.971	0.966	0.965
SWDM6	RMSE	2.763	2.784	2.043	2.505	2.522	2.577	2.577	2.219	2.452	2.890	2.141	2.630	2.169	2.418	3.003	1.998	2.533	1.997	2.179	1.971	2.300	2.454	2.454	2.028	3.086	3.086	2.238	2.264	2.203	2.203	2.447	2.124	2.336	2.383
	NMB	-0.3013	-0.2802	-0.1267	-0.2823	-0.2302	-0.2757	-0.2757	-0.1597	-0.2707	-0.1613	0.0226	-0.1854	0.0170	-0.1235	0.2374	-0.0604	0.1326	-0.0349	-0.0190	-0.0233	-0.2038	0.1587	0.1587	-0.0814	-0.1699	-0.1699	-0.0375	-0.0356	0.1233	0.1233	-0.1442	-0.0694	-0.1453	-0.1526
	Corr	0.944	0.9361	0.963	0.958	0.955	0.955	0.955	0.959	0.959	0.942	0.961	0.958	0.964	0.959	0.945	0.957	0.941	0.957	0.951	0.961	0.956	0.966	0.966	0.967	0.914	0.914	0.951	0.950	0.963	0.963	0.956	0.965	0.960	0.959

Table 5: Statistical analysis showing root mean square error (RMSE), normalized mean bias (NMB) and Pearson correlation (Cor) between each simulation and each of the stations considered for specific humidity. The abbreviations used in the Table are explained in the introduction of this section.

Sims	Stat	BU	KA	LR	LF	MI	RU	SC	SP	ZE	AL	BE	FE	LA	PE	UJ	BO	FL	KR	LI	RZ	FA	IS	JU	MB	MBII	ST	SR	UN	UNII	BA	FI	OB	OK
	RMSE	0,001	0.030	0.001	0.001	0.001	0.001	0.001	0.001	0.001	0.002	0.001	0.001	0.001	0.001	0.001	0.002	0.001	0.001	0.001	0.001	0.001	0.001	0.002	0.002	0.002	0.002	0.002	0.002	0.002	0.001	0.001	0.001	0.002
BG3D	NMB	0,1791	-0.1427	0.1804	0.1904	0.1802	0.1886	0.1894	0.1689	0.1872	0.2100	0.1403	0.1644	0.1681	0.16959	0.1662	0.2179	0.1906	0.1862	0.1899	0.0236	0.0393	0.0217	0.2047	0.2047	0.188	0.1695	0.1695	0.1739	0.1731	0.1551	0.1551	0.1514	0.1891
	Corr	0,961	0.061	0.959	0.960	0.960	0.959	0.959	0.963	0.961	0.956	0.946	0.956	0.953	0.954	0.954	0.954	0.959	0.970	0.959	0.970	0.979	0.975	0.922	0.922	0.944	0.930	0.930	0.939	0.939	0.940	0.940	0.942	0.954
BGF	RMSE	0.001	0.030	0.001	0.001	0.001	0.001	0.001	0.001	0.001	0.002	0.001	0.001	0.002	0.002	0.002	0.002	0.002	0.001	0.002	0.001	0.001	0.001	0.002	0.002	0.002	0.002	0.002	0.002	0.002	0.002	0.002	0.001	0.002
	NMB	0.1775	-0.1447	0.1791	0.1891	0.1787	0.1873	0.1880	0.1670	0.1862	0.2280	0.1606	0.1787	0.1880	0.1854	0.1861	0.2357	0.2077	0.1859	0.1957	0.0232	0.0385	0.0208	0.2130	0.2130	0.1962	0.1778	0.1778	0.1829	0.1821	0.1620	0.1620	0.1581	0.1955
	Corr	0.962	0.060	0.960	0.963	0.960	0.961	0.961	0.964	0.963	0.954	0.950	0.923	0.957	0.956	0.957	0.953	0.958	0.971	0.962	0.971	0.981	0.976	0.922	0.922	0.944	0.932	0.932	0.939	0.939	0.940	0.940	0.945	0.956
BNT	RMSE	0.002	0.030	0.002	0.002	0.002	0.002	0.002	0.002	0.002	0.002	0.002	0.002	0.002	0.002	0.002	0.002	0.002	0.001	0.002	0.001	0.001	0.001	0.002	0.002	0.002	0.002	0.002	0.002	0.002	0.002	0.002	0.002	0.002
	NMB	0.2259	-0.0837	0.2284	0.23678	0.2271	0.2355	0.2362	0.2173	0.2351	0.2622	0.1983	0.2207	0.2258	0.2269	0.2240	0.2695	0.2093	0.1949	0.2076	0.0545	0.0695	0.0528	0.2393	0.2393	0.2222	0.2021	0.2021	0.2105	0.2097	0.1864	0.1864	0.1905	0.2265
	Corr	0.953	0.068	0.951	0.948	0.951	0.951	0.951	0.954	0.949	0.938	0.937	0,948	0.945	0.942	0.946	0.935	0.963	0.972	0.964	0.969	0.979	0.973	0.921	0.921	0.942	0.929	0.929	0.939	0.939	0.937	0.937	0.941	0.948
BP3	RMSE	0.001	0.030	0.001	0,001	0.001	0.001	0.001	0.001	0.001	0.002	0.001	0.001	0.001	0.001	0.001	0.002	0.001	0.001	0.001	0.001	0.001	0.001	0.002	0.002	0.002	0.002	0.002	0.002	0.002	0.001	0.001	0.001	0.002
	NMB	0.1748	-0.1571	0.1746	0,1847	0.1755	0.1822	0.1829	0.1630	0.1816	0.2038	0.1339	0.1560	0.1606	0.1592	0.1587	0.2117	0.1818	0.1670	0.1797	0.0112	0.0266	0.0090	0.1974	0.1974	0.1811	0.1619	0.1619	0.1661	0.1653	0.1433	0.1433	0.1416	0.1791
	Corr	0.958	0.064	0.956	0.957	0.956	0.955	0.955	0.961	0.958	0.953	0.944	0.956	0.951	0.952	0.952	0.951	0.957	0.971	0.957	0.0969	0.978	0.974	0.920	0.920	0.941	0.927	0.927	0.936	0.936	0.937	0.937	0.940	0.952
BWDM6	RMSE	0,001	0.030	0.001	0.001	0.001	0.001	0.001	0.001	0.001	0.002	0.001	0.002	0.002	0.002	0.002	0.002	0.002	0.002	0.002	0.001	0.001	0.001	0.002	0.002	0.002	0.002	0.002	0.002	0.002	0.002	0.002	0.001	0.002
	NMB	0,1902	-0.1242	0.1869	0.1968	0.1872	0.1952	0.1959	0.1780	0.1943	0.2339	0.1667	0.1881	0.1937	0.1909	0.1919	0.2410	0.2127	0.2155	0.2116	0.487	0.0634	0.0476	0.2280	0.2280	0.2134	0.1954	0.1954	0.2000	0.1991	0.1852	0.1852	0.1700	0.2078
	Corr	0,963	0.059	0.961	0.965	0.962	0.962	0.962	0.965	0.966	0.958	0.951	0.962	0.957	0.957	0.957	0.957	0.960	0.966	0.959	0.968	0.976	0.971	0.929	0.929	0.949	0.933	0.933	0.941	0.941	0.942	0.942	0.948	0.958
NG3D	RMSE	0.001	0.030	0.001	0.001	0.001	0.001	0.001	0.001	0.001	0.001	0.001	0.001	0.001	0.001	0.001	0.002	0.001	0.001	0,001	0.001	0.001	0.001	0.002	0.002	0.002	0.001	0.001	0.001	0.001	0.001	0.001	0.001	0.001
	NMB	0.1641	-0.1548	0.1501	0.1605	0.1534	0.1577	0.1585	0.1609	0.1663	0.1563	0.0996	0.1252	0.1240	0.1070	0.1220	0.1648	0.1761	0.1885	0.1829	-0.0304	-0.0103	-0.0261	0.1758	0.1758	0.1700	0.1510	0.1510	0.1505	0.1496	0.1503	0.1503	0.1228	0.1632
	Corr	0.965	0.059	0.964	0.969	0.964	0.966	0.966	0.962	0.969	0.969	0.954	0.961	0.964	0.962	0.963	0.968	0.962	0.970	0.962	0.969	0.978	0.974	0.926	0.926	0.946	0.934	0.934	0.944	0.943	0.941	0.941	0.949	0.962
NGF	RMSE	0.001	0.030	0.001	0,001	0.001	0.001	0.001	0.001	0.001	0.002	0.001	0.001	0.001	0.001	0.001	0.002	0.001	0.001	0.001	0.001	0.001	0,001	0.002	0.002	0.002	0.002	0.002	0.001	0.001	0.001	0.001	0.001	0.001
	NMB	0.1627	-0.1586	0.1469	0.1573	0.1507	0.1548	0.1556	0.1590	0.1642	0.1777	0.1204	0.1460	0.1472	0.1302	0.1452	0.1859	0.1789	0.1913	0.1859	-0.0341	-0.0132	-0.0300	0.1805	0.1805	0.1753	0.1567	0.1567	0.1553	0.1544	0.1549	0.1549	0.1258	0.1656
	Corr	0.966	0.059	0.964	0.970	0.964	0.966	0.966	0.963	0.970	0.967	0.955	0.964	0.964	0.963	0.963	0.965	0.964	0.970	0.964	0.971	0.980	0.975	0.928	0.928	0.949	0.936	0.936	0.945	0.945	0.942	0.942	0.950	0.962
NNT	RMSE	0.002	0.030	0.001	0.002	0.001	0.001	0.001	0.001	0.002	0.002	0.001	0.002	0.002	0.002	0.002	0.002	0.001	0.001	0.001	0.001	0.001	0.001	0.002	0.002	0.002	0.002	0.002	0.002	0.002	0.002	0.002	0.001	0.002
	NMB	0.2026	-0.0841	0.1894	0.1993	0.1931	0.1975	0.1983	0.2004	0.2052	0.2125	0.1554	0.1825	0.1837	0.1690	0.1818	0.2204	0.1826	0.1961	0.1891	-0.0002	0.0183	0.0045	0.2010	0.2010	0.1946	0.1736	0.1736	0.1766	0.1758	0.1770	0.1770	0.1496	0.1894
	Corr	0.961	0.070	0.960	0.961	0.960	0.962	0.962	0.958	0.960	0.949	0.941	0.952	0.950	0.949	0.949	0.947	0.966	0.972	0.965	0.969	0.979	0.974	0.924	0.924	0.946	0.933	0.933	0.943	0.943	0.939	0.939	0.948	0.956
NP3	RMSE	0,001	0.030	0.001	0.001	0.001	0.001	0.001	0.001	0.001	0.001	0.001	0.002	0.001	0.001	0.001	0.001	0.001	0.001	0.001	0.001	0.001	0.001	0.002	0.002	0.002	0.001	0.001	0.001	0.001	0.001	0.001	0.001	0.001
	NMB	0,1522	-0.1703	0.1375	0.1480	0.1415	0.1457	0.1465	0.1497	0.1542	0.1458	0.0882	0.1124	0.1132	0.0971	0.1112	0.1537	0.1592	0.1708	0.1656	-0.0470	-0.0263	-0.423	0.1636	0.1636	0.1589	0.1394	0.1394	0.1374	0.1366	0.1382	0.1382	0.1049	0.1474
	Corr	0,965	0.062	0.963	0.968	0.964	0.965	0.965	0.962	0.968	0.967	0.953	0.954	0.962	0.961	0.962	0.967	0.962	0.970	0.962	0.970	0.979	0.975	0.925	0.925	0.945	0.933	0.933	0.942	0.941	0.939	0.939	0.950	0.961
NWDM6	RMSE	0.001	0.030	0.001	0,001	0.001	0.001	0.001	0.001	0.001	0.002	0.001	0.001	0.001	0.001	0.001	0.002	0.002	0.002	0.002	0.001	0.001	0.001	0.002	0.002	0.002	0.002	0.002	0.002	0.002	0.002	0.002	0.001	0.002
	NMB	0.1813	-0.1305	0.1681	0.1782	0.1709	0.1755	0.1763	0.1765	0.1832	0.1944	0.1400	0.1629	0.1643	0.1479	0.1624	0.2024	0.2077	0.2187	0.2133	0.0081	0.0283	0.0126	0.2097	0.2097	0.2053	0.1869	0.1869	0.1859	0.1851	0.1854	0.1854	0.1553	0.1951
	Corr	0.961	0.059	0.958	0.965	0.959	0.961	0.961	0.958	0.964	0.965	0.951	0.955	0.959	0.959	0.959	0.965	0.958	0.965	0.958	0.962	0.969	0.966	0.927	0.927	0.947	0.934	0.934	0.941	0.941	0.941	0.941	0.946	0.957
SG3D	RMSE	0.001	0.030	0.001	0.001	0.001	0.001	0.001	0.001	0.001	0.002	0.001	0.001	0.001	0.001	0.001	0.002	0.001	0.001	0.001	0.001	0.001	0.001	0.002	0.002	0.002	0.002	0.002	0.002	0.002	0.001	0.001	0.001	0.002
	NMB	0.1742	-0.1438	0.1742	0.1843	0.1727	0.1828	0.1835	0.1640	0.1816	0.2261	0.1318	0.1561	0.1617	0.1572	0.1598	0.2098	0.1854	0.1845	0.1850	0.0163	0.0323	0.0149	0.2066	0.2000	0.1843	0.1653	0.1653	0.1696	0.1688	0.1531	0.1531	0.1417	0.1818
	Corr	0.962	0.061	0.960	0.962	0.960	0.959	0.959	0.963	0.962	0.957	0.948	0.957	0.955	0.954	0.956	0.955	0.961	0.970	0.961	0.969	0.978	0.974	0.923	0.922	0.943	0.929	0.929	0.939	0.939	0.939	0.939	0.943	0.956
SGF	RMSE	0.001	0.030	0.001	0,002	0.001	0.001	0.001	0.001	0.001	0.002	0.001	0.001	0.002	0.002	0.002	0.002	0.001	0.001	0.001	0.001	0.001	0.001	0.002	0.002	0.002	0.002	0.002	0.002	0.002	0.001	0.001	0.001	0.002
	NMB	0.1703	-0.1493	0.1691	0,1792	0.1674	0.1778	0.1786	0.1589	0.1766	0.02215	0.1540	0.1759	0.1837	0.1787	0.1818	0.2293	0.2077	0.1879	0.1883	0.0148	0.0305	0.0131	0.2066	0.2066	0.1905	0.1717	0.1717	0.1766	0.1758	0.1575	0.1575	0.1475	0.1868
	Corr	0.964	0.061	0.963	0.967	0.963	0.963	0.963	0.966	0.968	0.954	0.949	0.961	0.955	0.954	0.956	0.952	0.958	0.971	0.964	0.972	0.981	0.975	0.924	0.924	0.945	0.932	0.932	0.941	0.940	0.941	0.941	0.945	0.957
SNT	RMSE	0.002	0.030	0.001	0.002	0.002	0.002	0.002	0.002	0.002	0.002	0.002	0.002	0.002	0.002	0.002	0.002	0.002	0.001	0.002	0.001	0.001	0.001	0.002	0.002	0.002	0.002	0.002	0.002	0.002	0.002	0.002	0.001	0.002
	NMB	0.2207	-0.0999	0.2218	0.2313	0.2194	0.2299	0.2306	0.2114	0.2293	0.2580	0.1929	0.2185	0.2220	0.2170	0.2655	0.2654	0.2002	0.1936	0.1993	0.0471	0.0629	0.0468	0.2334	0.2334	0.2178	0.1971	0.1971	0.2052	0.2044	0.1841	0.1841	0.1797	0.2178
	Corr	0.954	0.063	0.952	0.949	0.952	0.952	0.952	0.955	0.950	0.937	0.936	0.946	0.943	0.942	0.934	0.934	0.964	0.973	0.965	0.969	0.978	0.972	0.921	0.921	0.941	0.929	0.929	0.939	0.939	0.938	0.938	0.941	0.949
SP3	RMSE	0.001	0.030	0.001	0.001	0.001	0.001	0.001	0.001	0.001	0.002	0.001	0.001	0.001	0.001	0.001	0.002	0.001	0.001	0.001	0.001	0.001	0.001	0.002	0.002	0.002	0.002	0.002	0.002	0.002	0.001	0.001	0.001	0.001
	NMB	0.1698	-0.1575	0.1685	0.1786	0.1676	0.1768	0.1776	0.1583	0.1761	0.1958	0.1255	0.1488	0.1556	0.1511	0.1537	0.2039	0.1766	0.1688	0.1756	0.0050	0.0205	0.0033	0.1934	0.1934	0.177	0.1580	0.1580	0.1628	0.1619	0.1435	0.1435	0.1336	0.1729
	Corr	0.959	0.064	0.957	0.959	0.957	0.956	0.956	0.961	0.960	0.953	0.945	0.954	0.951	0.952	0.953	0.951	0.960	0.970	0.961	0.969	0.978	0.973	0.920	0.920	0.941	0.927	0.927	0.937	0.937	0.937	0.937	0.942	0.953
SWDM6	RMSE	0.001	0.030	0.001	0.001	0.001	0.001	0.001	0.001	0.001	0.002	0.001	0.002	0.002	0.002	0.002	0.002	0.002	0.002	0.002	0.001	0.001	0.001	0.002	0.002	0.002	0.002	0.002	0.002	0,002	0.002	0.002	0.001	0.002
	NMB	0.1843	-0.1271	0.1808	0.1908	0.1795	0.1895	0.1902	0.1735	0.1886	0.2261	0.1589	0.1837	0.1889	0.1835	0.1870	0.2339	0.2070	0.2127	0.2059	0.0403	0.0553	0.0395	0.2225	0.2225	0.2073	0.1892	0.1892	0.1937	0.1929	0.1830	0.1830	0.1610	0.1006
	Corr	0.964	0.060	0.962	0.966	0.963	0.963	0.963	0.964	0.966	0.957	0.949	0.959	0.957	0.956	0.957	0.956	0.961	0.966	0.961	0.967	0.975	0.970	0.929	0.929	0.948	0.933	0.933	0.941	0.941	0.942	0.942	0.949	0.959

F Statistically significant spatial differences between urban and non-urban schemes for T2 and Q2

Fig. F.1 and Fig. F.2 show the statistically significant differences in multi-year seasonal average hourly temperature at 2 m (T2) for the winter (DJF) and summer (JJA) seasons, respectively. Urban areas show a strong signal compared to their rural counterparts, especially for those simulations using the SLUCM urban scheme, where the differences in T2 are higher than 1 K for both seasons. The choice of microphysics and convection schemes does not seem to have a significant impact on the simulation of the UHI.

Specific humidity values are also affected by the presence of cities, showing smaller values than for rural areas. This can be seen in Fig. F.3 and Fig. F.4 for each of the evaluated seasons. However, the reduction in specific humidity is stronger in JJA reaching values around $-1\,\mathrm{g\,kg^{-1}}$, which is an order of magnitude bigger than in DJF (around $-0.1\,\mathrm{g\,kg^{-1}}$). Besides, the impact of the selected microphysics and convection scheme is not relevant over cities during JJA season. In DJF, the bulk scheme seems to reduce specific humidity more than the SLUCM, and the simulation showing the highest reduction is the one using the GF convection scheme. In general, the SLUCM scheme leads to a small decrease in specific humidity over cities and a clear increase over rural areas up to 0.2 $\mathrm{g\,kg^{-1}}$ in DJF.

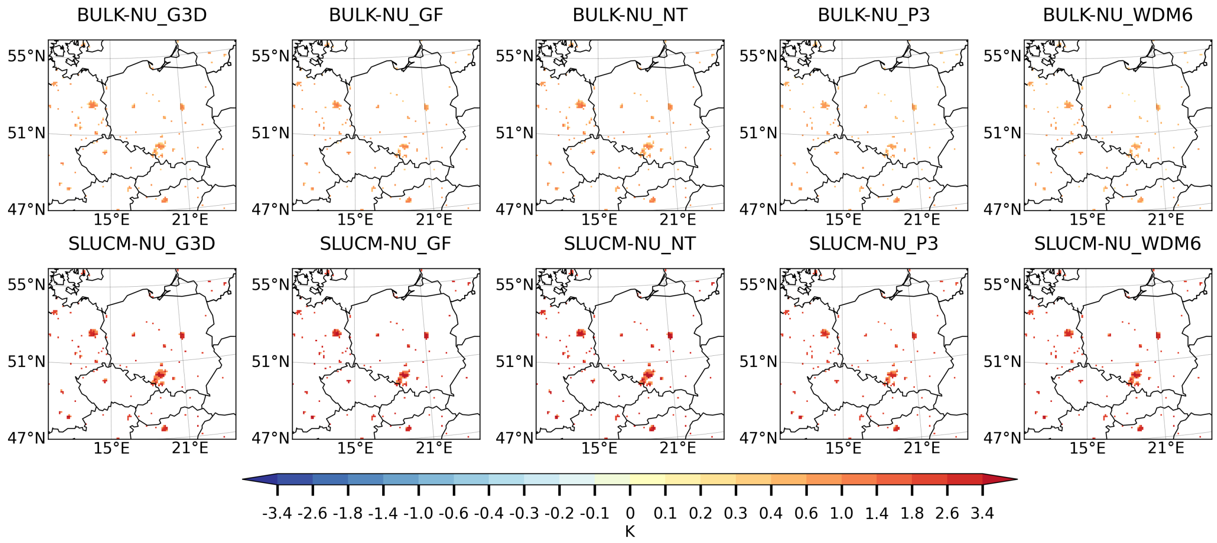

Figure F.1: Spatial differences of multi-year seasonal average of hourly T (in K) between the BULK and no urban scheme (BULK−NU; upper row) simulations and between the SLUCM and no urban scheme (SLUCM−NU; lower row) simulations for the winter season (DJF) over a zoomed-in domain. Colored areas represent statistically significant differences on the 99% level using t-test.

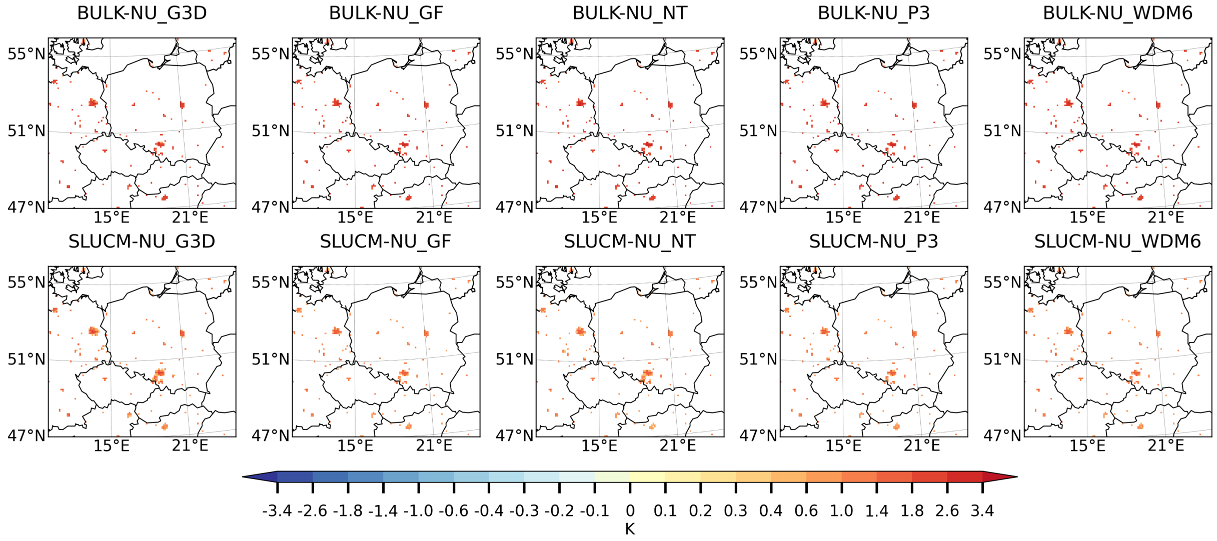

Figure F.2: Same as Fig. F.1 but for the summer season (JJA).

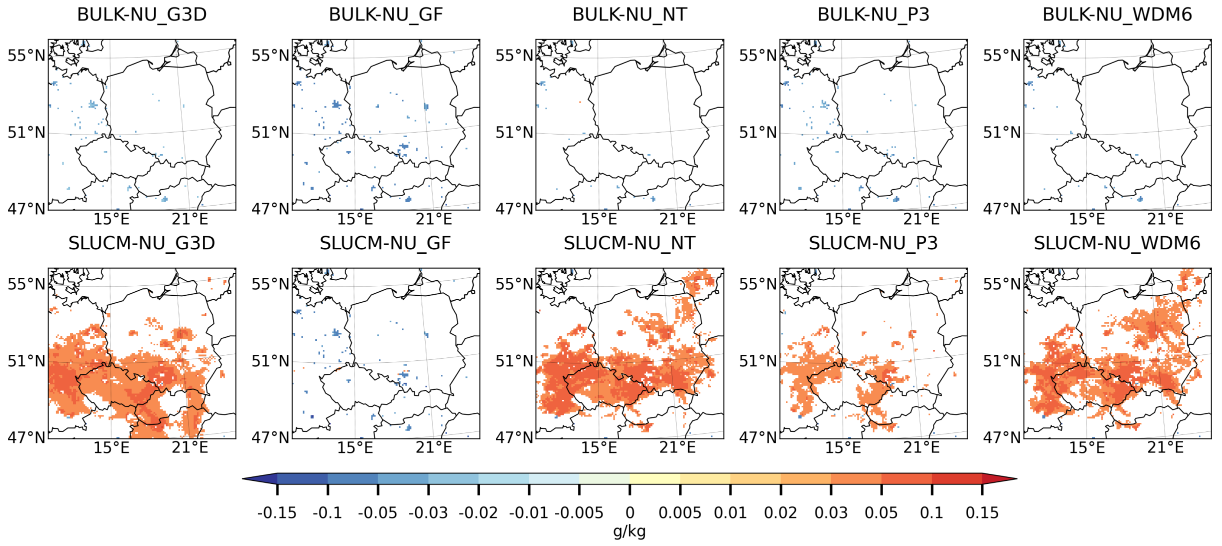

Figure F.3: Spatial differences of multi-year seasonal average of specific humidity (g/kg) between the BULK and no urban scheme (BULK−NU; upper row) simulations and between the SLUCM and no urban scheme (SLUCM−NU; lower row) simulations for the winter season (DJF) over a zoomed-in domain. Colored areas represent statistically significant differences on the 99% level using t-test.

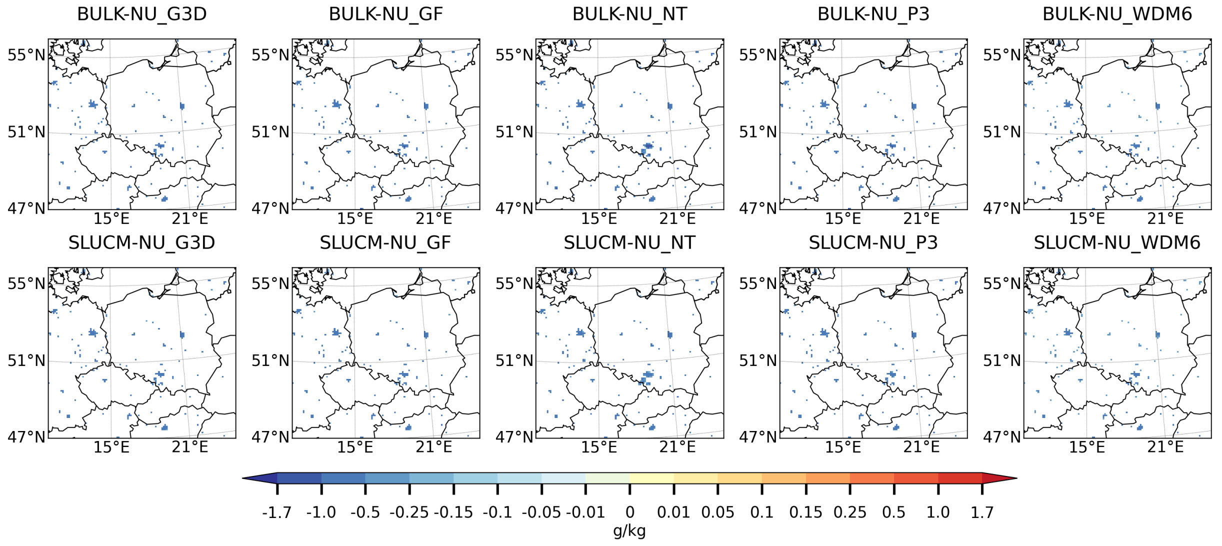

Figure F.4: Same as Fig. F.3 but for the summer season (JJA).

G Diurnal cycles for T2 and Q2 over the selected cities and their rural counterparts

Fig. G.1 presents the differences in simulated temperature between the simulations run with urban schemes and the no-urban simulations. For each city, the panel on the left shows the values for winter, and the right panel shows the values for the summer. For all seasons, the simulated values of temperature in the city centers (solid lines) are higher than those in the vicinity (dotted lines). During the winter months (DJF), simulations run with the SLUCM scheme produce higher values of temperature than the bulk scheme, while the opposite is true for JJA (in line with the spatial distribution of the impact). Two maxima are identified in the differences in the diurnal cycle of temperature over the city centers during DJF when the SLUCM urban scheme is used. These maxima occur in the early morning around 8:00 am and in the afternoon around 5:00 pm. On the other hand, the minimum differences occur around noon. In JJA, there is only one maximum in the evening around 8:00 pm and the minimum differences are shifted towards the early morning around 8:00 am. Differences between the bulk and no-urban schemes follow a similar pattern but lower values and less prominent maxima. Differences in DJF between simulations run with the SLUCM and the bulk schemes are bigger than 1 °C for the majority of the cities, while in JJA the differences are smaller and the simulations run with the SLUCM scheme show lower values than those with the bulk scheme. Compared with the values in the vicinity, the differences around the city centers are higher in DJF than in JJA for simulations run with the SLUCM scheme.

The combinations producing the highest differences in DJF are the SLUCM urban scheme with the NT convection scheme reaching up to 2.8 °C for some cities, followed by the SLUCM urban scheme with the WDM6 microphysics scheme. In JJA, the bulk scheme with the P3 microphysics scheme or with the WDM6 microphysics schemes are the combinations that produce the highest values. The size of these differences depends on the city. For example, the highest difference in Budapest reaches up to 3 °C in JJA, while Munich shows the lowest (maximum values at around 1 °C) for the same period.

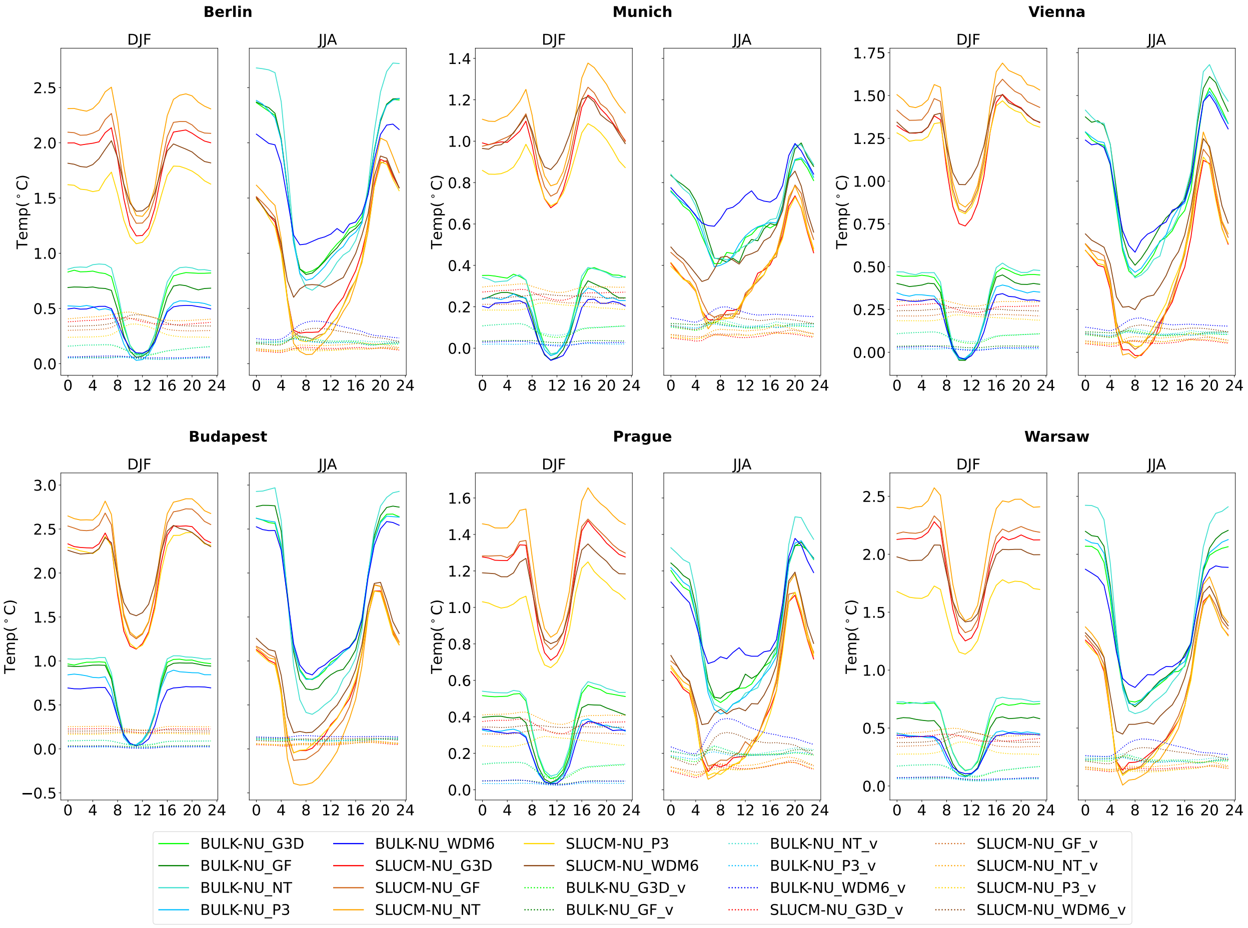

Figure G.1: Diurnal cycle of city center (solid lines) and vicinity (dotted lines) for BULK−no urban (green and blue lines) and SLUCM−no urban (red and brown lines) for temperature at 2 m (°C). Differences between BULK−NU using the G3D convection scheme are shown in light green lines; BULK−NU with GF, in dark green lines; BULK−NU with NT, in turquoise lines; BULK−NU with the P3 microphysics scheme, in light blue lines; and BULK−NU with WDM6, in dark blue lines. In case of SLUCM−NU differences, red lines show the differences when the G3D convection scheme is used; SLUCM−NU with GF are shown in light brown; SLUCM−NU with NT, in orange; SLUCM−NU with P3, in yellow; and SLUCM−NU with WDM6, in dark brown. For each of the six selected cities, the panel on the left shows results for DJF and the panel on the right, results for JJA.

The variation of specific humidity is higher in the city centers than in the vicinities, especially during JJA (Fig. G.2). The values of specific humidity in the city center show a significant minimum around noon in DJF (up to 0.1 g kg^{-1}) and between 8:00 am and 6:00 pm in JJA (at around 0.8 g kg^{-1} in Budapest). A slight increase in specific humidity is detected during the nighttime (up to 0.1 g kg^{-1} for some cities such as Warsaw). In general, higher values of specific humidity are obtained with the SLUCM urban scheme in

DJF, and with the bulk scheme in JJA.

The values of specific humidity decrease during the daytime and increase during the nighttime for both seasons. However, the reduction in specific humidity is more pronounced in the cities than in their vicinities and during JJA. This reduction can be explained due to higher vertical turbulent mixing, less water available, and stronger convection over cities during the daytime and in summer. Zhou et al., 2024[?] analyzed the impact of urban expansion on temperature and specific humidity over the Yangtze River Delta, China, drying during two heatwaves. The authors showed that this expansion produced notable near-surface warming and a pronounced reduction of moisture especially during the daytime, which agrees with the results presented in this manuscript. In general, the combination SLUCM and WDM6 scheme lead to the lowest reductions in the cities (in the vicinity it is the combination bulk and WDM6 schemes), while the highest reductions are achieved with the bulk schemes. Differences between the city center and its surroundings are city-dependant. For example, it is around $0.7 \cdot 10^{-3}$ kg/kg in Berlin and around $0.3 \cdot 10^{-3}$ kg/kg in Prague in summer, which means a 80% difference for the two cities.

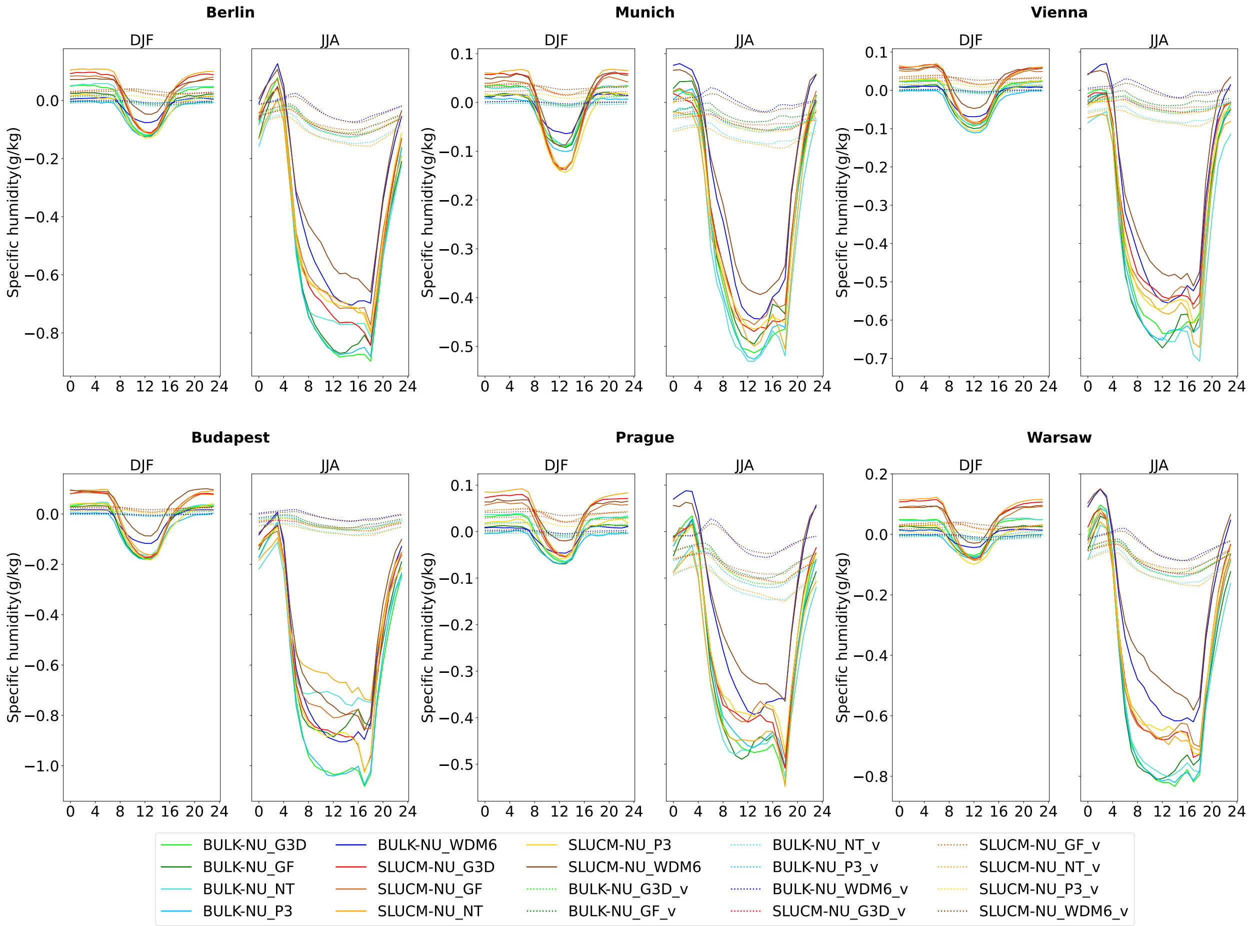

Figure G.2: Diurnal cycle of city center (solid lines) and vicinity (dotted lines) for BULK−no urban (green and blue lines) and SLUCM−no urban (red and brown lines) for specific humidity (g/kg). Differences between BULK−NU using the G3D convection scheme are shown in light green lines; BULK−NU with GF, in dark green lines; BULK−NU with NT, in turquoise lines; BULK−NU with the P3 microphysics scheme, in light blue lines; and BULK−NU with WDM6, in dark blue lines. In case of SLUCM−NU differences, red lines show the differences when the G3D convection scheme is used; SLUCM−NU with GF are shown in light brown; SLUCM−NU with NT, in orange; SLUCM−NU with P3, in yellow; and SLUCM−NU with WDM6, in dark brown. For each of the six selected cities, the panel on the left shows results for DJF and the panel on the right, results for JJA.
