# Supplementary figures and images for: Long‐Term Impact of Urban Areas on Meteorological Conditions Over Central Europe
Source: Ann N Y Acad Sci. 2025 Sep 28;1553(1):461–76. doi: 10.1111/nyas.70069 (PMC12645272; doi:10.1111/nyas.70069)

**Berlin****Munich****Vienna**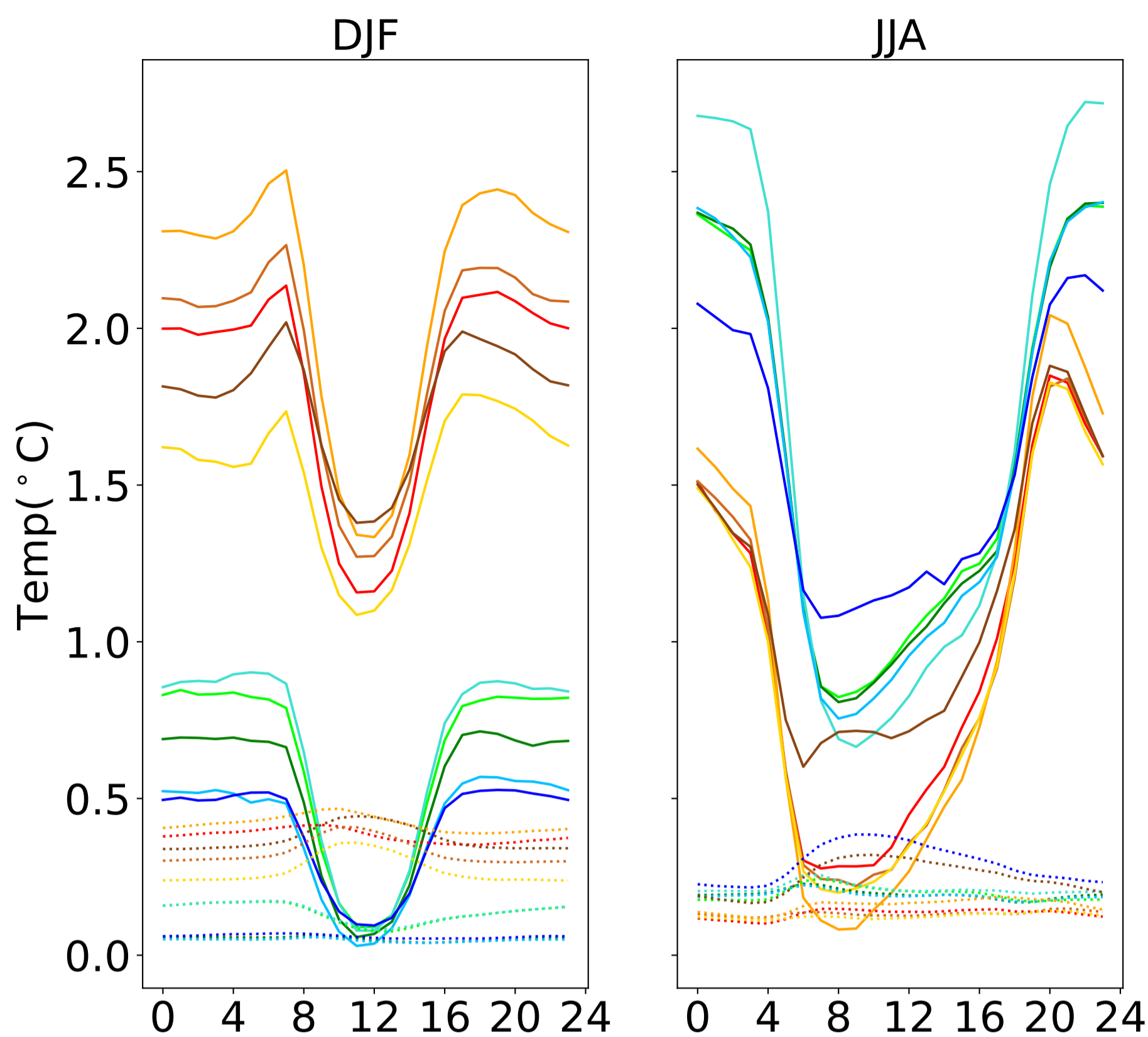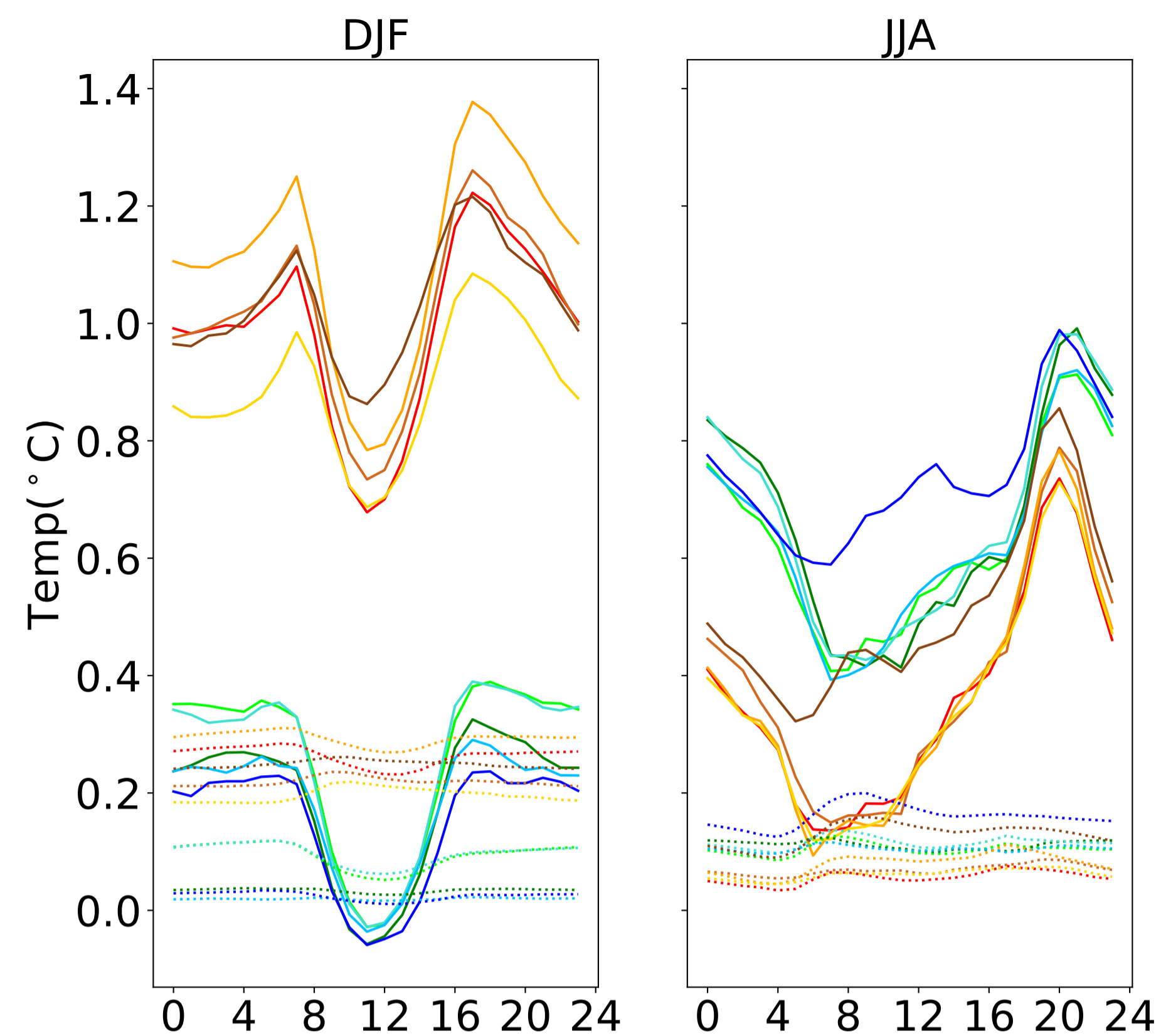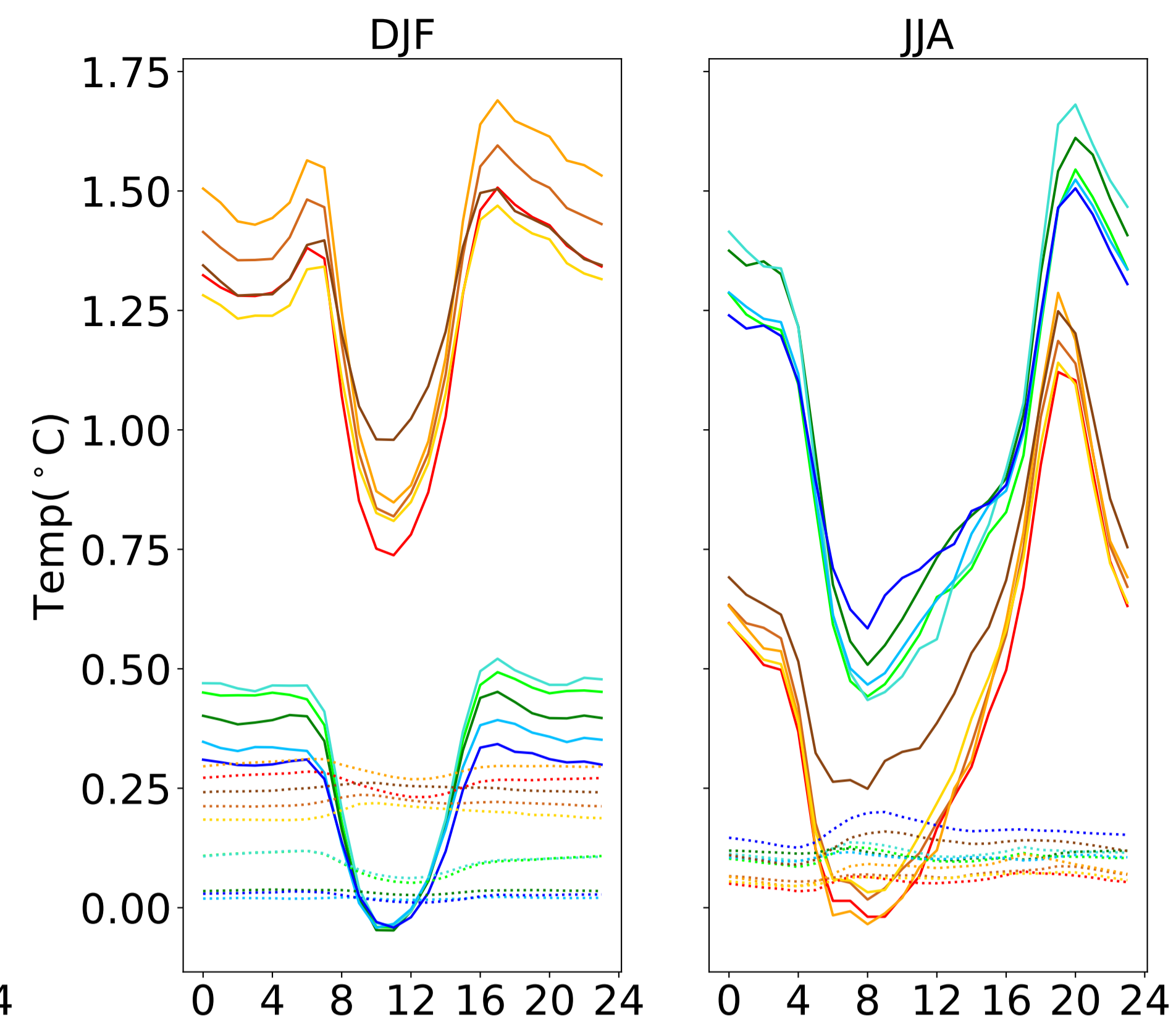**Budapest****Prague****Warsaw**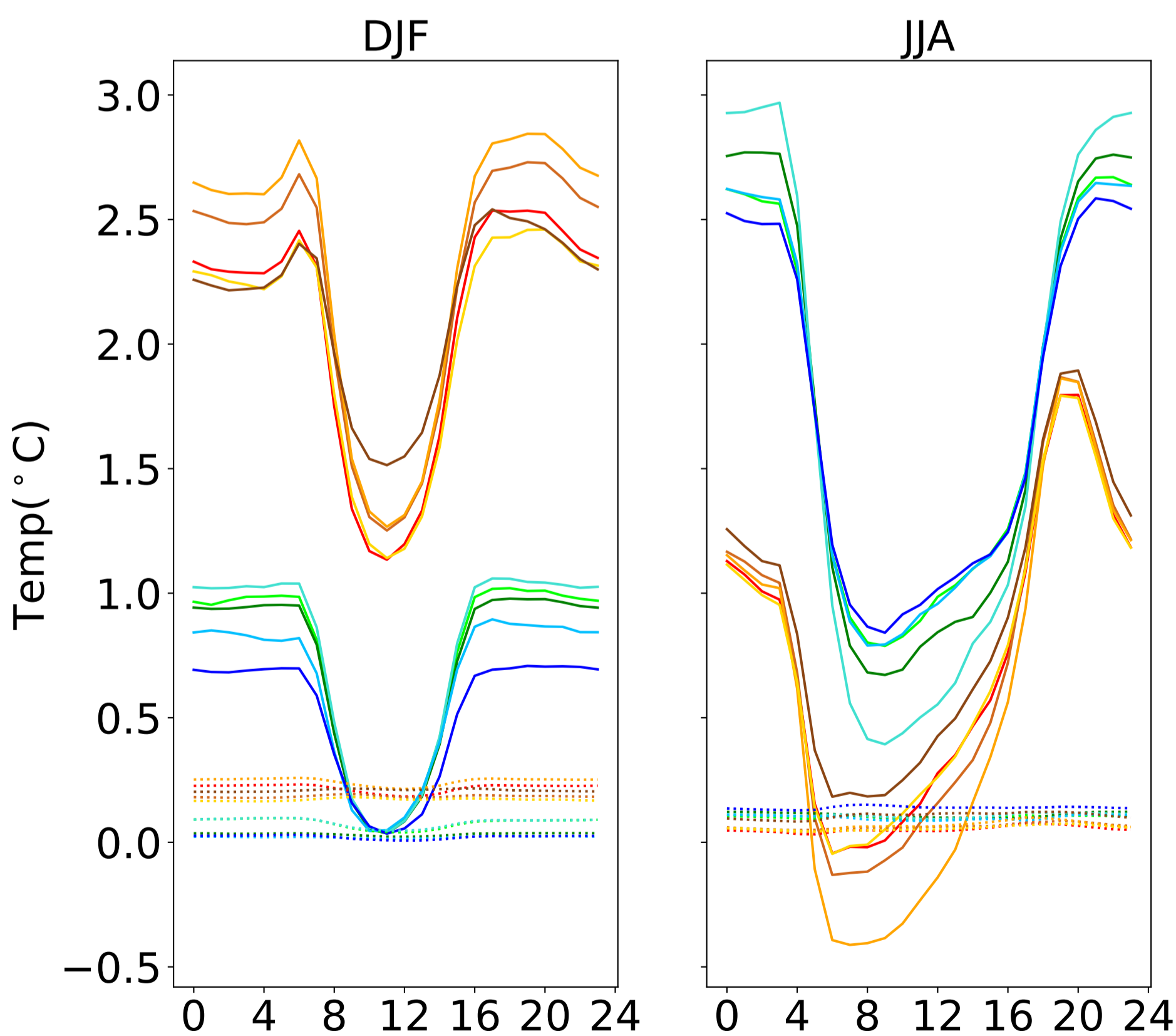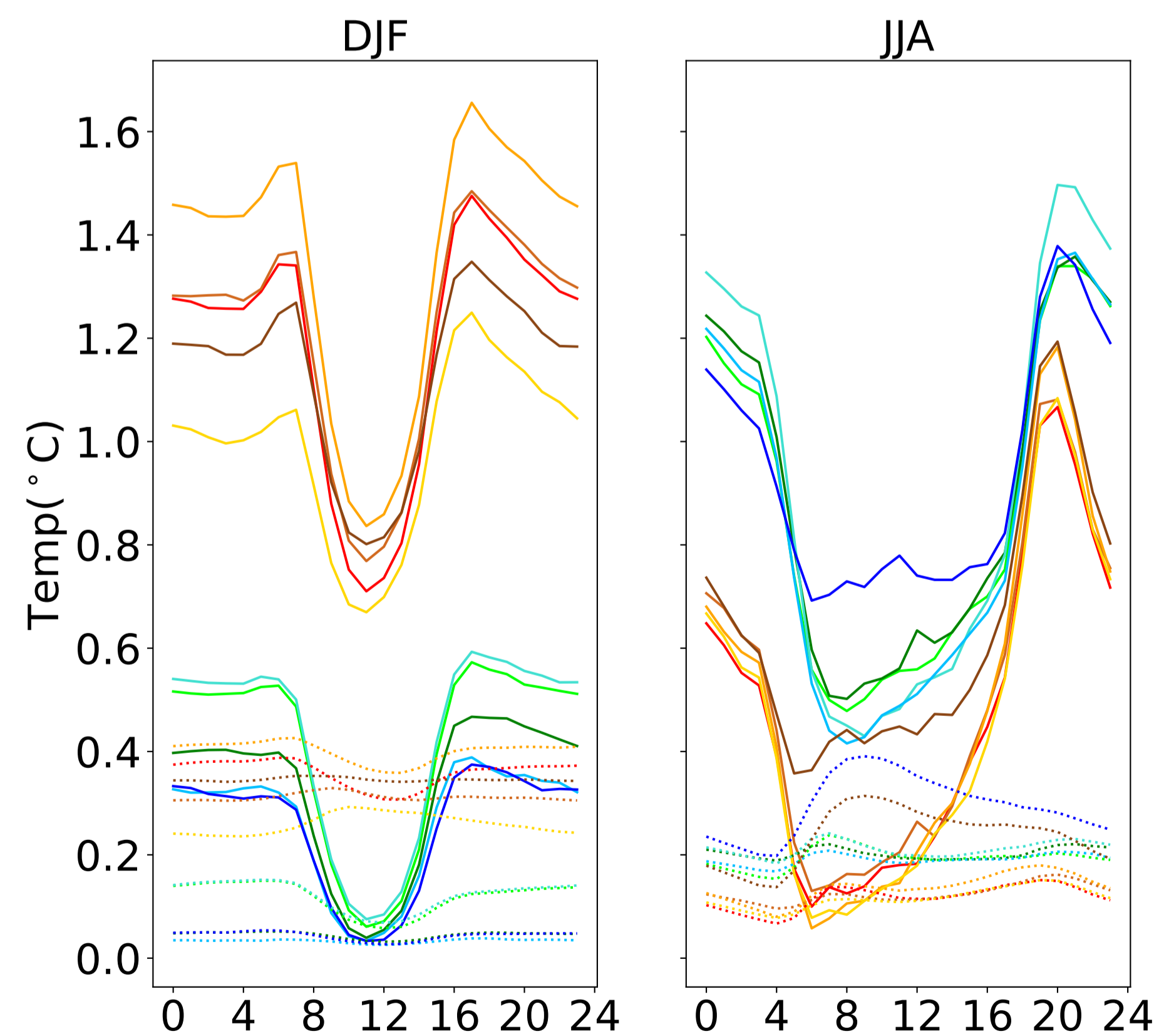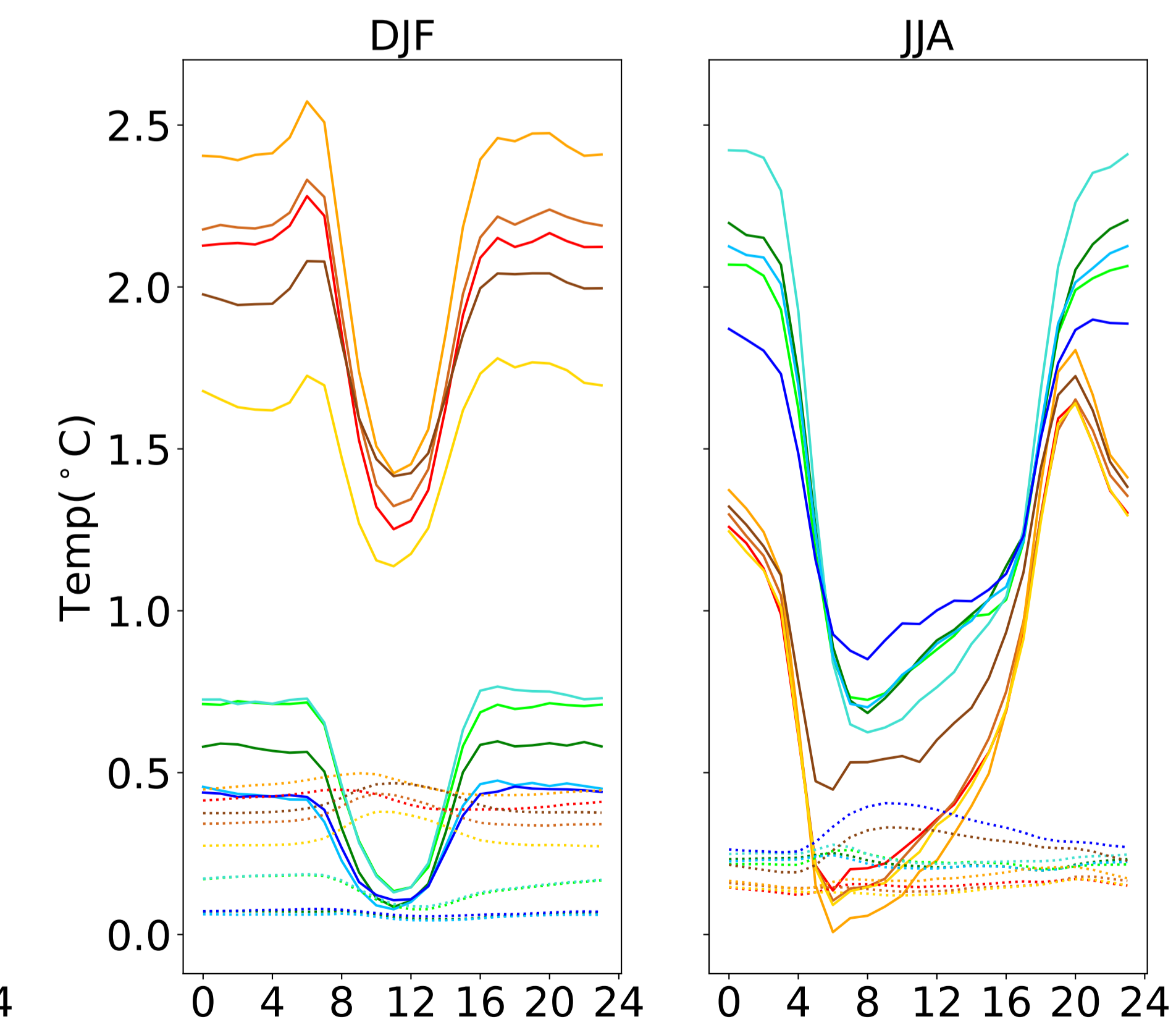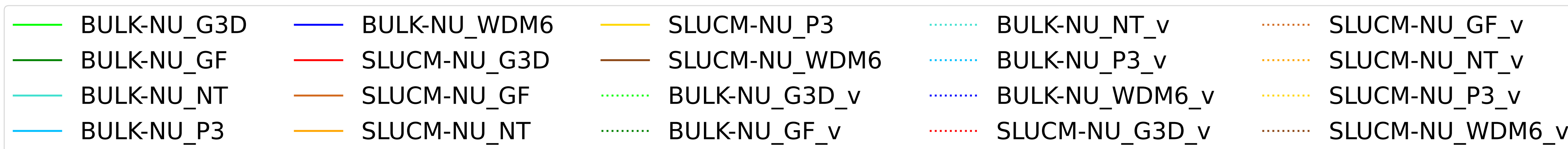

Supplement: Supplementary file 2 — Data S1 [file NYAS-1553-461-s001.zip › Diff_center_and_vic_T2.pdf]

Berlin

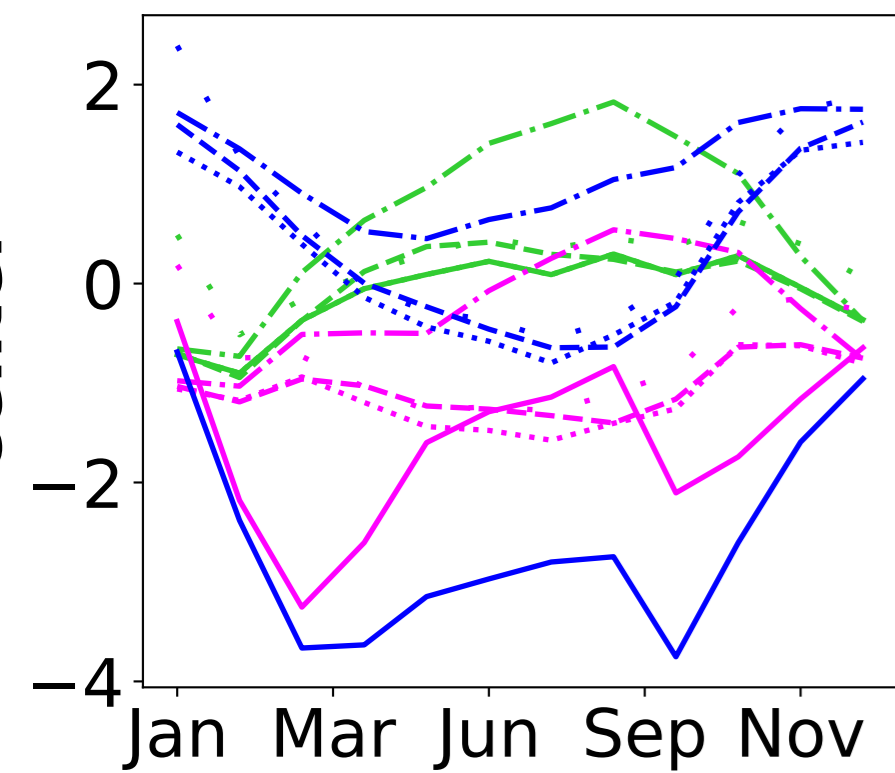

Budapest

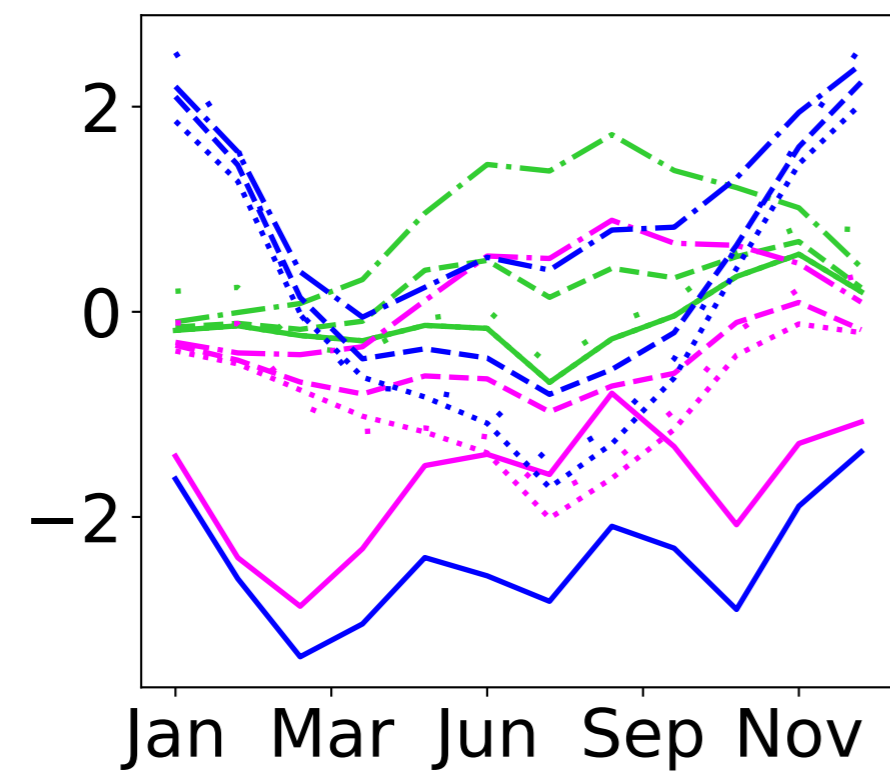

Munich

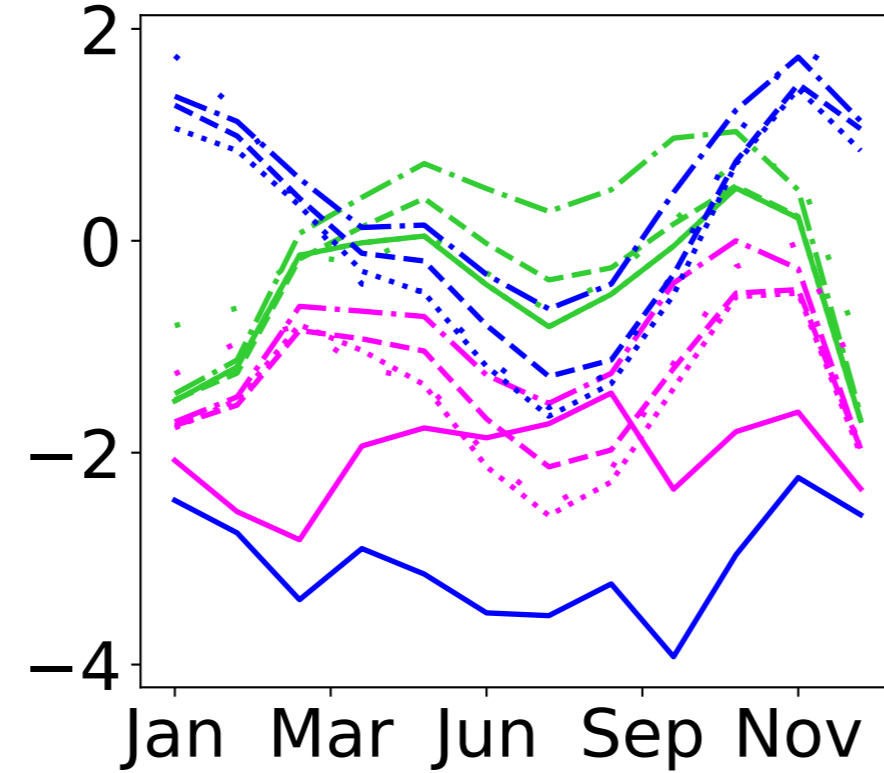

Prague

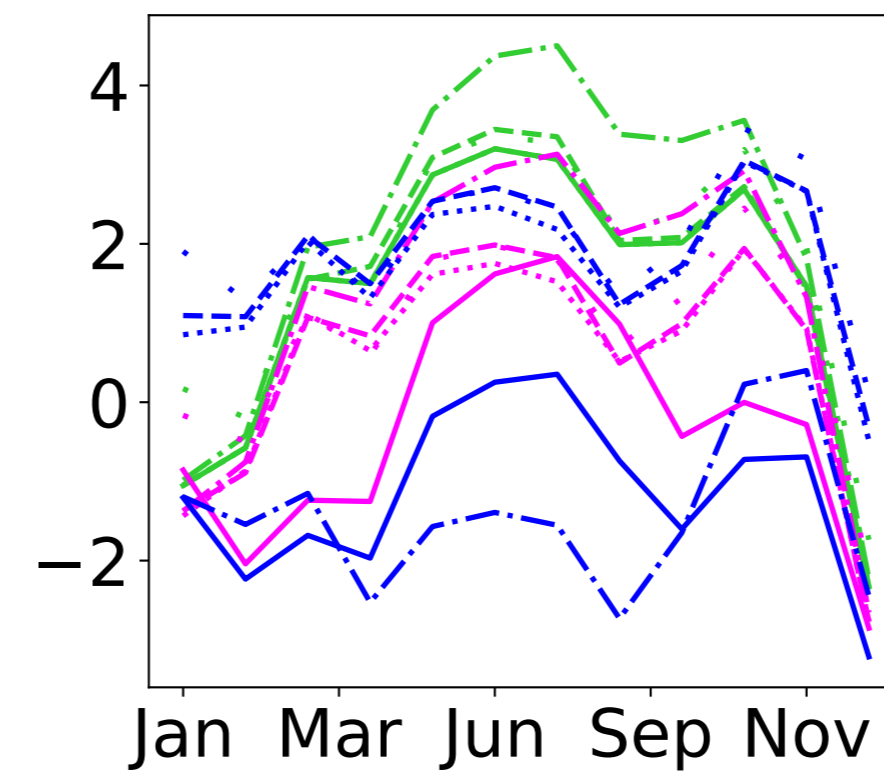

Vienna

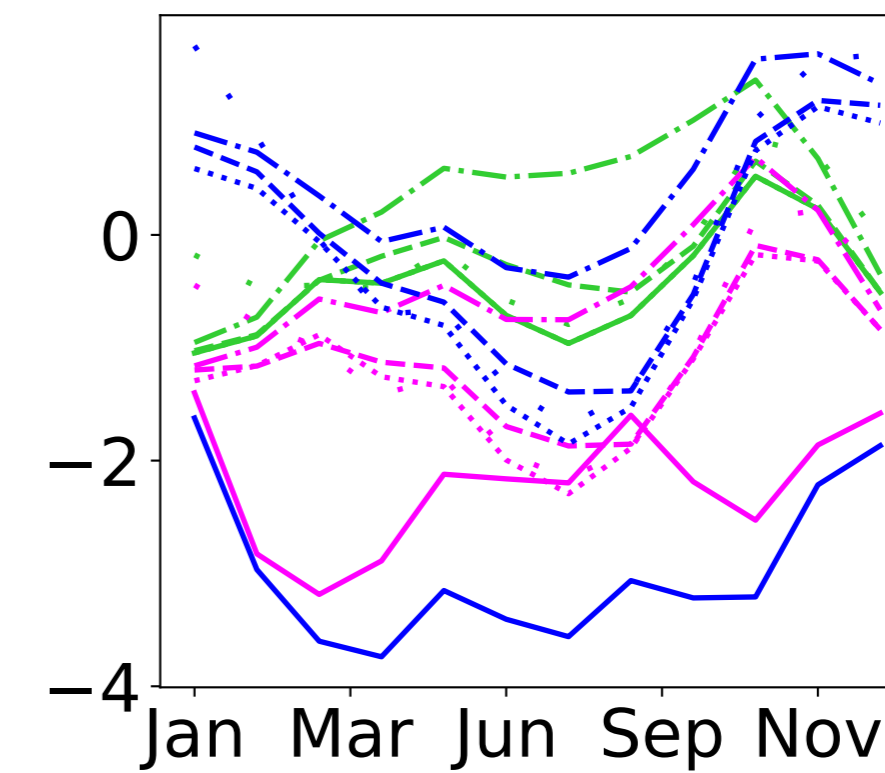

Warsaw

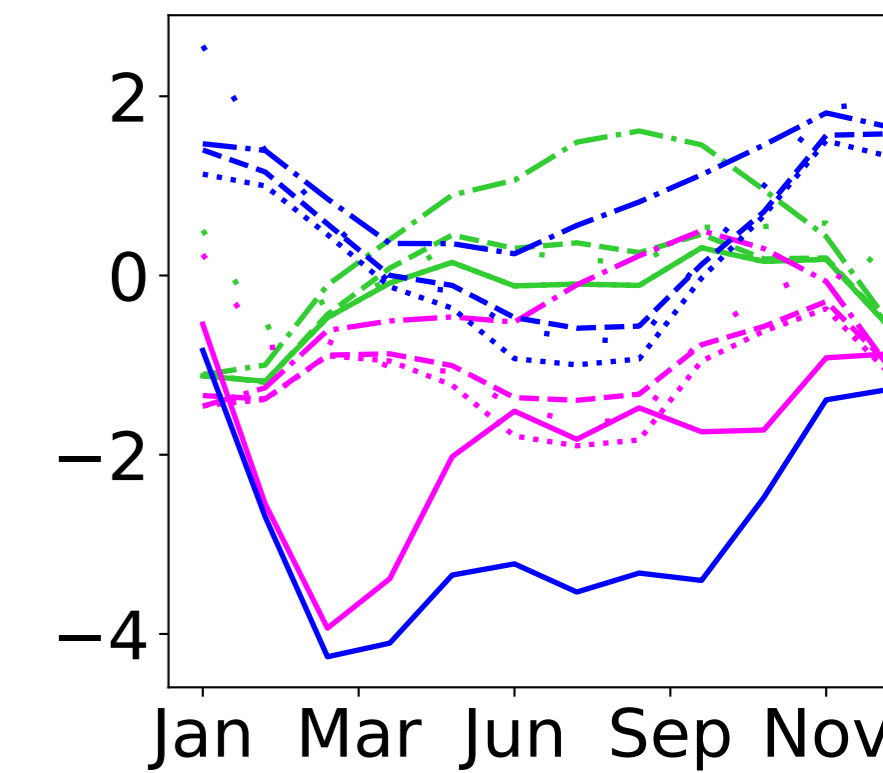

Center

Vicinity

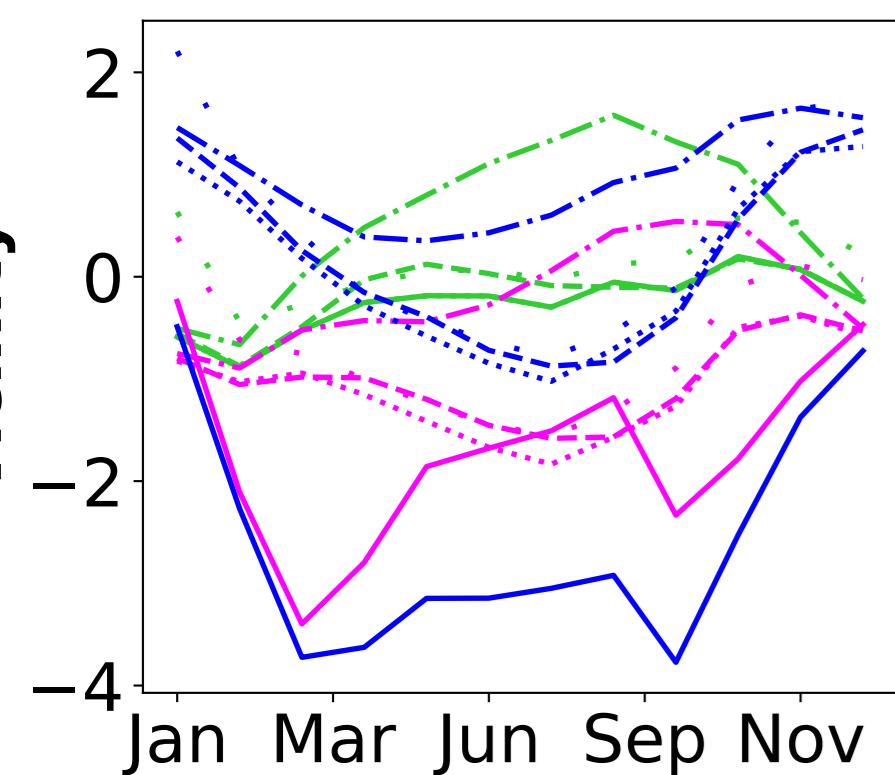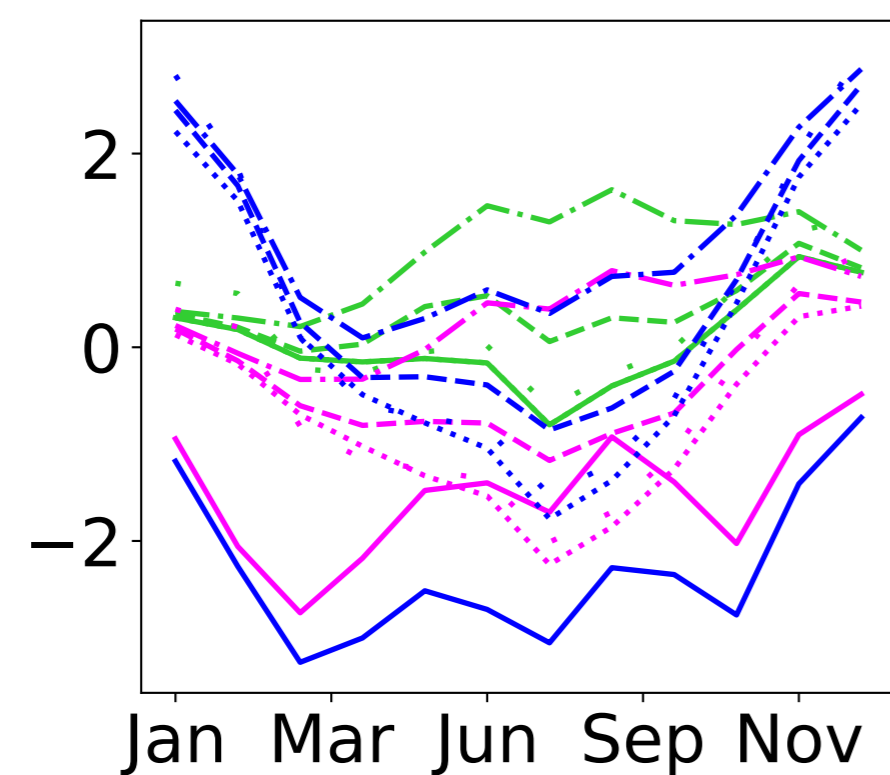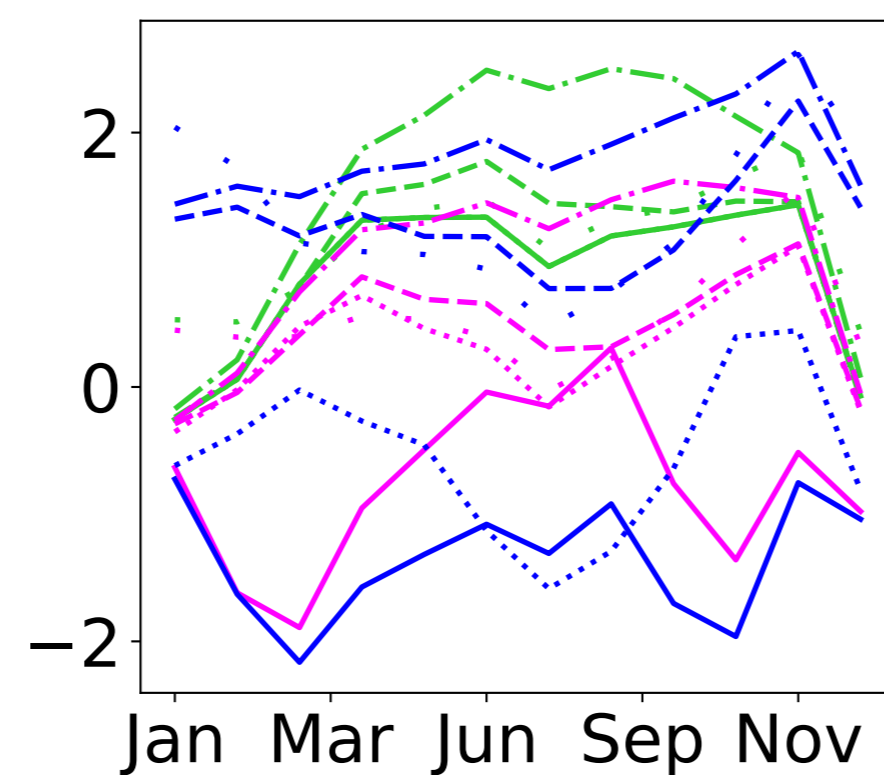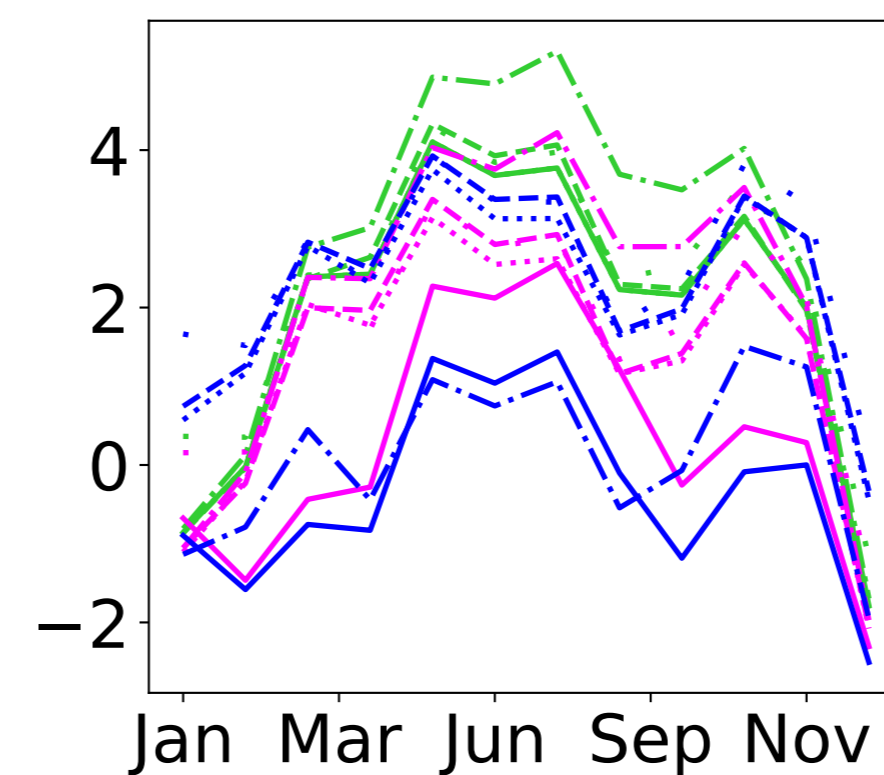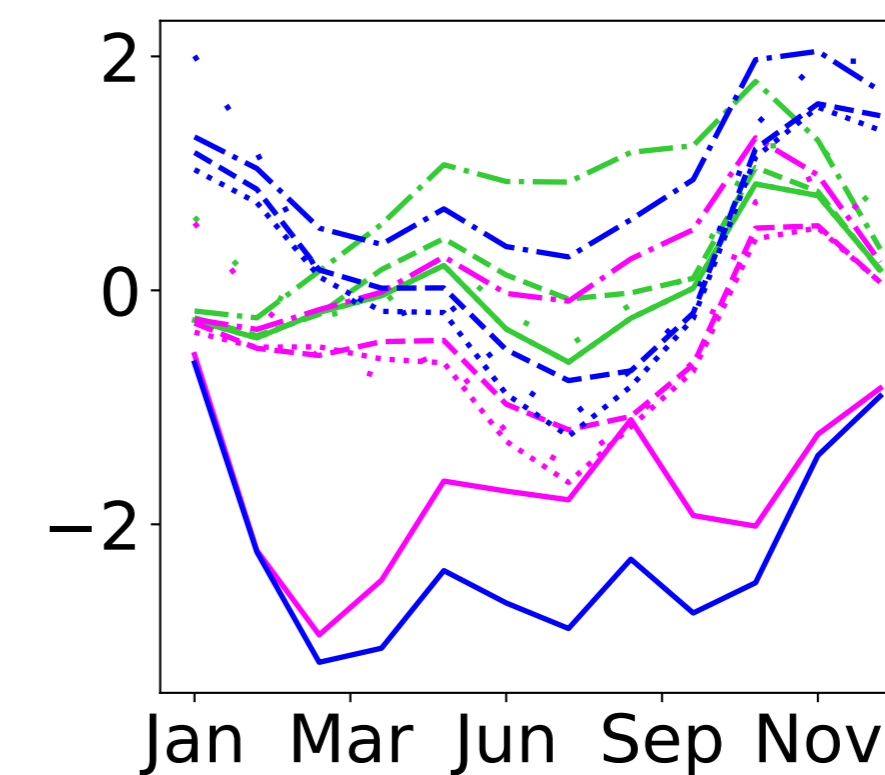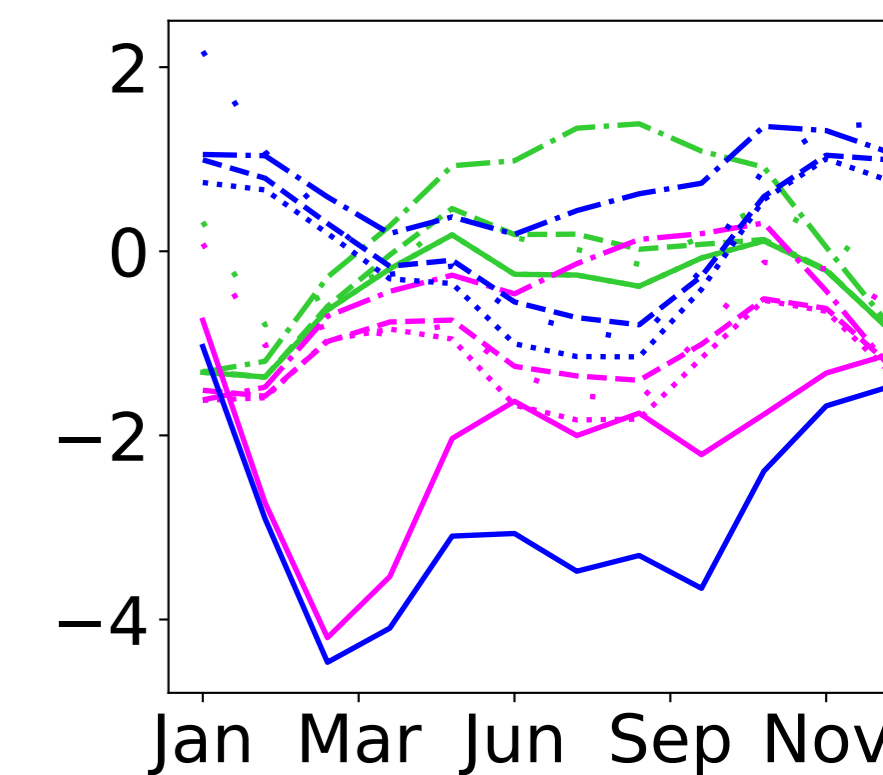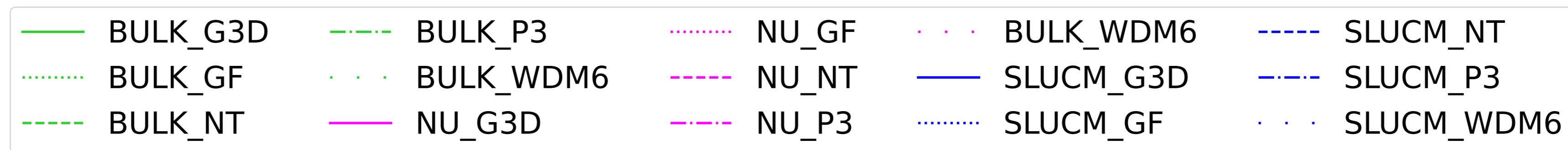

Supplement: Supplementary file 2 — Data S1 [file NYAS-1553-461-s001.zip › Comparison_obs_sims_T2max.pdf]

**Berlin**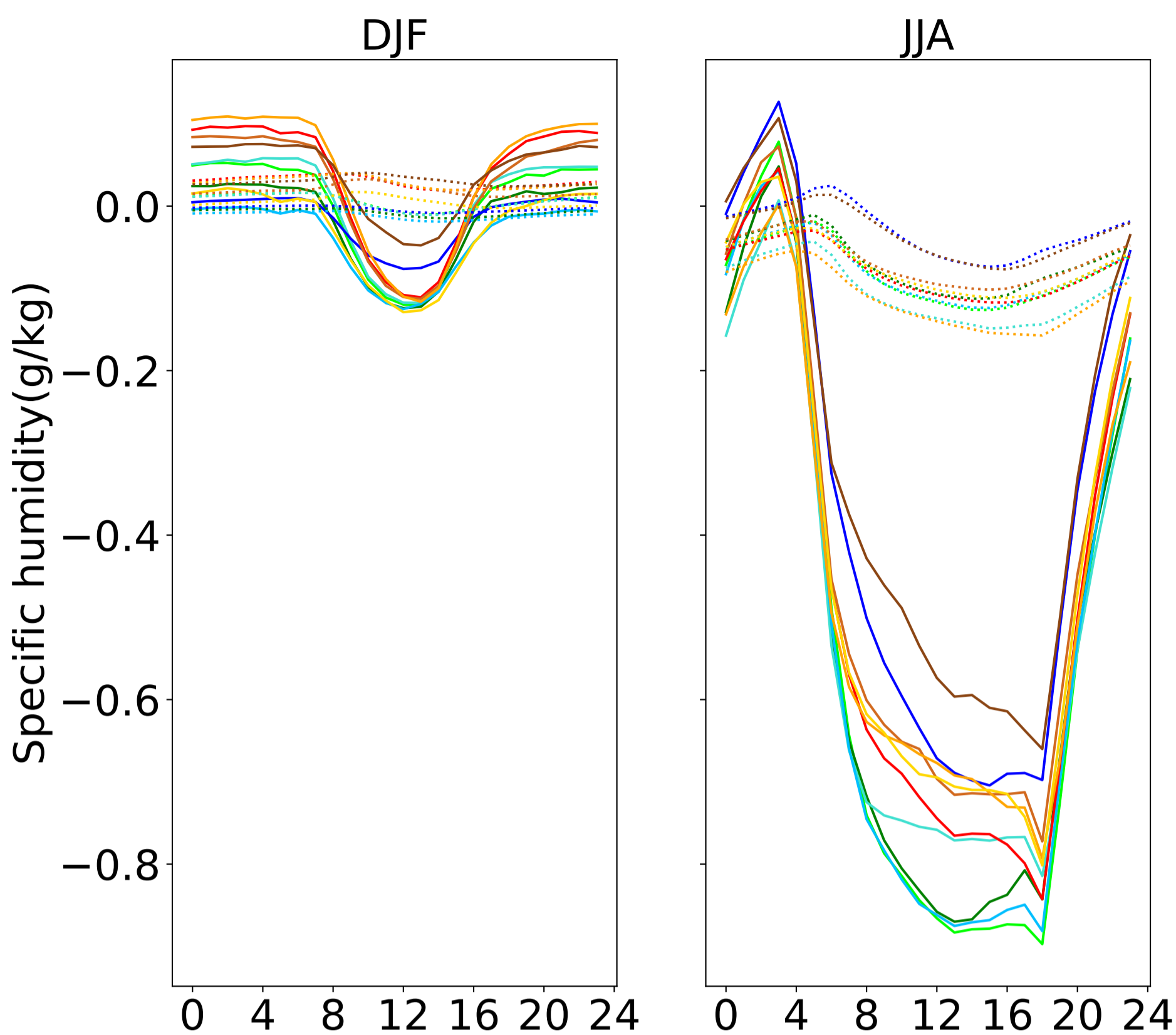**Munich**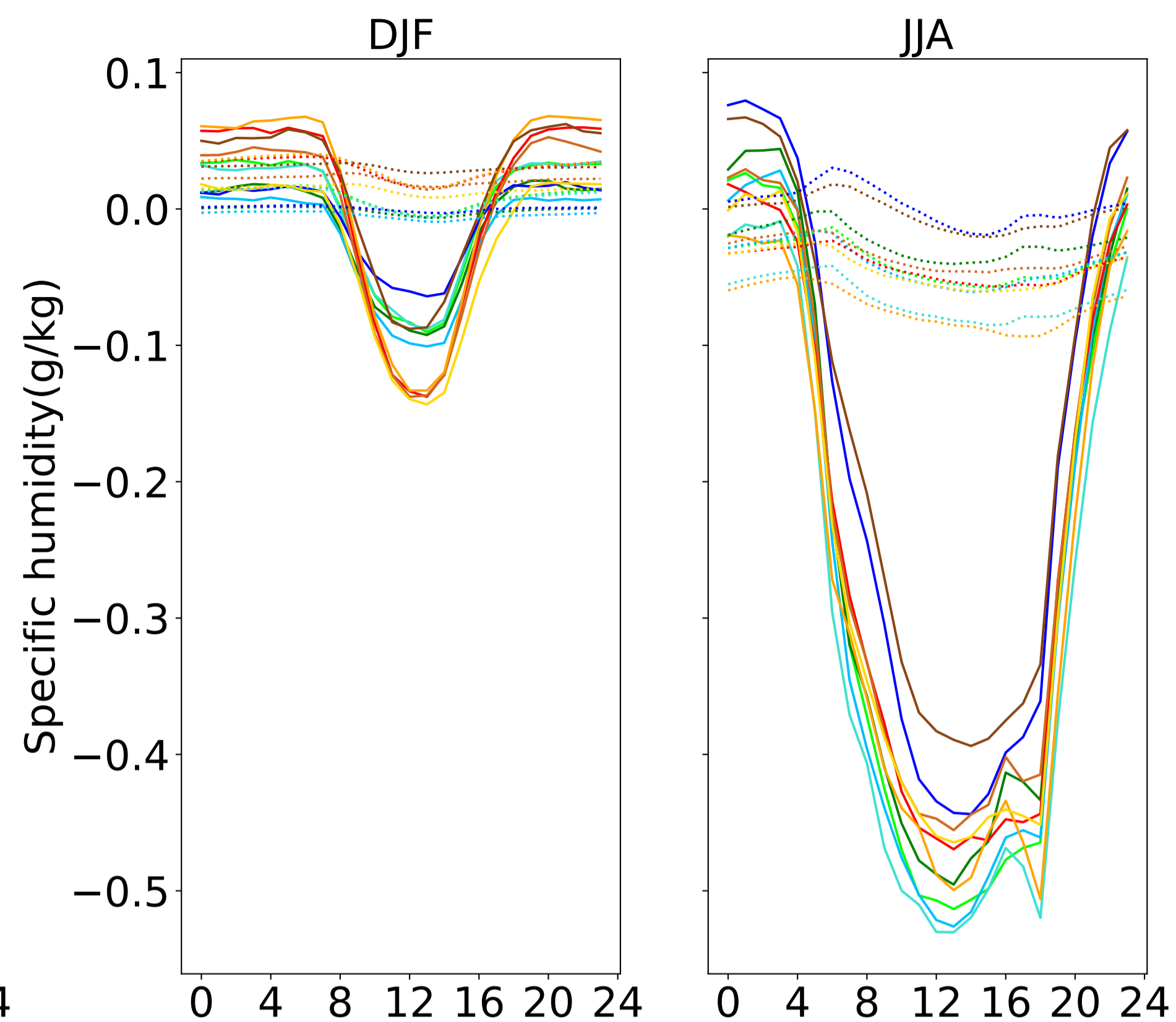**Vienna**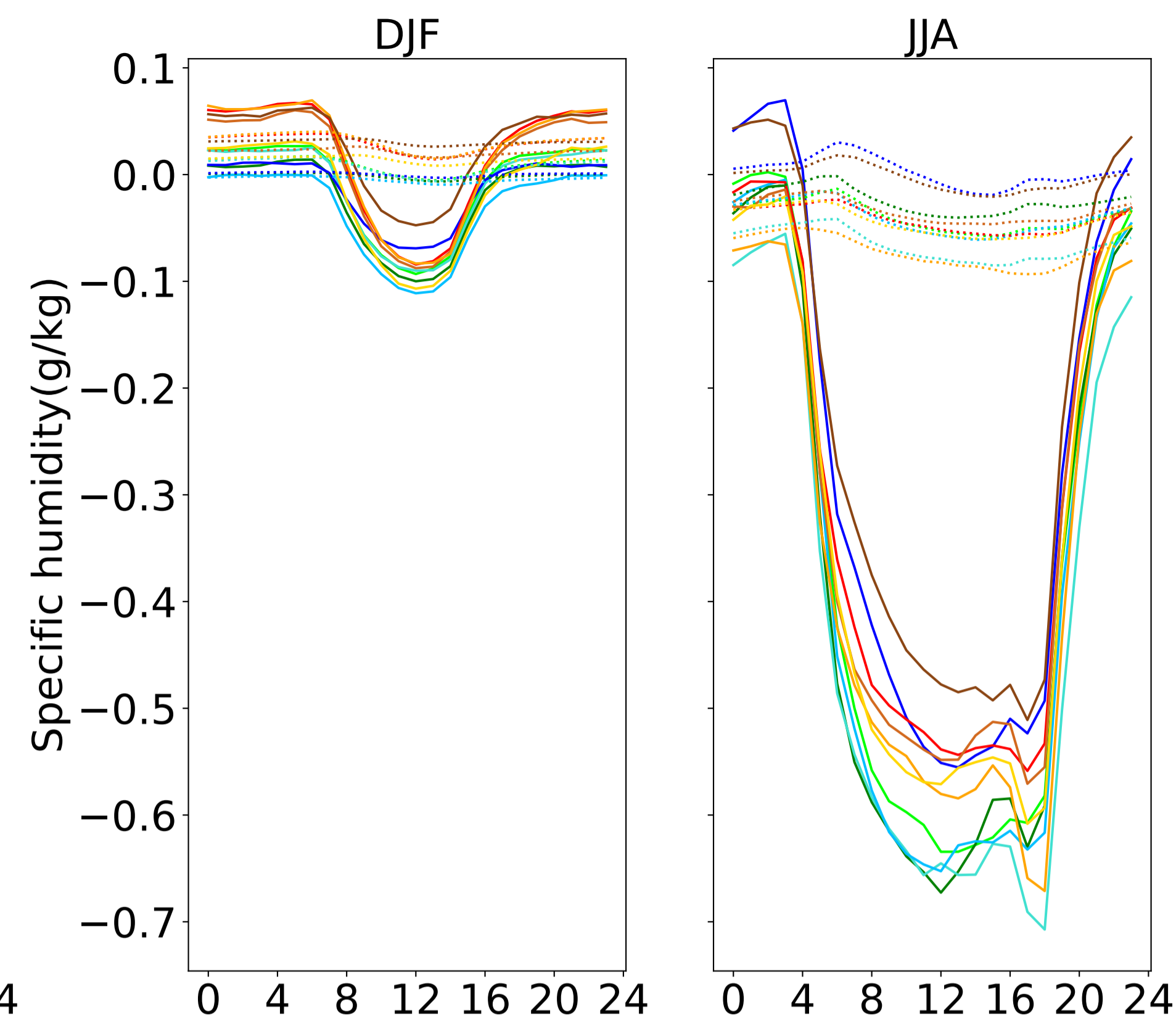**Budapest**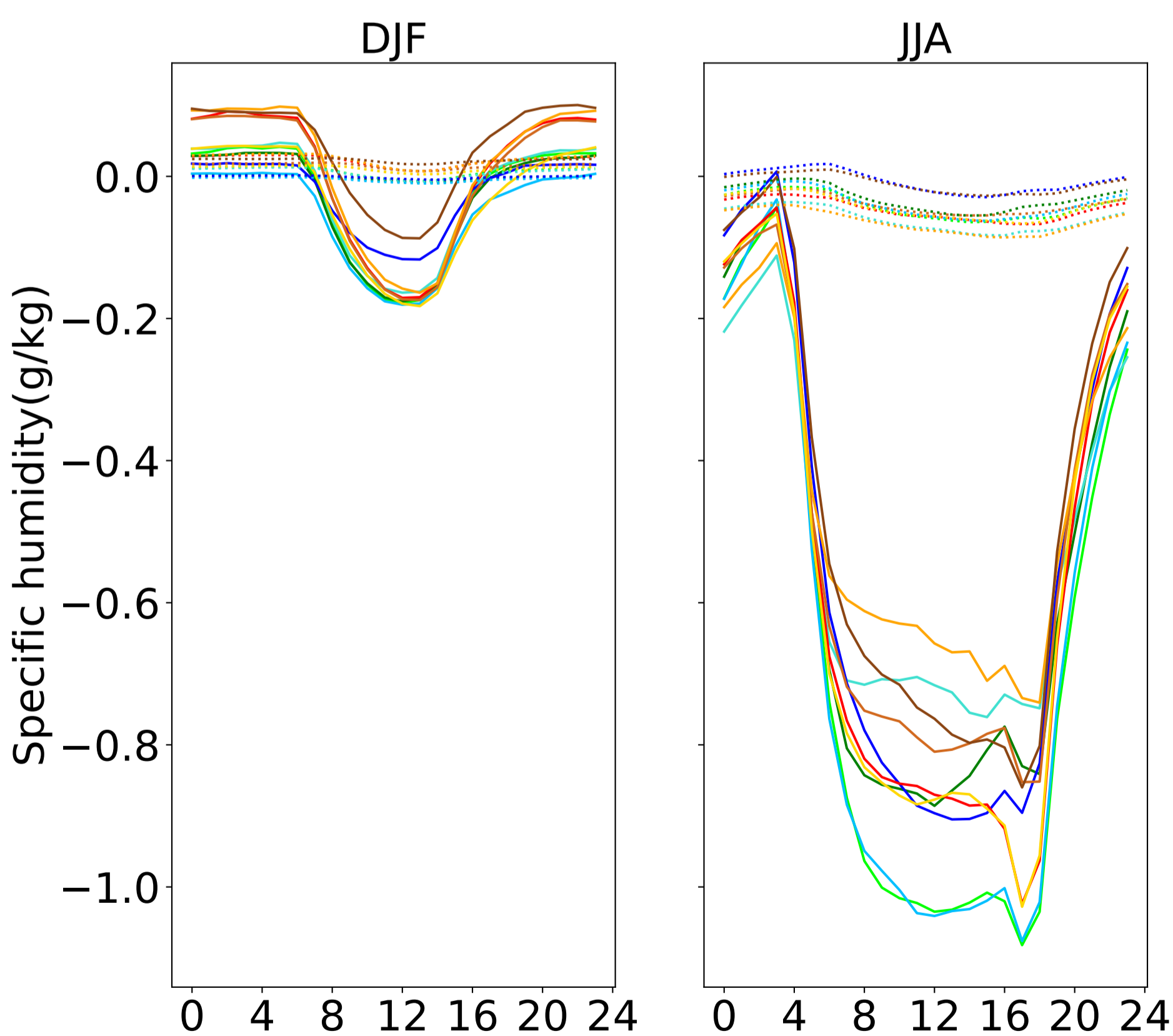**Prague**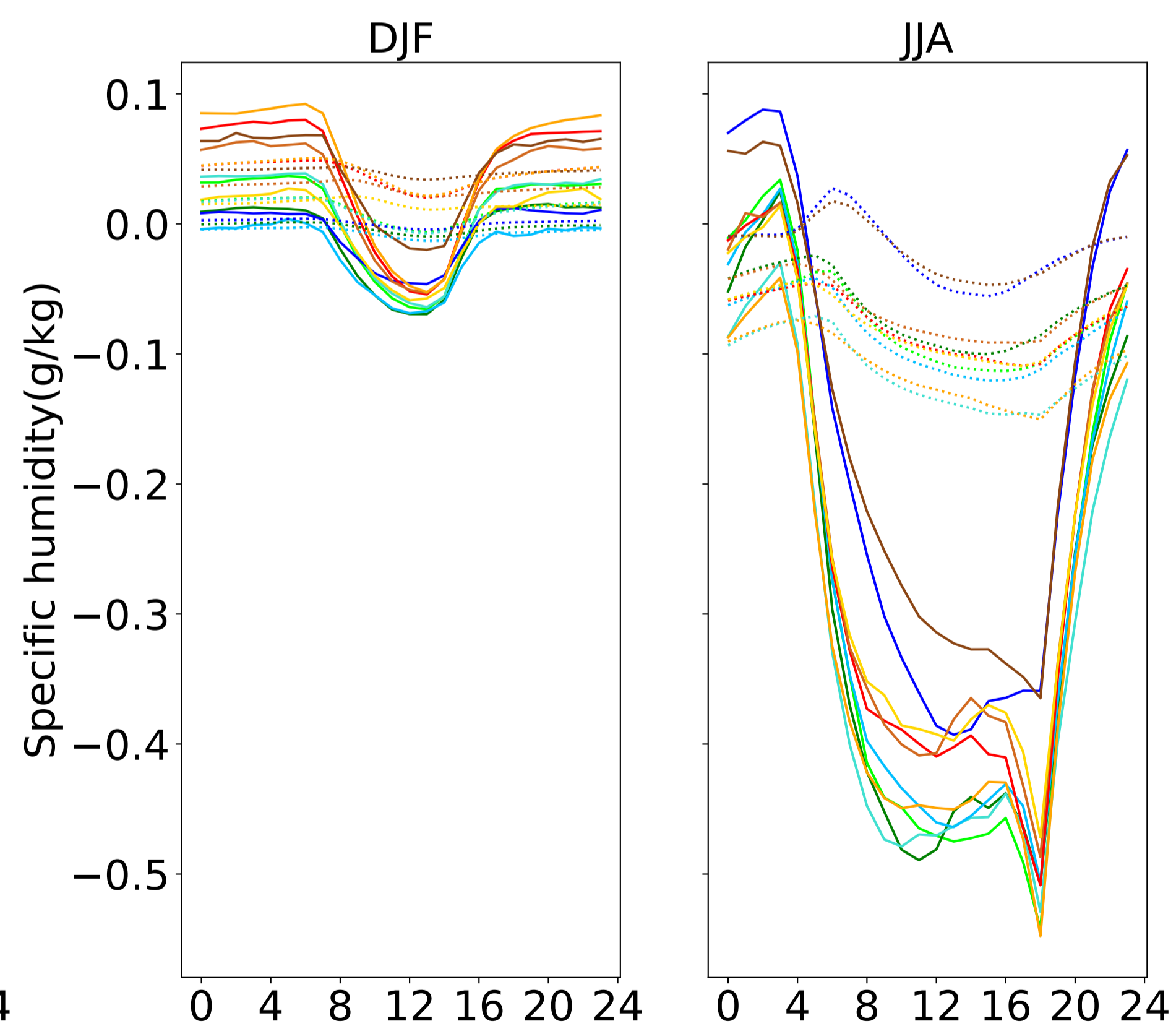**Warsaw**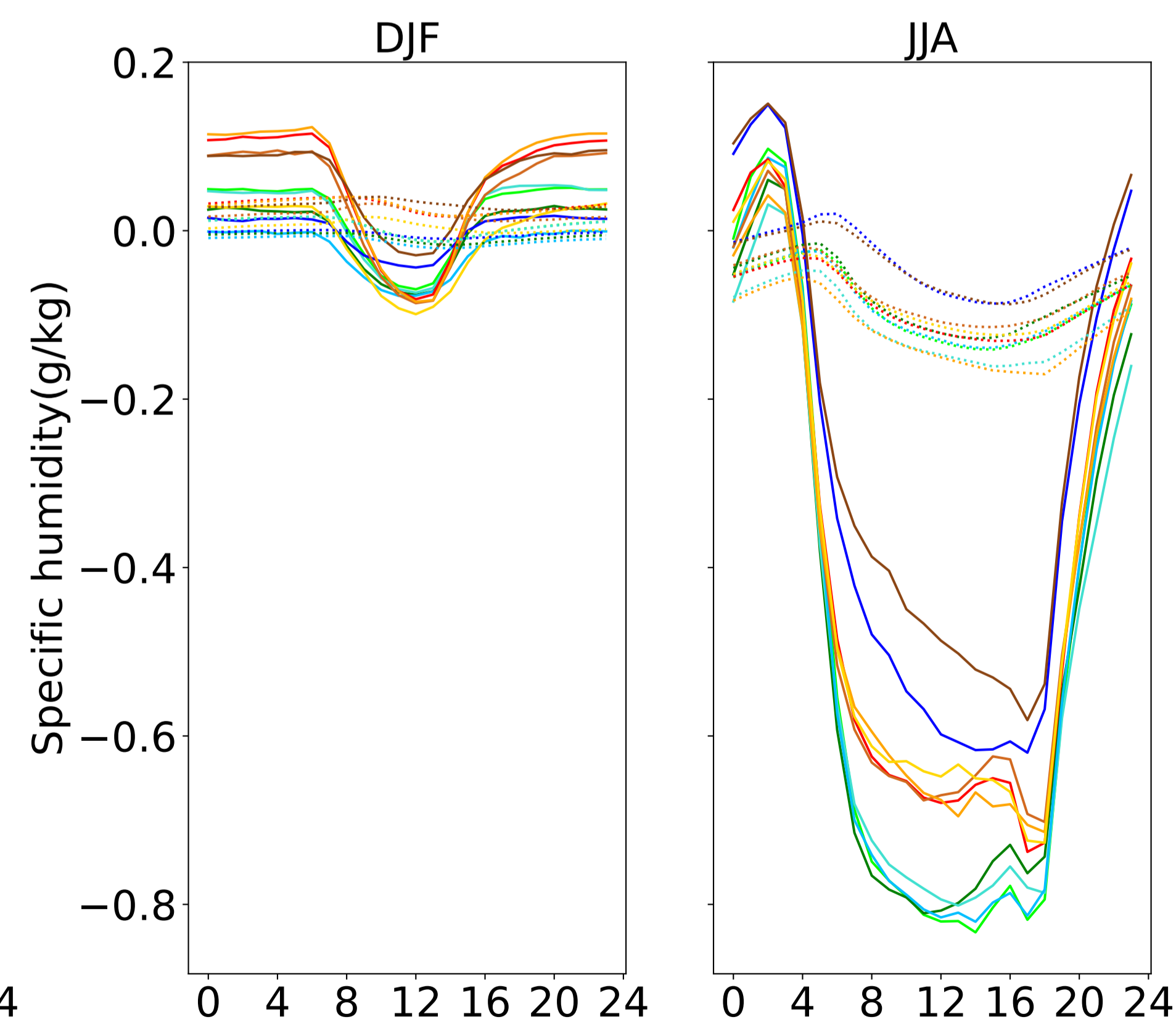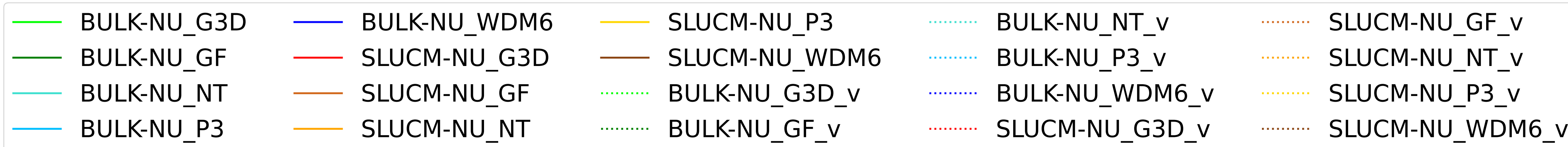

Supplement: Supplementary file 2 — Data S1 [file NYAS-1553-461-s001.zip › Diff_center_and_vic_Q2.pdf]

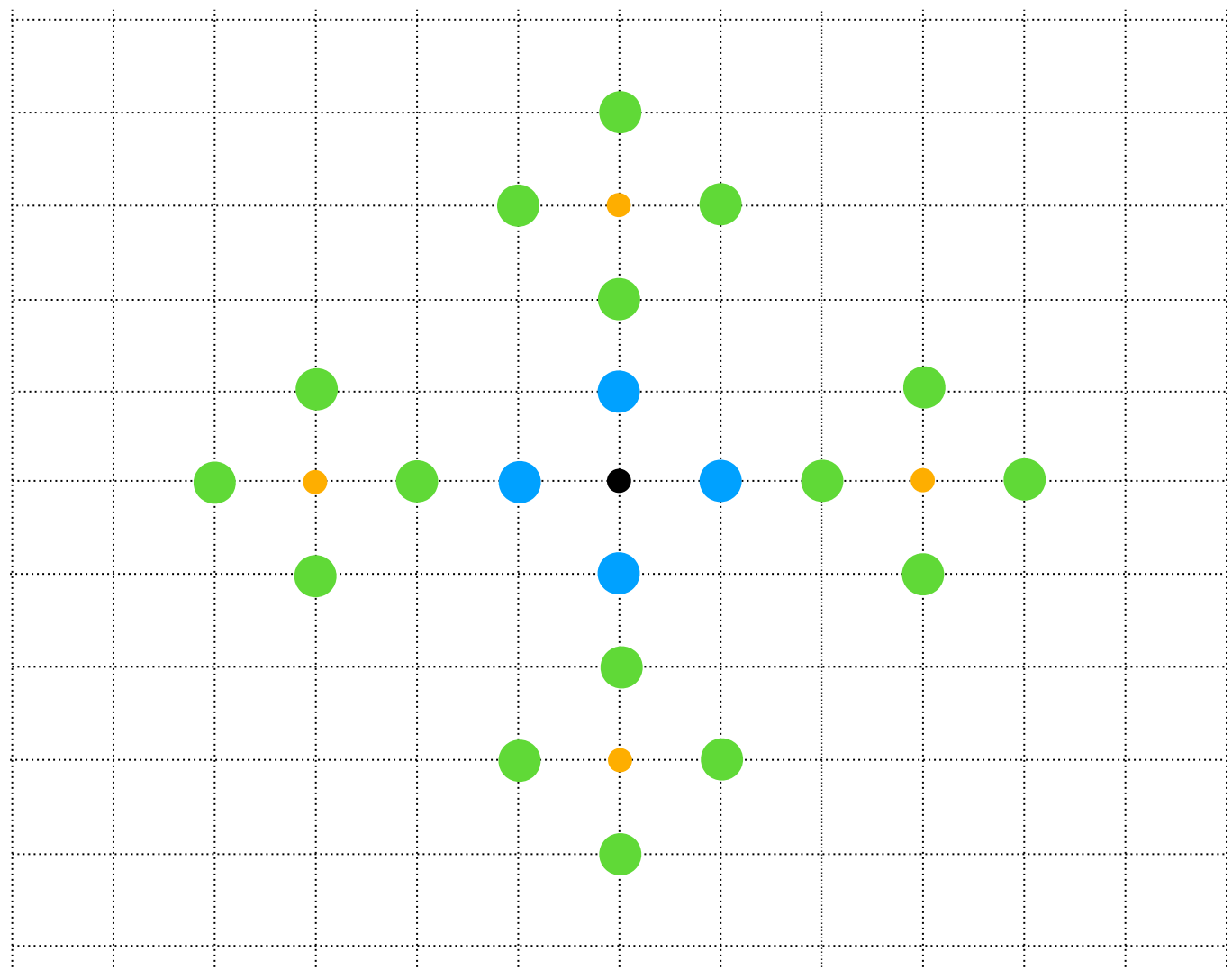

Supplement: Supplementary file 2 — Data S1 [file NYAS-1553-461-s001.zip › grid_urban_rural.pdf]

Berlin

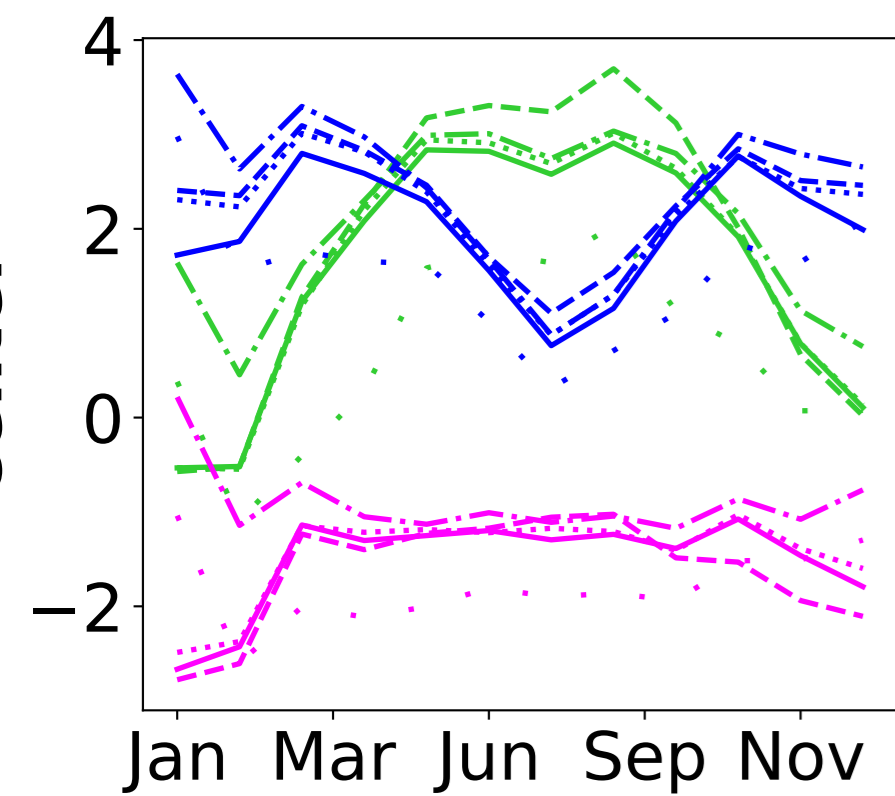

Budapest

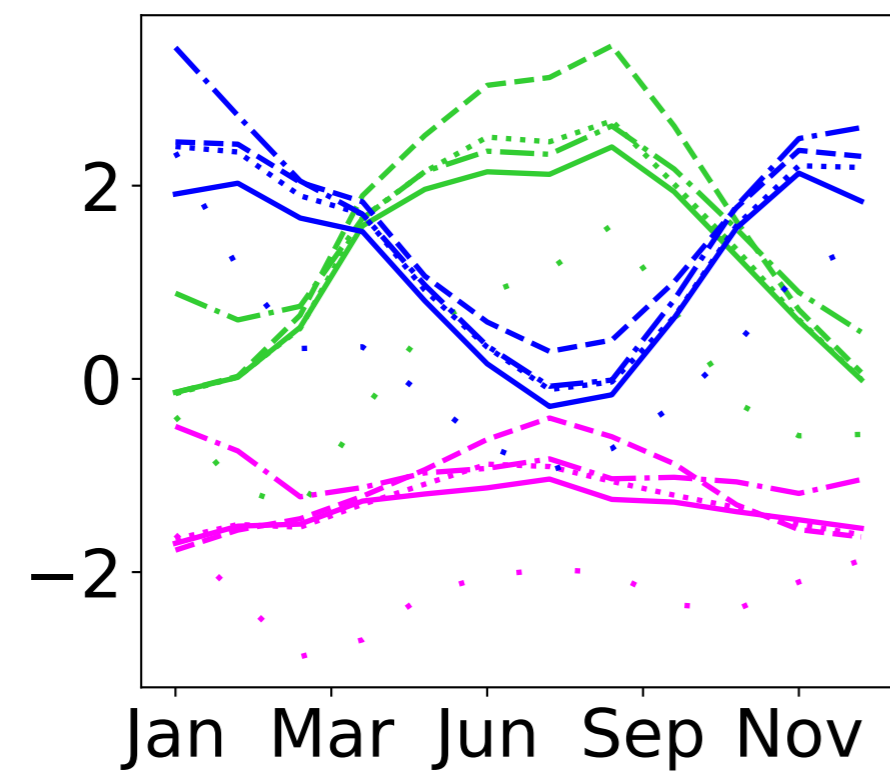

Munich

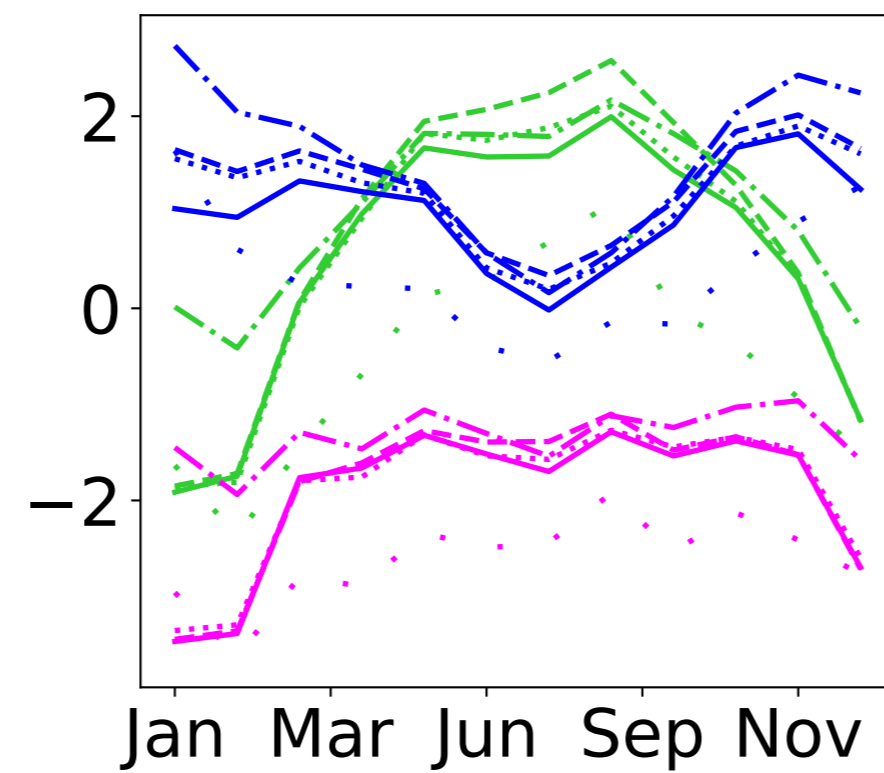

Prague

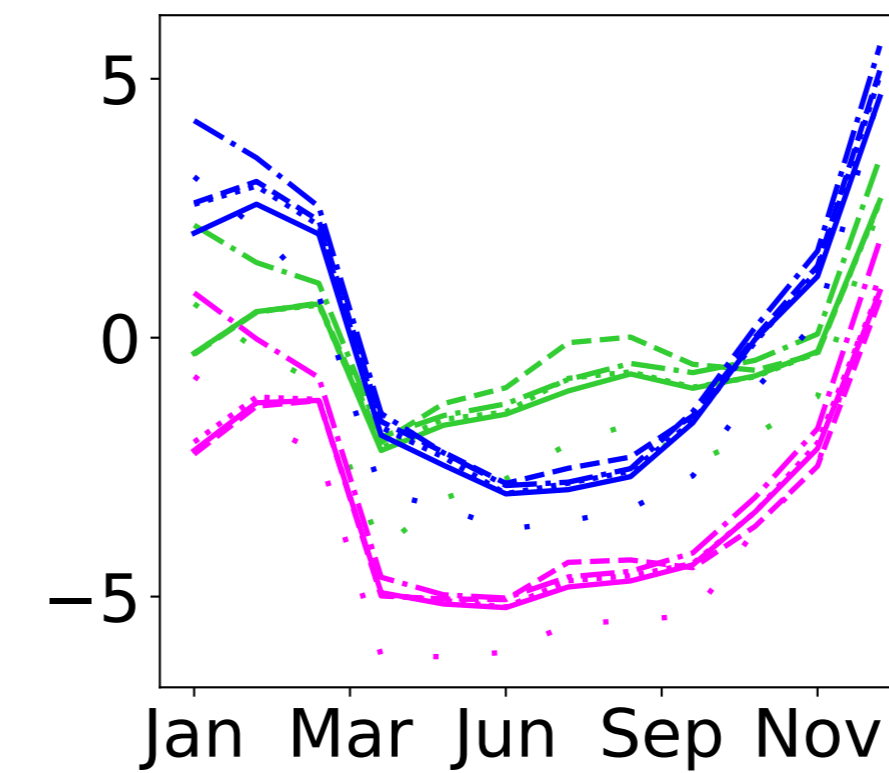

Vienna

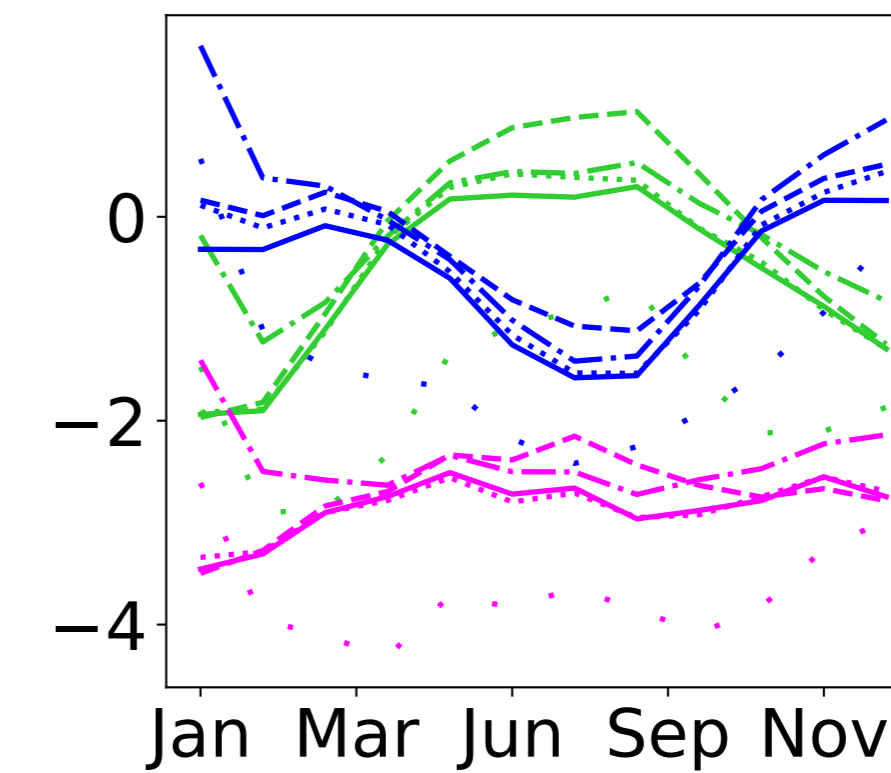

Warsaw

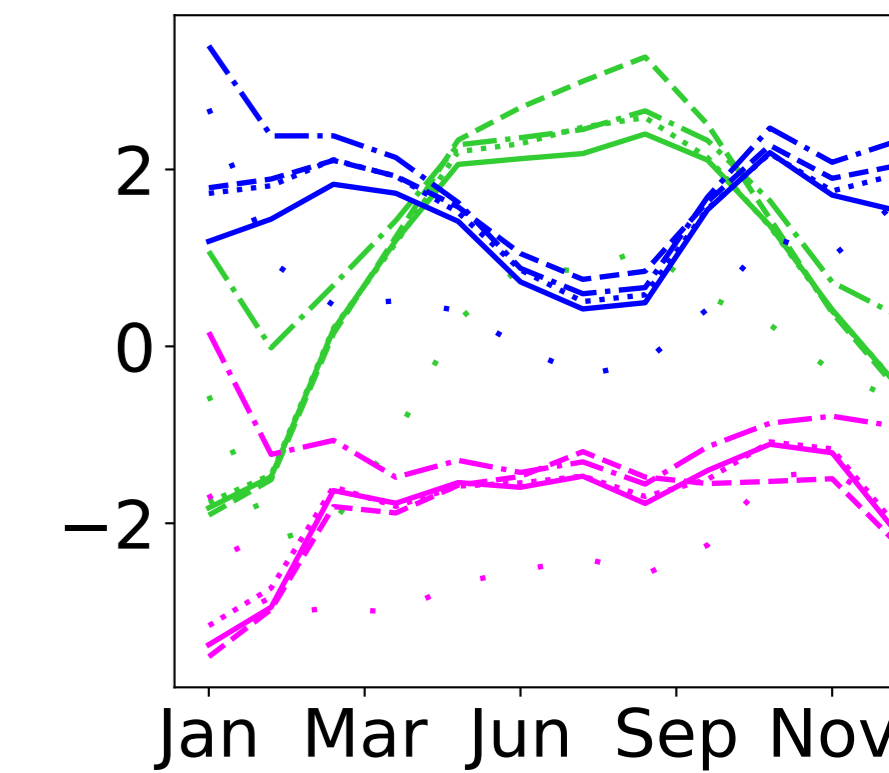

Vicinity

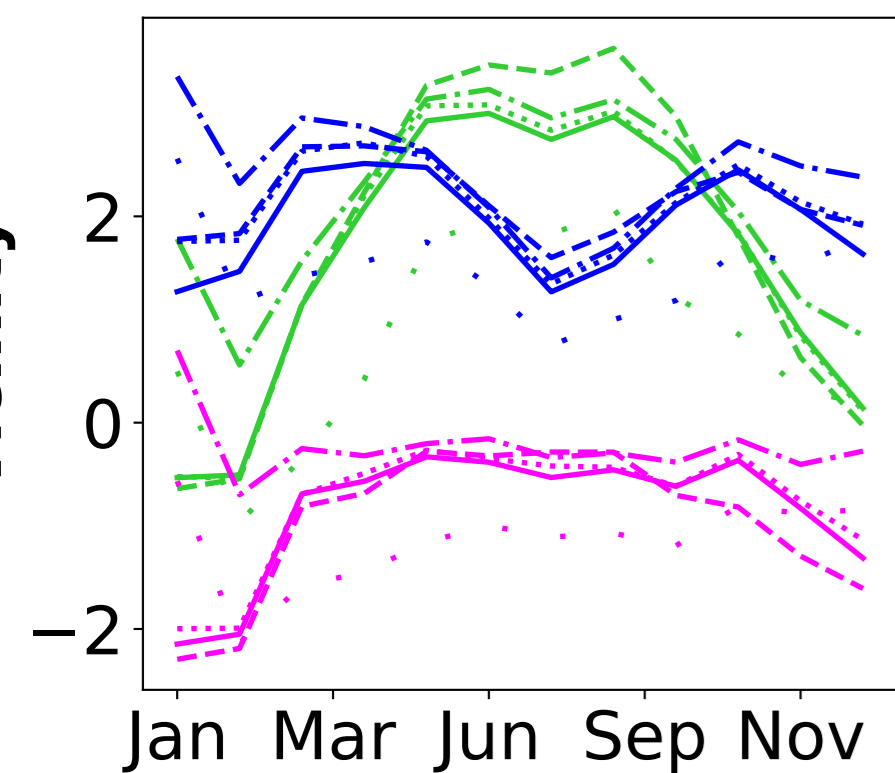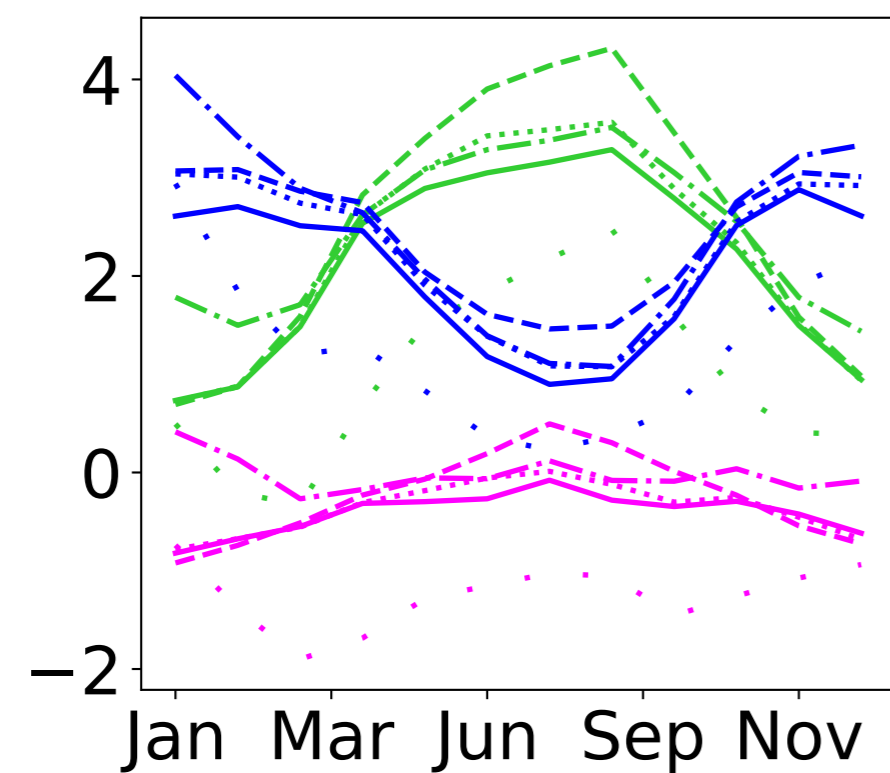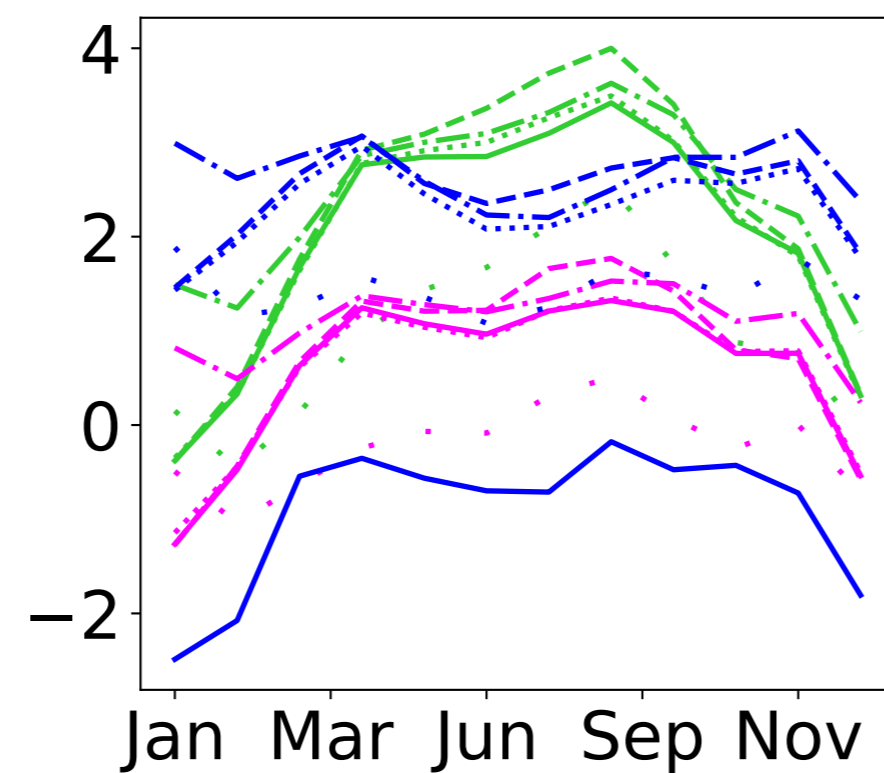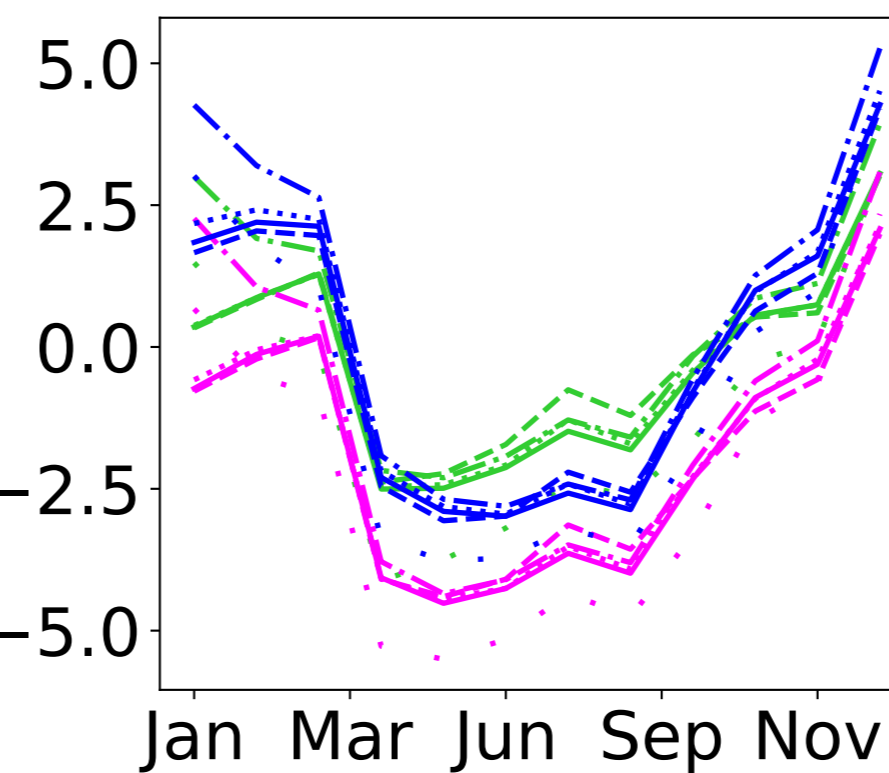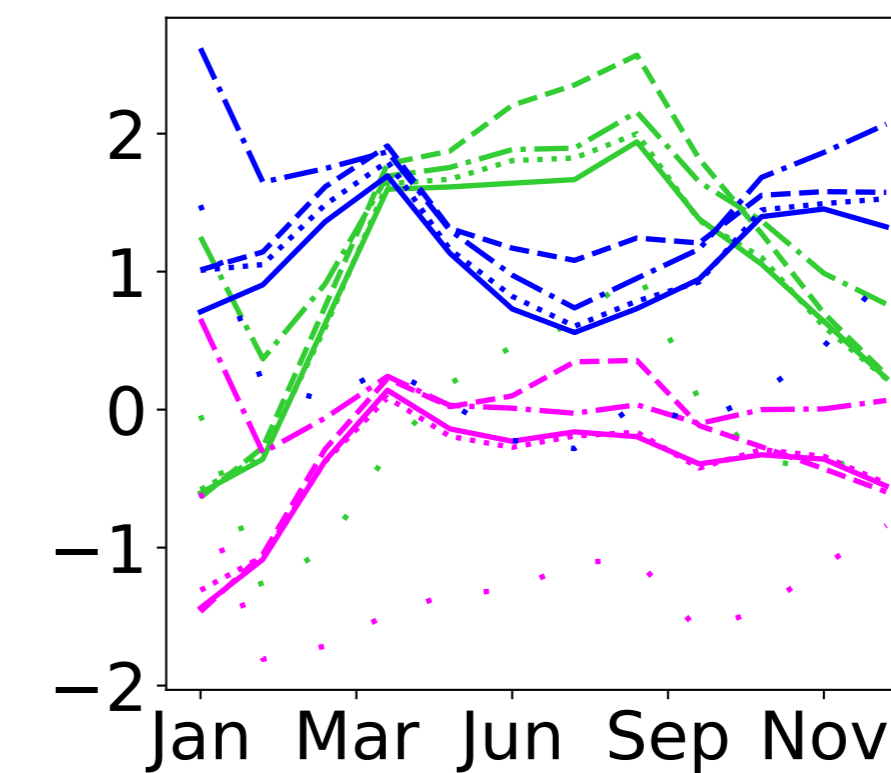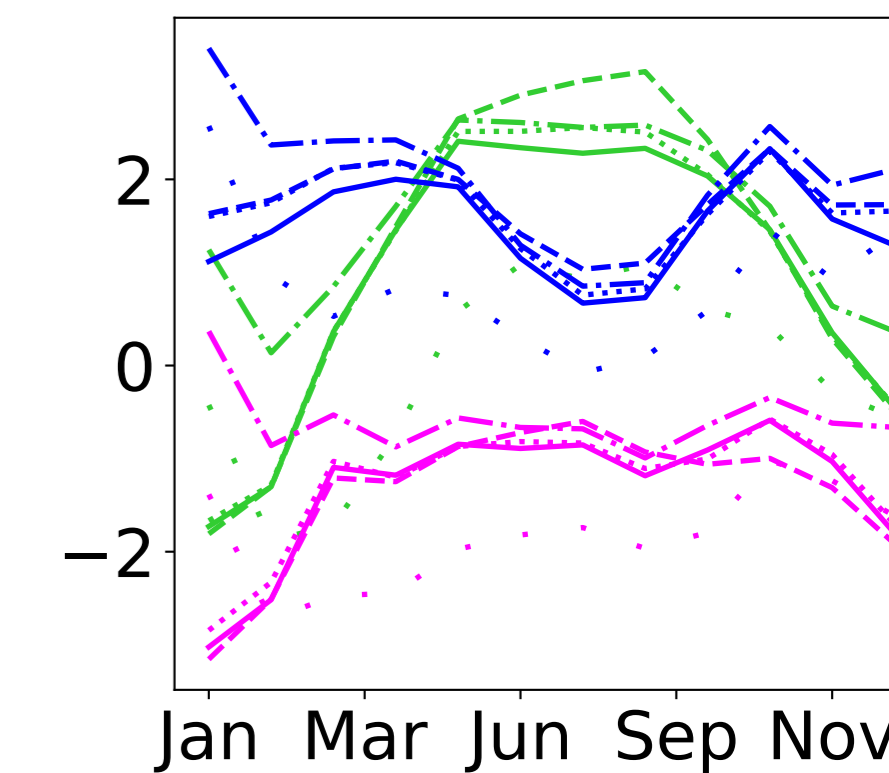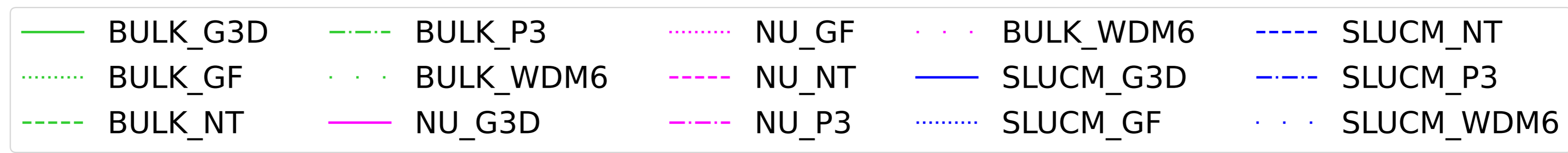

Supplement: Supplementary file 2 — Data S1 [file NYAS-1553-461-s001.zip › Comparison_obs_sims_T2min.pdf]

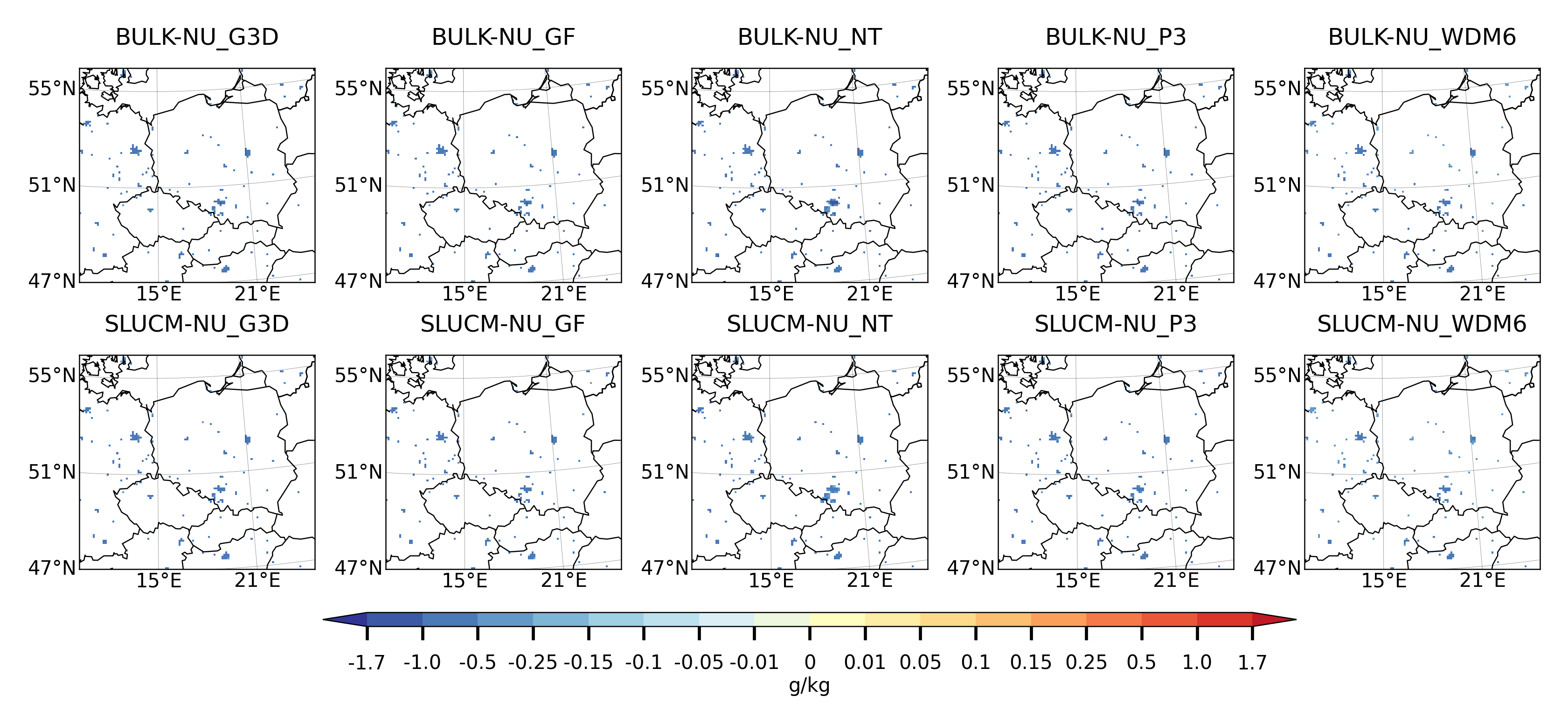

Supplement: Supplementary file 2 — Data S1 [file NYAS-1553-461-s001.zip › t-student_Q2_JJA.png]

# Land Use Categories

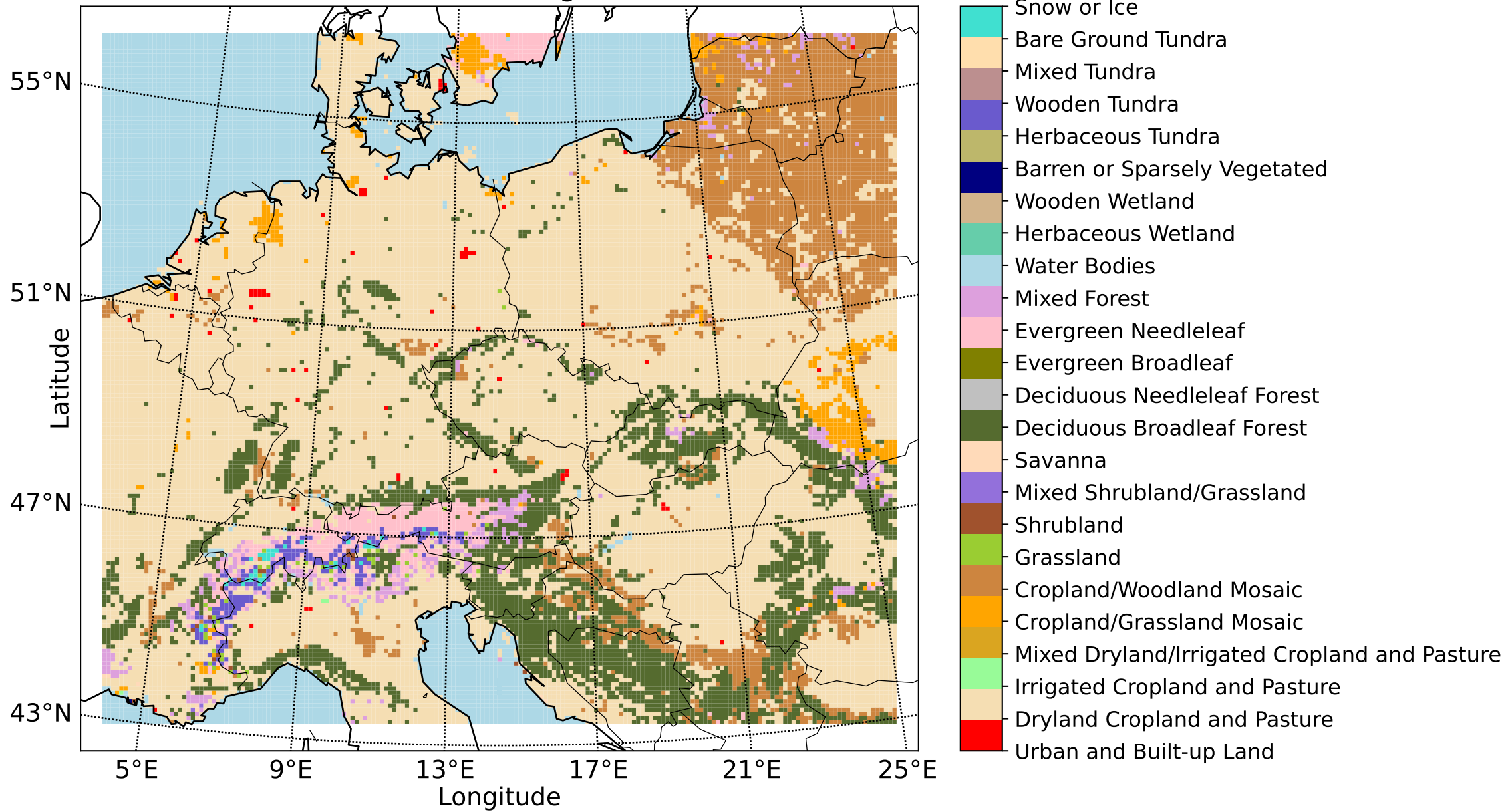

Supplement: Supplementary file 2 — Data S1 [file NYAS-1553-461-s001.zip › usgs_land_use_categories_with_basemap.pdf]

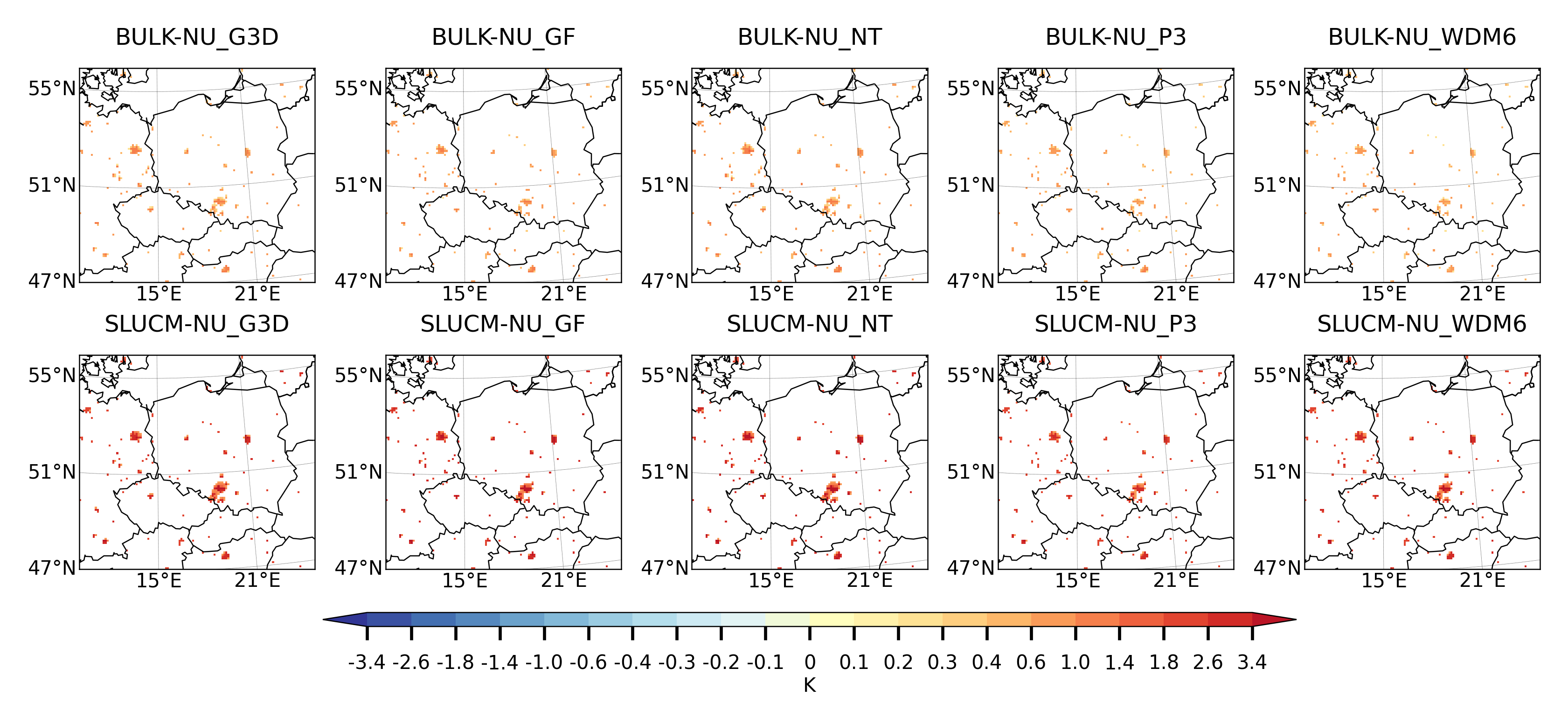

Supplement: Supplementary file 2 — Data S1 [file NYAS-1553-461-s001.zip › t-student_T2_DJF.png]

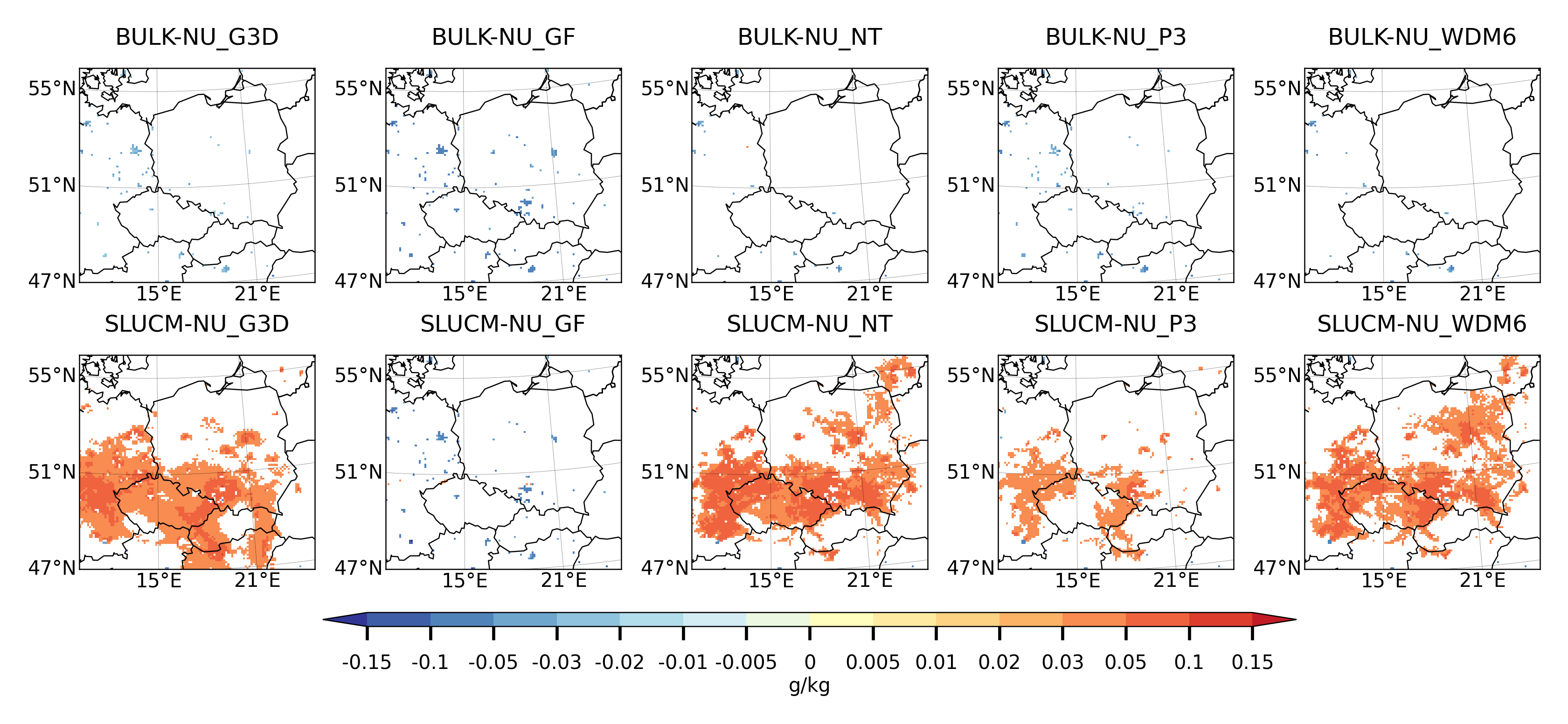

Supplement: Supplementary file 2 — Data S1 [file NYAS-1553-461-s001.zip › t-student_Q2_DJF.png]

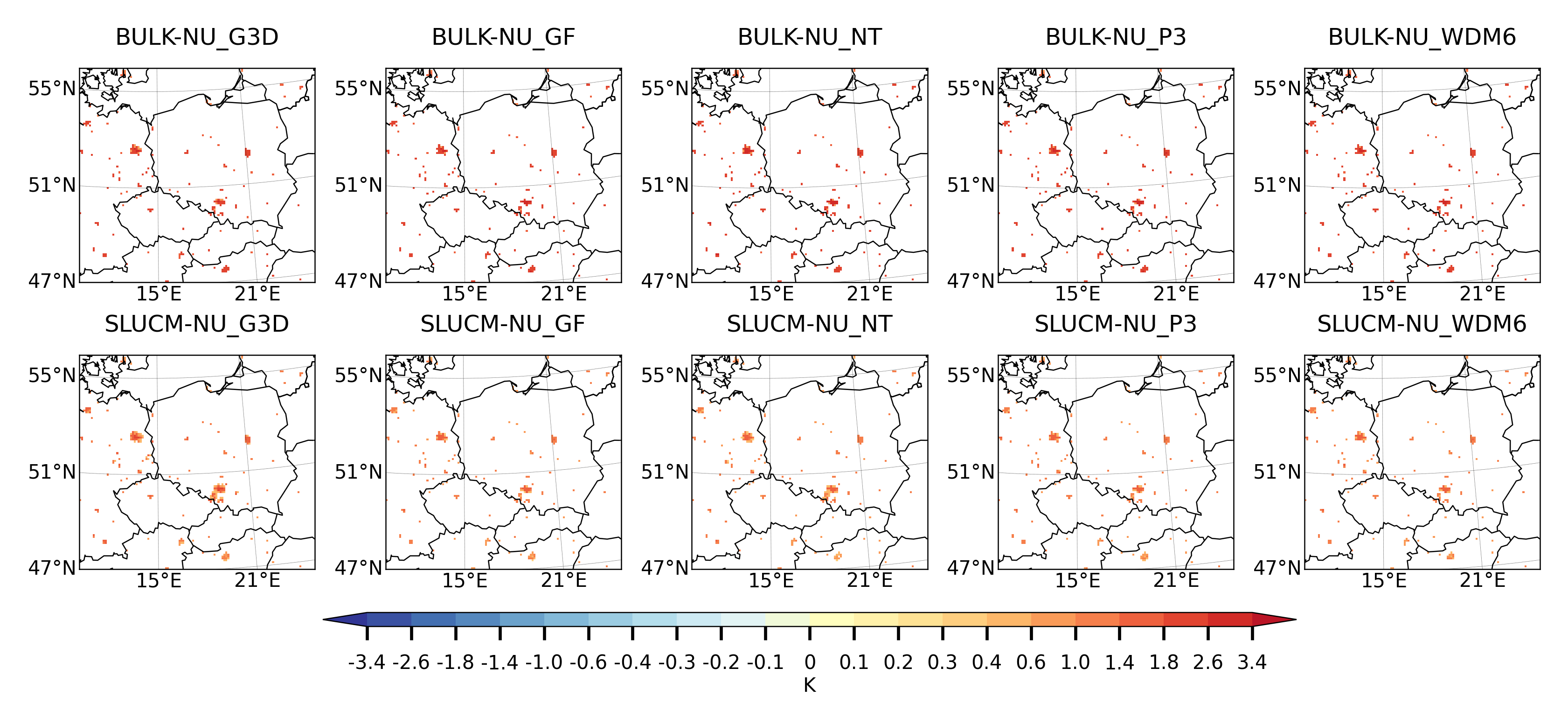

Supplement: Supplementary file 2 — Data S1 [file NYAS-1553-461-s001.zip › t-student_T2_JJA.png]
